# Translation arrest cancellation of VemP, a secretion monitor in *Vibrio*, is regulated by multiple *cis* and *trans* factors, including SecY

**DOI:** 10.1016/j.jbc.2024.107735

**Published:** 2024-09-02

**Authors:** Yuki Ikeda, Ryoji Miyazaki, Tomoya Tsukazaki, Yoshinori Akiyama, Hiroyuki Mori

**Affiliations:** 1 Institute for Life and Medical Sciences, Kyoto University, Kyoto, Japan; 2 Division of Biological Science, Graduate School of Science and Technology, Nara Institute of Science and Technology, Nara, Japan

**Keywords:** nascent polypeptide, periplasmic chaperone, protein cross-linking, protein complex, proton motive force, ribosome, SecA, secretion

## Abstract

VemP is a secretory protein in the *Vibrio* species that monitors cellular protein-transport activity through its translation arrest, allowing expression of the downstream *secD2-secF2* genes in the same operon, which encode components of the protein translocation machinery. When cellular protein-transport function is fully active, *secD2/F2* expression remains repressed as VemP translation arrest is canceled immediately. The VemP arrest cancellation occurs on the SecY/E/G translocon in a late stage in the translocation process and requires both *trans* factors, SecD/F and PpiD/YfgM, and a *cis* element, Arg-85 in VemP; however, the detailed molecular mechanism remains elusive. This study aimed to elucidate how VemP passing through SecY specifically monitors SecD/F function. Genetic and biochemical studies showed that SecY is involved in the VemP arrest cancellation and that the arrested VemP is stably associated with a specific site in the protein-conducting pore of SecY. VemP-Bla reporter analyses revealed that a short hydrophobic segment adjacent to Arg-85 plays a critical role in the regulated arrest cancellation with its hydrophobicity correlating with the stability of the VemP arrest. We identified Gln-65 and Pro-67 in VemP as novel elements important for the regulation. We propose a model for the regulation of the VemP arrest cancellation by multiple *cis* elements and *trans* factors with different roles.

The cell surface of Gram-negative bacteria consists of two lipid bilayers, the cytoplasmic and outer membranes, and the periplasmic space in between, where many soluble and membrane proteins are localized. The outer membrane and periplasmic proteins are generally synthesized in the cytoplasm as precursors with an N-terminal signal sequence and then targeted to the SecY/SecE/SecG (SecY/E/G) channel, or Sec translocon, on the cytoplasmic membrane to initiate their membrane translocation. Since SecY/E/G is a passive channel, it requires both the essential ATP-driven motor SecA and the monovalent cation-driven motor SecD/SecF (SecD/F) for efficient protein transport (1, 2). SecY/E/G is not only involved in the transport of secretory proteins but also in the integration of cytoplasmic membrane proteins. For the latter, it recognizes their hydrophobic transmembrane (TM) segments and directs them into the lipid phase (see below).

SecY, the central component of the Sec translocon, is a membrane protein with 10 TMs (hereafter referred to as TM1–TM10 from the N terminus) (3). It exhibits a bivalve-like overall structure formed by the N- and C-terminal halves, each containing 5 TMs, which has an hourglass-shaped internal tunnel with a small hole formed by "pore ring" residues (4, 5). In the resting state, a short α-helix called "plug helix" is placed on the pore ring from the periplasmic side to prevent leakage of small molecules. At the posterior of the SecY bivalve, the two halves of SecY are tethered upon the interaction of TM3 of SecE, whereas at the anterior, TM2 and TM7 form a lateral gate that allows hydrophobic signal sequences of secretory proteins and TM regions of membrane proteins to be released into the lipid phase. In the initial step of translocation, a signal sequence intercalates into the lateral gate, which widens the channel and displaces the plug helix from the pore ring, resulting in the opening of the hole (4). The mature region of the secretory protein is sequentially transferred to the periplasm through the hole. Signal sequences are cleaved by leader peptidase on the periplasmic during or shortly after the translocation (6).

SecA binds to a cytoplasmic protrusion in the C-terminal half of SecY (7, 8) and plays an essential role in the initial step of translocation (9). It also mediates the forward movement of a substrate protein through SecY from the cytoplasmic side; however, the function of SecA in this process remains controversial (10, 11). In contrast, SecD/F is a hetero-dimeric membrane protein complex, both of which have 6 TMs and a large first periplasmic (P1) domain (12). The P1 domain of SecD consists of two subdomains (head and base), with the former exhibiting a substrate-binding pocket (13). We proposed that SecD/F pulls up a substrate polypeptide from the periplasmic side by repeated rigid-body movements of the P1 head-subdomain coupled with the influx of monovalent cations, driven by the proton/cation motive force (14, 15). SecD/F can complete the translocation reaction independently of SecA (14). Thus, SecA and SecD/F, acting from the respective sides of SecY/E/G, are thought to independently drive the early and late stages of translocation, respectively.

Bacteria possess a mechanism to sense their own protein-transport activity and maintain it by upregulating the expression of Sec-related factors when their protein-transport activity decreases (16). Monitoring substrates encoded by genes upstream of *sec*-related genes play a central role in this regulation. They are secretory or membrane proteins that monitor the cellular protein-transport activity by sensing the state of their own transport. When the transport of a monitoring substrate is compromised, its translation is stably arrested near its C terminus. As a result, secondary structures of its mRNA formed in the intergenic regions between the monitoring substrate gene and the downstream *sec*-related genes are disrupted by the stalled ribosome, leading to the exposure of the SD sequence (present in the intragenic region) for the downstream genes and their translation. In contrast, if the cellular protein-transport function remains fully active, the translation arrest is transient and is quickly canceled by the pulling forces that drive its translocation. The translation arrest and its proper translocation-coupled translation arrest cancellation (TTAC) enable the real-time feedback regulation of the Sec-related proteins (16). *Escherichia coli* SecM (17), *Vibrio alginolyticus* VemP (18), and *Bacillus subtilis* MifM (19) are the monitoring substrates that regulate the expression of SecA, SecD2/F2, and YidC2, a membrane protein insertase (20), respectively. Recent comprehensive sequence information-based searches against a wide variety of bacterial genomes combined with biochemical analysis revealed that many secretory (or membrane) proteins encoded by genes located upstream of the *secA*, *secD/F*, and *yidC* homologs can undergo translation arrest (21, 22), suggesting that the mechanism underlying the translation arrests of the monitoring proteins is widely employed for maintaining protein export activity in bacteria.

*Vibrio* species, which are closely related to *E*. *coli*, possess two SecD/F paralogs with different ion specificity: SecD1/F1 utilizes Na^+^ as the driving ion (14) and is constitutively expressed, whereas SecD2/F2 is a putative H^+^-driven motor whose expression is tightly regulated by the translation arrest of VemP (18). When VemP translocation is impaired in an environment such as a Na^+^ poor condition where Na^+^-driven SecD1/F1 function is reduced, specific interactions between the arrest motif of VemP and the inner wall of the ribosome exit tunnel mediate stable translation arrest of VemP (23, 24) and upregulation of H^+^-driven SecD2/F2 (18), resulting in recovery of the cellular protein transport activity. The *vemP-secD2/F2* operon is specifically conserved among *Vibrio* species, but it is not present in *E. coli* (18). Since the expression level of SecD2/F2 correlates well with the extent of the VemP arrest, even when an *E.coli* strain with a plasmid carrying *vemP-secD2/F2* is used (18), no *Vibrio*-specific *trans-*factor is required for the regulatory mechanism and the results obtained with *E. coli* cells would essentially reflect the regulation mediated by VemP in *Vibrio* cells. Pulse-chase experiments with *E. coli* cells expressing VemP revealed that the arrest cancellation occurs on SecY/E/G after the signal sequence cleavage in a late step of translocation (24). *In vivo* photo cross-linking (XL) studies with *E. coli* cells showed that a nascent VemP polypeptide interacts with a membrane-anchored periplasmic chaperone PpiD as well as SecY and SecG, and the P1 head subdomain of SecD establishes a contact with PpiD (25, 26) (Fig. 1*A*). In addition, we have shown that PpiD forms a stable heterodimer with YfgM, a membrane protein of unknown function, and that the complex formation is required for these proteins to interact with both SecY/E/G and SecD/F (27) (Fig. 1*A*). We proposed that the cooperative functioning of SecD/F and PpiD/YfgM is required for the normal VemP TTAC, because either reduction of the cellular amount of SecD/F (in an *E. coli secD1* mutant) or disfunction of PpiD/YfgM (in an *E. coli* Δ*ppiD* or Δ*yfgM* mutant) stabilizes the VemP-arrested state, and overexpression of one complex in *E. coli* cannot compensate for disfunction of the other (25, 27). Using the *Vibrio secD2/F2, ppiD, or yfgM* deletion strains, we have obtained the consistent results, supporting the proposal (25, 27). VemP would specifically monitor late-stage protein-transport activities, consistent with its physiological function to regulate the SecD2/F2 expression. Furthermore, the Arg-85 residue in VemP should plays an essential role in the proper VemP TTAC, because the replacement of Arg-85 by any of six other amino acid residues with different side chain chemical properties including Lys (another positively charged residue) and Trp destabilizes the arrested state of VemP (25). However, the molecular mechanisms underlying the monitoring of transport activity by VemP during later stages, the exact roles of the *trans* factors (SecD/F and PpiD/YfgM), and the contribution of the *cis* element, Arg-85 in VemP, to this process remain unclear.

**Figure 1:**
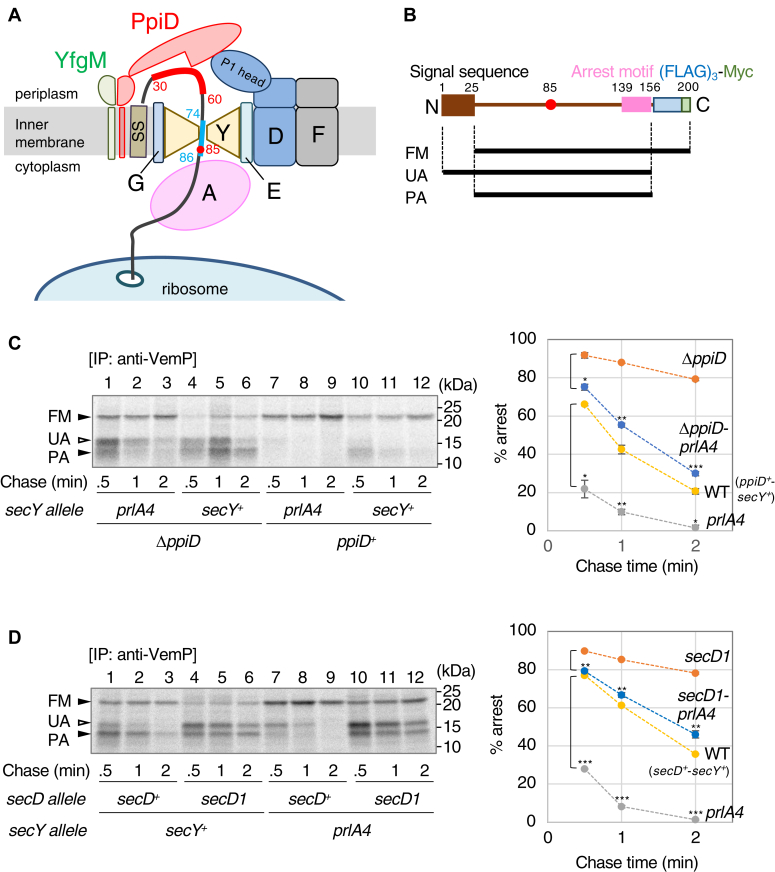
**The *prlA4* mutation alleviates the defect in VemP TTAC attributed to either Δ*ppiD* or *secD1* mutation.***A*, schematic of VemP nascent chain passing through the SecY/E/G translocon. SS, A, Y, E, G, D, F, and P1 head represent the signal sequence of VemP, SecA, SecY, SecE, SecG, SecD, SecF, and the P1 head subdomain in SecD, respectively. SecD/SecF and the PpiD/YfgM complex are cellular components involved in the VemP TTAC (25, 27) as *trans* factors. *Red* and *pale blue thick lines* in the VemP nascent chain indicate PpiD- and Sec translocon-interaction regions, respectively, identified by *in vivo* XL experiments (25). The *red circle* means Arg-85 residue, a *cis* element responsible for the proper VemP TTAC (25). *B*, a schematic structure of a model substrate, VemP-F_3_M, was used for pulse-chase experiments. FM, UA, and PA observed in C and D represent the full-length mature, unprocessed arrested, and processed arrested bands of VemP, respectively. *C* and *D*, cells of the indicated mutant strain carrying p-*vemP-f*_*3*_*m* were grown at 30 ˚C in M9 based medium until the early log phase. The cells were induced with 1 mM IPTG and 2 mM cAMP and pulse-labeled with [^35^S]-methionine for 30 s followed by a chase experiment with an excess amount of cold methionine for the indicated periods. At each time point, total cellular proteins were acid-precipitated, subjected to immunoprecipitation (IP) with anti-VemP antibodies, and analyzed by SDS-PAGE and phosphor-imaging. The result shown is a representative of two biological replicates. The intensities of these three bands were quantified, and the percentage of the arrested products to total VemP was calculated according to the formula: [% arrest] =([UA] + 2.5 × [PA])/([UA] + 2.5 × [PA] + 2.5 × [FM]) × 100. Means of the % of the arrested form of VemP were plotted against the chase time (error bars are SD (N = 2)). *Right graphs*, an unpaired two-tailed Student's *t* test was used to statistically compare the values between the groups. ∗*p* < 0.05, ∗∗*p* < 0.01, and ∗∗∗*p* < 0.001. PA, processed arrested form; TTAC, translocation-coupled translation arrest cancellation; XL, cross-linking.

In this study, we aimed to address the aforementioned questions using genetic and biochemical approaches with *E.coli* for which a wide variety of useful resources are available. We demonstrated that SecY is involved in the proper TTAC and characterized the mode of interaction between VemP and SecY in its arrested state. In addition, we developed a reporter system to easily evaluate the arrested state of VemP and identified a short hydrophobic segment (SHS) (L^78^ALF) and the (hydrophilic)xP motif in VemP as novel *cis* elements involved in the proper TTAC along with other *cis* and *trans* factors. Based on these results, we aim to propose a molecular mechanism by which VemP can specifically monitor the SecD/F function.

## Results

### SecY is involved in the proper TTAC of VemP

The findings that the TTAC of VemP occurs on the SecY/E/G translocon (24) and that the region from residue 74 to 86, containing Arg-85, of nascent VemP transiently interacts with SecY (25) (Fig. 1*A*) raise the possibility that the Sec translocon participates directly in the TTAC. To test this, we examined the effect of the *prlA4* mutation (*prlA4* is an allele of *secY*), which restores transport of a preprotein with a defective signal sequence (28), on the stability of arrested VemP. Strains carrying a combination of the *prlA4* mutation and either the *ΔppiD* mutation or the *secD1* mutation, which decreases the cellular accumulation level of SecD/F to undetectable levels, were constructed (see Experimental procedures) and characterized (Fig. S1, *A*–*C*, Supporting result 1). VemP-F_3_M, a fusion protein with C-terminal 3xFLAG-Myc tags (Fig. 1*B*), was expressed in these strains, and the kinetics of its TTAC was examined by pulse-chase experiments (Fig. 1, *C* and *D*). The arrested (unprocessed arrested form [UA]; processed arrested form [PA]) and full-length mature (FM) proteins were clearly distinguishable on neutral SDS-PAGE (Fig. 1, *C* and *D*; (24)). To assess TTAC, these three bands were quantified, and the percentage of the arrested products in total VemP was calculated (Fig. 1, *C* and *D*, right graphs). The arrested forms (UA and PA) were gradually converted to the FM form possessing the C-terminal F_3_M tag, coupled with the translocation over time in the WT strain (yellow), whereas they were more stable in the Δ*ppiD* (Fig. 1*C*, orange) and *secD1* (Fig. 1*D*, orange) strains, as reported previously (25). Interestingly, the introduction of *prlA4* destabilized the VemP arrest (blue *versus* orange and gray *versus* yellow). Consistent with these results, *prlA4* increased the PhoA activity in cells expressing a *vemP-phoA* reporter (27) (Fig. S2). These results strongly implicate SecY in the VemP TTAC.

### Mode of interaction between the arrested VemP and SecY

**Figure 2:**
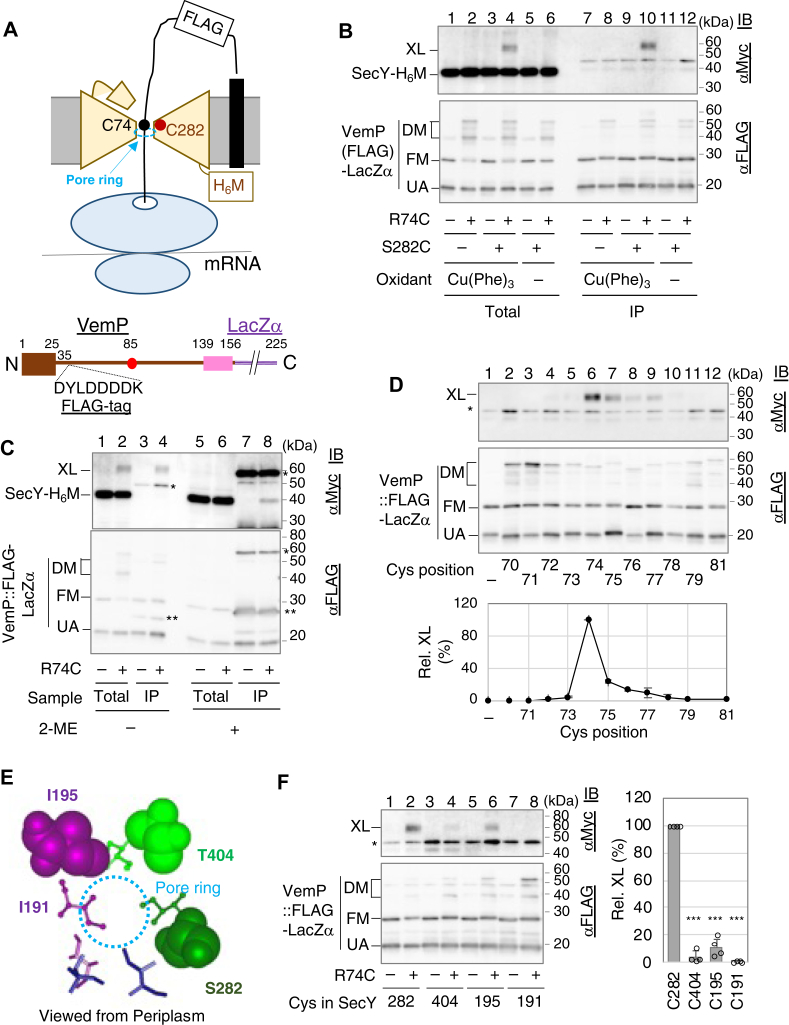
***In vivo* disulfide (S-S) bond formation between SecY(Cys)-H**_**6**_**M and the translation arrested VemP::FLAG(Cys) in Δ*ppiD* strain.***A*, a schematic representation of the arrested state of a nascent VemP polypeptide in the SecY. The positions of Cys282 in SecY-H_6_M and Cys74 in the arrested VemP::FLAG are shown in the *cartoon*. A schematic structure of VemP::FLAG-LacZα is also shown. *B*, *in vivo* S-S bond formation between SecY(C282) and the arrested VemP::FLAG (C74). Δ*ppiD* cells carrying both p-*secY(S282 or C282)-h*_*6*_*m-SD-ha-secE* and p-*vemP::FLAG(R74 or C74)-lacZα* were grown in the L-medium at 30 ˚C until the midlog phase. The cells were then induced with 1 mM IPTG and 2 mM cAMP for 1 h. Cultures were subjected to an oxidation reaction with final 0.25 mM Cu(Phe)_3_ on ice for 30 min or mock treatment (−). Acid-precipitated total proteins were solubilized with a 1% SDS-containing buffer without 2-mercaptoethanol. A portion of the solubilized samples was directly analyzed by SDS-PAGE and immunoblotting (IB) with anti-Myc and anti-FLAG antibodies (Total). Another portion of the samples was subjected to IP with anti-FLAG M2 beads. Isolated proteins were analyzed by SDS-PAGE and IB with the same antibodies (IP). XL, DM, FM, and UA on the *left side* of the *lower gel* represent the cross-linked product, the dimer forms of both VemP::FLAG-LacZα and the arrested VemP::FLAG, the full-length mature form of VemP::FLAG-LacZα, and the unprocessed arrested VemP::FLAG product, respectively. The dimer form of the arrested VemP::FLAG could be generated in the cytoplasm, because it was expected that a portion of the arrested VemP was not targeted to the translocon. The result shown is a representative of two biological replicates. *C*, reducing reagent sensitivity of the XL products. Δ*ppiD* cells expressing SecY(C282)-H_6_M, HA-SecE, and VemP::FLAG(C74 or R74)-LacZα were treated with the oxidant a and analyzed by SDS-PAGE and IB either directly (Total) or after purification by IP with the anti-FLAG M2 beads (IP) as shown in Figure 2*B*. Prior to SDS-PAGE, SDS-solubilized samples were divided into two portions. One sample was treated (+) with the final 10% (v/v) 2-mercaptethanol at 37 ˚C for 10 min, and the other was left untreated (−). ∗ and ∗∗ presumably represent H and L chains of antibodies nonspecifically cross-reacting with anti-Myc antibody, respectively. The mobility change of the antibody L chains on SDS-PAGE after treatment with the reducing reagent is probably due to the difference in its molecular shape of the L chain with or without intramolecular S-S bonds. The result shown is a representative of two biological replicates. *D*, effects of Cys positions in VemP on S-S bond formation between SecY(C282) and VemP::FLAG(mono-Cys). Δ*ppiD* cells expressing SecY(C282)-H_6_M, HA-SecE, and VemP::FLAG (Cys at the indicated positions)-LacZα were treated with the oxidant and subjected to IP using anti-FLAG antibodies as shown in Figure 2*B*. Acid-precipitated total proteins were solubilized with the buffer containing 1% SDS without the reducing reagent and analyzed by SDS-PAGE and IB with anti-Myc and anti-FLAG antibodies. ∗ indicates a protein nonspecifically cross-reacting with anti-Myc antibody. The result shown is a representative of two biological replicates. The intensities of the XL bands were quantified and normalized to those of the accumulated VemP polypeptides (UA + FM). The XL efficiency was normalized to that of the VemP::FLAG(C74), and means with SD (N = 2) are shown in the *bottom graph*. The dimerization efficiency seemed to be influenced by the positions of introduced Cys residues for unknown reasons. *E*, *close-up view* of the pore ring of the periplasmic side of an *E. coli* SecY model structure (UniProt: A0A827V5K4) obtained from the AlphaFold Protein Structure Database (https://alphafold.ebi.ac.uk/)). Only six Ile residues that form the pore ring in SecY are shown as either *stick* or *ball-and-stick*, in which the residues shown as *ball-and-stick* have previously been shown to form S-S bonds with a nascent Cys-containing secretory protein when replaced with Cys (29). Three residues (I195, S282, and T404) substituted with Cys in this study are represented as space fills. These residues are located at a similar depth to the membrane surface. *F*, effects of the Cys positions in SecY-H_6_M on the S-S bond formation between VemP::FLAG(C74). Δ*ppiD* cells expressing SecY(mono Cys at the indicated position)-H_6_M, HA-SecE, and VemP::FLAG(C74 or R74)-LacZα were treated with the oxidant and subjected to IP with anti-FLAG M2 beads as shown in Figure 2*D*. ∗ indicates a protein nonspecifically cross-reacting with anti-Myc antibody. The result shown is a representative of two biological replicates. The intensities of the XL products were quantified and normalized as described in Figure 2*D*. The mean values of the XL efficiency normalized to that of the SecY(C282)-H_6_M are shown with SD (N = 4) and the raw data in the *right graph*. A paired two-tailed Student's *t* test was used to statistically compare the values between the WT and each mutant. ∗∗∗*p* < 0.001. Amp, ampicillin; FM, full-length mature; HA, hemagglutinin; IP, immunoprecipitation; UA, unprocessed arrested form; XL, cross-linking.

To dissect the potential role of SecY in the VemP TTAC, we examined the interaction between the arrested VemP and SecY by disulfide (S-S) bond XL experiments using a SecY-H_6_M (C terminally His_6_-Myc–tagged SecY) and hemagglutinin (HA)-SecE (N terminally HA-tagged SecE) coexpression system (Fig. S3, *A* and *B*). We first used an *E. coli* SecY-H_6_M derivative (SecY(C282)-H_6_M) carrying a single Cys at position 282 located near the pore ring, because Cys-282 in SecY has been shown to form an S-S bond with Cys introduced into a translocating polypeptide (29) (Fig. 2*A*, upper). Using the VemP-PhoA reporter, we confirmed that SecY(C282)-H_6_M retained a comparable ability to maintain the VemP-arrested state in the WT and Cys-less SecY-H_6_M (Fig. S3*C*, Supporting result 2). We also performed Cys-scanning mutagenesis targeting 25 residues (65–89) in the VemP portion of the VemP-PhoA reporter and found that these Cys substitutions, except Q65C, P67C, and R85C, had a minor effect on VemP translation arrest (Fig. S4*A*).

We used VemP::FLAG-LacZα, a VemP-derivative having a FLAG sequence (DYLDDDDK) between Pro-35 and Tyr-36 and a C terminally fused LacZα moiety, in the following XL experiments (Fig. 2*A*, lower). The internal FLAG tag, which did not affect the kinetics of the VemP TTAC (Fig. S4*B*), allowed efficient isolation of this model protein and its XL products with anti-FLAG antibodies. To induce S-S formation, Δ*ppiD* cells coexpressing SecY(C282)-H_6_M and VemP::FLAG(mono Cys)-LacZα were treated with a membrane-permeable oxidant, Cu(Phe)_3_, (Fig. 2*B*). Among the mono-Cys derivatives of VemP::FLAG-LacZα, the one carrying C74 produced a slowly migrating band on SDS-PAGE that was detected by anti-Myc immunoblotting (IB) in both total (lane 4) and anti-FLAG immunoprecipitated (IP) (lane 10) samples. The formation of this band required both Cys residues in SecY and VemP and oxidant treatment, (Fig. 2*B*). Further treatment of the oxidized samples with 2-mercapto-ethanol resulted in the disappearance of this band (Fig. 2*C*, lane 6) and the concomitant appearance of an anti-Myc–reactive band of the size of SecY-H_6_M in the IP sample (Fig. 2*C*, lane 8). Together, these results indicate that the slowly migrating band represented an S-S XL product between SecY(C282)-H_6_M and VemP::FLAG(C74). Anti-FLAG IB failed to detect this band (Fig. 2*B*, lower gel), probably because only a very small fraction of the VemP::FLAG(C74) molecules were cross-linked with SecY(C282)-H_6_M. The findings that (i) this XL product was RNase-sensitive (Fig. S5*A*, Supporting result 3–(1)) and (ii) the introduction of R85W drastically reduced XL formation (Fig. S5*B*, Supporting result 3–(2)) strongly suggest that the observed XL product contained the nascent VemP::FLAG polypeptide.

Systematic XL experiments with SecY(C282)-H_6_M and VemP::FLAG derivatives carrying mono-Cys at different positions (70–81) revealed that XL products were produced in both purified (Fig. 2*D*) and total (Fig. S5*C*) samples but only with VemP::FLAG carrying Cys at the positions of Arg-74 and a few downstream residues, suggesting that translocation of the nascent VemP polypeptide stops with its region around Arg-74 retained near the SecY pore ring. We next examined whether VemP::FLAG(C74) was disulfide cross-linked with other SecY mutants carrying Cys near the pore ring. We chose Cys at Ile-195 and Thr-404, which are at approximately the same depth in the membrane as Ser-282 (Fig. 2*E*, space filling), and Cys at Ile-191 (Fig. 2*E*, ball and stick), which is slightly cytoplasmic but forms an S-S bond with a translocating substrate *in vitro* (29). Analysis using the VemP-PhoA reporter confirmed that these SecY(Cys)-H_6_M mutants retained the ability to maintain the VemP-arrested form (Fig. S3*C*). XL of these three SecY(Cys)-H_6_M mutants with the nascent VemP::FLAG(C74) was much less efficient than that of SecY(C282)-H_6_M (Fig. 2*F*). In addition, essentially no XL was detected between these mutants and VemP::FLAG carrying mono-Cys at 73 to 77 positions (Fig. S5*E*). These results suggest that the nascent VemP stalling site in SecY is located adjacent to TM7 (lateral gate side), which contains Ser-282.

### A SHS, a novel *cis* element, was involved in maintaining the stability of the VemP translation arrest

**Figure 3:**
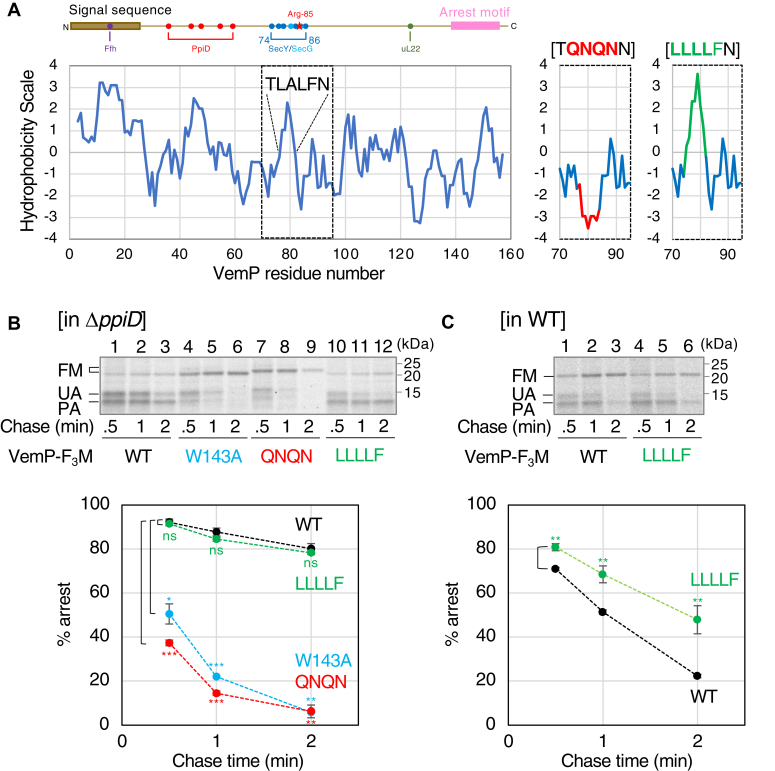
**A short hydrophobic segment in the Sec-interacting region of VemP is involved in the arrest cancellation.***A*, a schematic representation of VemP is shown at the *top* with the interaction sites with cellular components indicated (25). The hydrophobicity of VemP is plotted against the residue number using the “Kyte and Doolittle” scale (window size is 5) (47) (*left*). A short hydrophobic segment (SHS) whose hydrophobicity is highly conserved among *Vibrio* species in the Sec-interacting region (*blue line* in the schematic picture) is highlighted with the amino acid sequence TLALFN. The hydrophobicity of the region in the VemP derivatives (TQNQNN and LLLLFN) corresponding to the 70 to 95 region marked by the *dashed line* in the WT is also shown (*right*). The regions with hydrophobicity altered by these mutations are colored in *red* and *green*, respectively. *B*, pulse-chase experiments of Δ*ppiD* cells expressing VemP-F_3_M mutants. Δ*ppiD* cells carrying p-*vemP-f*_*3*_*m* with the indicated mutation were grown in the M9-based medium containing Amp at 30 ˚C until the early log phase. The cells were induced and subjected to pulse-chase experiments as described in Figure 1. The labeled proteins were purified using IP with anti-VemP antibodies and analyzed by SDS-PAGE and phosphor-imaging. The result shown is a representative of two biological replicates. The percentage of arrests was calculated as described in Figure 1. Means with SD (N = 2) are plotted against chase time. An unpaired two-tailed Student's *t* test was used to statistically compare the values between the WT and each mutant. ∗*p* < 0.05, ∗∗*p* < 0.01, ∗∗∗*p* < 0.001, and *ns*, not significant. *C*, pulse-chase experiments of WT cells expressing the indicated VemP-F_3_M derivatives were carried out, as shown in Figure 3*B*. The result shown is a representative of two biological replicates. Amp, ampicillin.

To gain insight into the role of SecY in the VemP TTAC, we focused on the VemP region (74–86) that transiently interacts with SecY when VemP translation is arrested (Fig. 3*A*, left panel). Hydrophobicity profile analysis of the corresponding regions in VemP orthologs revealed that they commonly contain a SHS (L^78^ALF in *V. alginolyticus* VemP) (Fig. S6, Supporting discussion 1). To explore the potential role of the SHS in TTAC, we first constructed two mutants, VemP(QNQN)-F_3_M and VemP(LLLLF)-F_3_M mutants, which exhibit an altered SHS with decreased and increased hydrophobicity, respectively (Fig. 3*A*, right panels), and examined the kinetics of their TTAC (Fig. 3*B*). Compared to WT VemP-F_3_M (black), the QNQN mutation (red) destabilized the arrest as did the strong arrest-defective mutation, W143A (24) (pale blue) in the Δ*ppiD* strain. Conversely, the LLLLF mutation (green) stabilized the arrest, and the TTAC of the LLLLF mutant was similar and slower than WT VemP-F_3_M in the Δ*ppiD* (Fig. 3*B*) and WT (Fig. 3*C*) strains, respectively. We then introduced these mutations into His_10_-VemP(ΔSS)-F_3_M, a VemP derivative lacking the signal sequence and thus defective in translocon binding, and examined the behavior of these mutants. In contrast to the arrest defective W143A mutant, the QNQN and LLLLF mutants as well as the R85W mutant accumulated in an arrested form (Fig. S7, Supporting result 4), indicating that the arrested forms of the latter mutants are stable unless they interact with the Sec translocon. These results strongly suggest that the SHS is involved in the VemP TTAC, where the reduced hydrophobicity of the SHS leads to facilitated arrest release.

### Establishment of a VemP-Bla reporter system enabling convenient monitoring of the translation arrest stability

**Figure 4:**
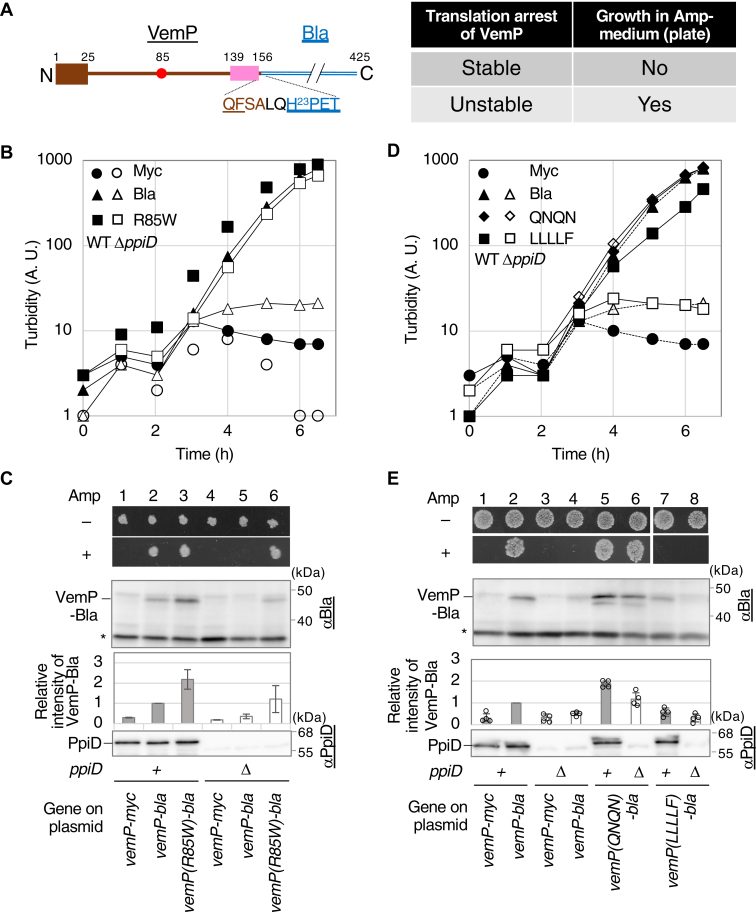
**Construction and evaluation of a VemP-Bla reporter system.***A*, a schematic representation of VemP-Bla including the amino acid sequence of the joint point (*left*) and the expected growth phenotypes (*right*). *B*, growth curves of either Δ*ppiD* or WT strain expressing a VemP derivative in Amp-containing medium. Cells of Δ*ppiD* (*open*) or WT (*closed*) strain carrying either p*-vemP-myc* (*circle*), p-*vemP-bla* (*triangle*), or p-*vemP(R85W)-bla* (*square*) were grown in the L-medium supplemented with both 10 μg/ml Amp, 20 μg/ml Cm, and 1 mM IPTG at 30 ˚C, and the turbidity of the cultures was measured every 1 h and plotted against cultivation time. The result shown is a representative of two biological replicates. *C*, *upper panels:* growth phenotype of either WT (1, 2, 3) or Δ*ppiD* (4, 5, 6) strains expressing either VemP-Myc, VemP-Bla, or VemP(R85W)-Bla on L-plate. Cells were grown in the L-Cm medium at 30 ˚C until midlog phase, diluted to 100-fold with saline, spotted on the L-plate supplemented with 1 mM IPTG, 20 μg/ml of Cm without (−) or with (+) 10 μg/ml Amp, and incubated at 30 ˚C for 16 h. The result shown is a representative of two biological replicates. *Lower panels:* cellular accumulation of VemP derivatives in either WT (1, 2, 3) or Δ*ppiD* (4, 5, 6) strains. Cells carrying a plasmid with the indicated gene were grown in the L-medium supplemented with 20 μg/ml Cm at 30 ˚C until midlog phase and induced with 1 mM IPTG for 1 h. Acid-precipitated total cellular proteins were solubilized in the SDS-sample buffer and analyzed by SDS-PAGE and IB using anti-Bla antibodies (*top column*) and anti-PpiD antibodies (*bottom column*). The result shown is a representative of two biological replicates. The intensities of VemP-Bla were quantified and normalized using a nonspecifically cross-reacting product (∗) as an internal control. The mean intensities of the VemP-Bla products relative to that of VemP-Bla (lane 2) expressed in the WT cells were shown in the *middle panel* with SD (N = 2). *D*, growth curves of either WT (*closed*) or Δ*ppiD* (*open*) strain expressing either VemP-Myc (*circle*), VemP-Bla (*triangle*), VemP(QNQN)-Bla (*rhombus*), or VemP(LLLLF)-Bla (*square*) in liquid culture. Cells of the indicated strains were cultured, and the turbidity of the cultures was measured as in B. The result shown is a representative of two biological replicates. E, *upper panels:* growth of either WT or Δ*ppiD* strain expressing the indicated VemP-Bla derivative on the L-plate, and (*lower panels*) cellular accumulation of the indicated VemP-Bla derivatives. All experiments were carried out as shown in *panel C*. The result shown is a representative of at least two biological replicates. The mean intensities of the VemP-Bla products relative to that of VemP-Bla (lane 2) expressed in the WT cells are shown in the *middle panel* with SD (N = 4) and the raw data. Amp, ampicillin; Cm, chloramphenicol.

To perform a systematic mutagenesis study for the SHS, we constructed a VemP-Bla reporter (Fig. 4*A*) in which the entire VemP sequence was followed by the Bla domain without the signaling sequence (aa 23–425). We expected that a significant amount of VemP-Bla would be synthesized and accumulated in the periplasm if the arrested state of VemP was destabilized but not under an arrest-stabilized condition. Since periplasmically accumulated Bla confers ampicillin (Amp) resistance to cells, the growth of the reporter-expressing cells in a medium containing an appropriate concentration of Amp will reflect the stability of the VemP arrest (Fig. 4*A*, right). Indeed, the model experiments described in the following demonstrated the usefulness of this system (Fig. 4, *B*–*E*). WT (closed) or Δ*ppiD* (open) strains carrying a plasmid encoding either VemP-Myc (circles), VemP-Bla (triangles), or VemP(R85W)-Bla (squares) were inoculated into the L-liquid medium containing 10 μg/ml Amp, and their growth was monitored by measuring culture turbidity (Fig. 4*B*). Cells carrying p-*vemP-myc* did not grow regardless of the host strain. In contrast, cells carrying p-*vemP-bla* grew when the WT strain was used as the host but not with the Δ*ppiD* host strain. This is logical because the VemP translation arrest is transient and properly released in the WT strain, whereas it is stabilized in the Δ*ppiD* strains. As expected, cells carrying p-*vemP*(*R85W*)-*bla*, which encodes an arrest-destabilizing mutant, grew well even with the Δ*ppiD* strain as the host. Consistent results were obtained when cells were grown on L-plates containing 10 μg/ml Amp (Fig. 4*C*). The results of anti-Bla IB (Fig. 4*C*) showed that the relative VemP-Bla levels in the Amp-resistant strains (lanes 3 and 6) were comparable to or higher than the control (lane 2), whereas the levels in the Amp-sensitive strain (lane 5) were significantly lower than those in the control. Thus, this reporter system allowed a rough estimation of the arrest stability of VemP mutants from the growth phenotype.

Next, the QNQN and LLLLF mutations described in Figure 3 were introduced into the reporter and analyzed similarly (Fig. 4, *D* and *E*). As expected, the Δ*ppiD* strain carrying p-*vemP(QNQN)-bla* (open diamonds) grew well in the Amp-containing medium (Fig. 4*D*), whereas the Δ*ppiD* strain carrying p-*vemP(LLLLF)-bla* (open squares) did not. The WT strain carrying the latter plasmid (closed squares) showed slightly slower growth than that observed with the combination of WT host and reporter (closed triangles). Consistent results were obtained for both growth on Amp plates and cellular accumulation levels of the VemP-Bla derivatives (Fig. 4*E*). Taken together, we conclude that this reporter system is useful for isolating *cis* mutations that alter the stability of the VemP translation arrest.

### Isolation and characterization of VemP arrest mutants by random mutagenesis targeting the SHS

**Figure 5:**
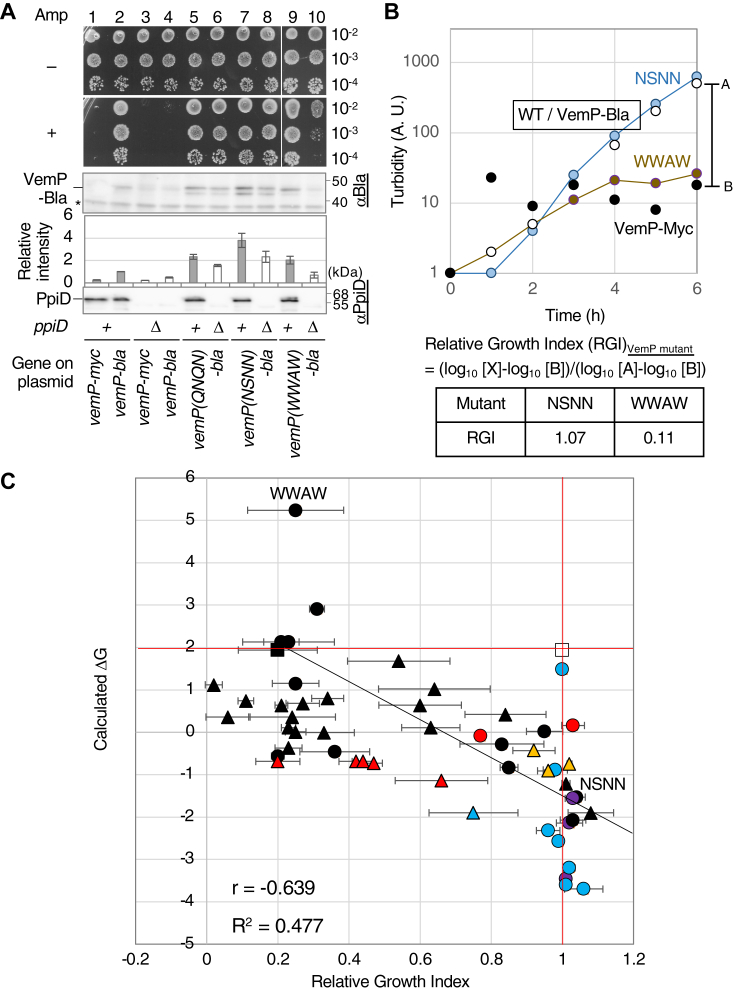
**Characterization of the SHS random mutants of VemP.***A*, *upper panels*: growth of Δ*ppiD* strain expressing VemP-Bla derivatives on L-plate. Cells carrying the plasmids indicated below were grown as shown in Figure 4*C* and diluted with saline as indicated, spotted on the L-plate supplemented with 1 mM IPTG and 20 μg/ml Cm without (−) or with (+) 10 μg/ml Amp, and incubated at 30 ˚C for 16 h. The result shown is a representative of two biological replicates. *Lower panels:* cellular accumulation of the VemP-Bla mutants. The experiments were performed as shown in Figure 4*C*. ∗ indicates a protein nonspecifically cross-reacting with anti-Bla antibody. The result shown is a representative of two biological replicates. The VemP-Bla bands were quantified, normalized, and plotted as a bar graph, as shown in Figure 4*C*. *B*, growth curves of strains expressing the VemP derivatives in the liquid culture. Experiments with the indicated cells were performed as in Figure 4*B*. A and B in the graph indicate the turbidity of WT cells expressing VemP-Bla and Δ*ppiD* cells expressing VemP-Myc at 6 h cultivation, respectively. The result shown is a representative of two biological replicates. *C*, relationship between the hydrophobicity of the target region and the relative growth index (RGI) of cells expressing the mutants. The RGI of all isolated VemP-Bla derivatives was calculated using the formula shown in Figure 5*B*. Furthermore, using the Wimley and White hydrophobic scale, ΔG_membrane to water_ values for five residues (78–82) in the SHS of all VemP-Bla derivatives were calculated. The ΔG values and the RGIs with SD (N = 2) of all the VemP-Bla derivatives were plotted on the *vertical and horizontal axes*, respectively. The *open square*, whose x and y values are shown as *red lines*, and the *closed square* indicate the WT cells expressing WT VemP-Bla and the Δ*ppiD* cells expressing WT VemP-Bla, respectively. The results with the mutants obtained from groups 1 and 2 are shown as circles and triangles, respectively. The positions corresponding to the WWAW and NSNN mutants are indicated with their names. *Colored marks* represent the Δ*ppiD* cells expressing VemP-Bla mutants containing Arg (*red*), Lys (*yellow*), negatively charged residues (*pale blue*), and both positively and negatively charged residues (*purple*) in their SHS. The regression line obtained by a least squares method using all data except the *open square* is shown with r and R^2^ values. SHS, short hydrophobic segment.

To understand the role of the SHS (L^78^ALF) in the TTAC, we isolated mutants with altered arrest stability by random mutagenesis of the SHS using the VemP-Bla reporter (see Experimental procedures for details). We constructed two types of plasmid libraries; in one (group 1), all four residues were targeted, whereas in the other (group 2), only the two C-terminal residues were targeted. The plasmid libraries were introduced into the Δ*ppiD* strain, and the transformants obtained were screened for their growth phenotypes on the L-plate with or without 10 μg/ml Amp. Finally, we isolated 15 Amp^R^ and 8 Amp^S^ mutants from group 1 and 11 Amp^R^ and 16 Amp^S^ mutants from group 2. We then monitored the growth of Δ*ppiD* strains expressing the isolated VemP-Bla mutants both in a liquid medium and on a plate and also determined the accumulation levels of the VemP-Bla mutants. These results are summarized in Tables 1 for group 1 and 2 for group 2. Hereafter, a single-letter representation of the SHS amino acid sequence is used as the mutant name. Figure 5, *A* and *B* show the results obtained with the NSNN and WWAW mutants (used as representatives of the Amp^R^ and Amp^S^ mutants, respectively). The Δ*ppiD* strain expressing the NSNN (Fig. 5*A*, lane 8) or WWAW (lane 10) mutant grew well or poorly, respectively, on L-Amp plates. In the L-Amp liquid medium, the strain-expressing NNSN grew well, but the strain-expressing WWAW did not (Fig. 5*B*, pale blue and brown). Consistent with their growth phenotypes, the accumulation level of the NSNN and WWAW mutants was twice and less than that of the positive control (lane 2), respectively (Fig. 5*A*). Similar results were obtained for most of the other Amp^R^ and Amp^S^ mutants (see Tables 1, 2, Fig. S8). To semiquantitatively assess the growth ability of the Δ*ppiD* strain expressing each VemP-Bla mutant in the L-Amp medium, we calculated the relative growth index (RGI) of each strain from its turbidity after 6 h of cultivation according to the formula listed in Figure 5*B*. The RGI values roughly represent the relative extent of translation arrest of each mutant in the Δ*ppiD* strain. The values of the WT strain expressing the WT VemP-Bla (positive control) and the Δ*ppiD* strain–expressing VemP-Myc (negative control) were set to 1 and 0, respectively. Thus, RGI values greater than 1 and negative RGI values indicate better growth than the positive control and lower growth than the negative control, respectively. To examine the relationship between the hydrophobicity of the target region of the mutants and their translation arrest ability, the ΔG _bilayer to water_ values of five residues in the 78 to 82 region of mutants were calculated using the experimentally determined Wimley and White scale as an index of hydrophobicity (30) and plotted against the RGI values (Fig. 5*C*). As expected, the variation of ΔG values calculated from the group 2 mutants (triangles) was in the range of −2 to 2 Kcal/mol, which is smaller than that of ΔG values from the group 1 mutants (ΔG values were −4 to 5 Kcal/mol) (circles).

**Table 1:** Summary of the *in vivo* analyses of the VemP-Bla derivatives obtained by random mutagenesis (group 1)

Sequence^a^	ΔG _bilayer to water_^b^ (kcal/mol)	Screening^c^	RGI^d^	Growth on plate^e^	Accumulation^f^
LALF (in WT)	1.94	R	1	A	1
LALF	1.94	S	0.20 (±0.11)	–	0.31 (±0.03)
QNQN	−2.14	NE	1.02 (±0.04)	A	1.55 (±0.07)
NSNN	−1.53	R	1.04 (±0.02)	A	2.34 (±0.50)
WWAW	5.24	S	0.25 (±0.13)	C	0.69 (±0.26)
RGGF	0.16	R	1.03 (±0.03)	A	1.65 (±0.35)
VRYG	−0.09	R	0.77 (±0.01)	B	1.11 (±0.29)
EATD	−3.7	R	1.06 (±0.05)	A	1.60 (±0.13)
FDWS	1.48	R	1.0 (±0)	B	1.41 (±0.13)
QLET	−2.32	R	0.96 (±0.03)	A	1.81 (±0.20)
LDCK	−1.56	R	1.03 (±0.02)	A	1.05 (±0.21)
RNVE	−3.46	R	1.01 (±0.01)	A	1.36 (±0.09)
PNIS	−0.83	R	0.85 (±0.02)	B	1.09 (±0.10)
RSDC	−2.07	R	1.03 (±0.04)	A	1.82 (±0.23)
LSTS	0.02	R	0.95 (±0.06)	A^–^	1.12 (±0.08)
PNEA	−3.2	R	1.02 (±0.02)	A	1.21 (±0.02)
TSTE	−2.57	R	0.99 (±0)	A	1.28 (±0.09)
PCED	−3.6	R	1.01 (±0.01)	A	1.10 (±0.26)
PGYD	−0.89	R	0.98 (±0.03)	A^–^	0.91 (±0.04)
VWVL	2.13	S	0.23 (±0.13)	C^–^	0.38 (±0.09)
LTT–	−0.28	S	0.83 (±0.11)	A^–^	0.44 (±0.03)
ATIN	−0.56	S	0.2 (±0.02)	–	0.43 (±0.15)
CWFA	2.91	S	0.31 (±0.02)	B	0.38 (±0.13)
VWLV	2.13	S	0.21 (±0.05)	–	0.17 (±0.17)
TILL	1.15	S	0.25 (±0.07)	C^–^	0.12 (±0.11)
IVTN	−0.46	S	0.36 (±0.10)	B	0.1 (±0.01)

Amp, ampicillin.

a Amino acid sequence of the 78 to 81 region of VemP-Bla derivatives.

b Gibbs energy (from bilayer to water) of the residues in the 78 to 82 region of VemP-Bla calculated by using Wimley and White scale (1996) (30).

c Results of the initial screening of Δ*ppiD* cells expressing VemP-Bla derivatives on L-Amp plate. R and S indicate Amp-resistant and Amp-sensitive, respectively. NE indicates “not examined.”

d Relative growth index (RGI) of Δ*ppiD* cells expressing VemP-Bla derivatives were calculated according to the formula shown in Figure 5*B*. Mean values are shown with SD (N = 2, biological replicates).

e Growth phenotype of Δ*ppiD* cells expressing VemP-Bla derivatives on L-plate containing Amp (10 μg/ml). A, B, and C represent that Δ*ppiD* cells expressing VemP-Bla derivatives grew well for 10^4^-, 10^3^-, and 10^2^-fold diluted culture spots on the L-Amp plate, respectively. A^–^, B^–^, and C^–^ indicate that the cells grew poorly at each dilution condition. – indicates that cells did not grow under any condition.

f Cellular accumulation levels of VemP-Bla derivatives were measured as described to the legend in Figure 4*C*. Mean values are shown with SD. (N = 2, biological replicates).

**Table 2:** Summary of the *in vivo* analyses of the VemP-Bla derivatives obtained by random mutagenesis (group 2)

Sequence^a^	ΔG _bilayer to water_^b^ (kcal/mol)	Screening^c^	RGI^d^	Growth on plate^e^	Accumulation^f^
LF (in WT)	1.94	R	1	A	1
LF	1.94	S	0.20 (±0.11)	–	0.31 (±0.03)
YA	1.02	S	0.64 (±0.16)	B^–^	0.83 (±0.07)
KI	−0.43	R	0.92 (±0.06)	A^–^	0.74 (±0.11)
RS	−0.69	S	0.42 (±0.05)	C	0.64 (±0.23)
WN	1.68	R	0.54 (±0.12)	C	0.64 (±0.12)
LE	−1.21	R	1.01 (±0.01)	A	1.05 (±0.13)
HK	−0.91	R	0.96 (±0.03)	A	0.75 (±0.22)
HL	0.64	S	0.6 (±0.12)	B	0.56 (±0.11)
RQ	−1.14	S	0.66 (±0.15)	C	0.68 (±0.03)
HF	0.42	S	0.84 (±0.06)	B^–^	0.29 (±0.17)
GK	−0.75	R	1.02 (±0.01)	A	0.96 (±0.07)
SE	−1.9	R	1.08 (±0.09)	A^–^	1.03 (±0.23)
RS	−0.69	S	0.44 (±0.04)	C^–^	0.57 (±0.03)
RH	−0.73	S	0.47 (±0.03)	C^–^	0.64 (±0.01)
GS	0.11	R	0.63 (±0.08)	B^–^	0.77 (±0.10)
PH	−0.37	R	0.23 (±0.04)	C^–^	0.58 (±0.01)
SS	−0.01	R	0.33 (±0.09)	B^–^	0.59 (±0.02)
SR	−0.69	R	0.2 (±0.06)	C^–^	0.56 (±0.11)
ES	−1.9	R	0.75 (±0.12)	B	0.55 (±0.27)
LP^g^	0.36	S	0.24 (±0.12)	C	0.27 (±0.12)
LS	0.68	S	0.27 (±0.05)	B^–^	0.24 (±0.02)
PI	0.11	S	0.23 (±0.02)	B	0.30 (±0.01)
LI	1.12	S	0.02 (±0.02)	C	0.31 (±0.03)
AL	0.64	S	0.21 (±0.02)	B^–^	0.33 (±0)
LG	0.8	S	0.34 (±0.04)	A^–^	0.55 (±0.02)
LV	0.74	S	0.11 (±0.02)	B^–^	0.40 (±0.07)
VA	0.01	S	0.25 (±0.03)	B	0.39 (±0.02)
LP^g^	0.36	S	0.06 (±0.06)	B^–^	0.45 (±0.16)

a Amino acid residues at positions 80 and 81 of VemP-Bla derivatives.

b–f Described as ^b–f^ in Table 1.

g Synonymous mutations.

The RGI values of the group 2 mutants were widely scattered from 0 to 1, whereas those of the group 1 mutants were clustered around 0.2 and 1 (see Supporting discussion 2). The calculated ΔG *versus* RGI plot for the 50 isolated mutants suggests a significant negative correlation between the hydrophobicity of VemP SHS and the growth of cells expressing the VemP-Bla in the Amp-containing medium (r = −0.639, R^2^ = 0.477). We also found a significant positive correlation between the RGI values of VemP-Bla mutants and their cellular accumulation (r = 0.78, R^2^ = 0.68) (Fig. S9*A*, Supporting result 5). As an amino acid substitution would alter not only hydrophobicity but also other physical properties, including side chain volume and α-helix propensity at the mutated site, we also examined the relationships between RGI and the aforementioned two properties and found weaker correlations of RGI with the side chain volume (r = −0.32, R^2^ = 0.10) and the α-helix propensity of the SHS (r = 0.19, R^2^ = 0.04), suggesting that the hydrophobicity of the SHS would be a major factor in determining the RGI values (Fig. S10, *A* and *B*, Supporting discussion 3). These results suggest a positive correlation between the degree of hydrophobicity of the SHS and the stability of the VemP translation arrest.

### Systematic mutational analyses of the SHS

#### Effect of the SHS hydrophobicity on RGI

**Figure 6:**
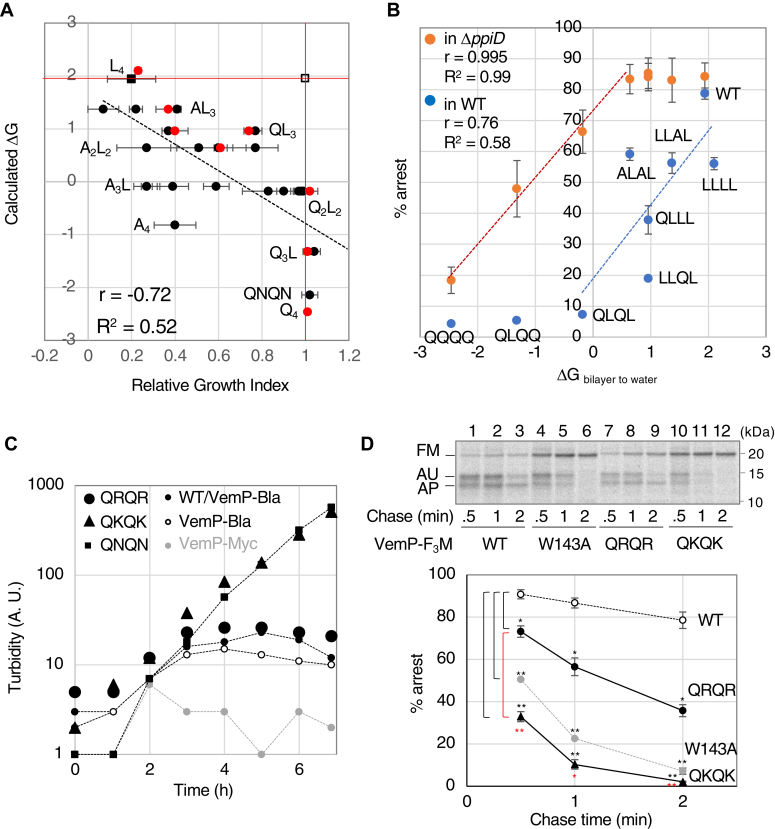
**Systematic mutational analysis of SHS in VemP.***A*, a total of 31 VemP-Bla derivatives, whose SHS consists of different combinations of Gln, Ala, and Leu, were systematically constructed and expressed in Δ*ppiD* cells. Cells expressing the VemP-Bla derivatives were cultured, as indicated in Figure 4*B*. The RGIs with SD (N = 2, biological replicates) of cells expressing the VemP-Bla derivatives and the ΔG values for five residues (78–82) of the corresponding mutants were calculated and plotted, as shown in Figure 5*C*. The regression line obtained by a least squares method using all data except the *open square* is also shown. Eight Mutants colored in *red* represent those used for pulse-chase experiments in Figure 6*B*. *B*, pulse-chase experiments with both Δ*ppiD* (*orange*) and WT (*blue*) cells expressing VemP-F_3_M mutants. Either Δ*ppiD* or WT cells carrying p-*vemP-f*_*3*_*m* with the indicated mutation were grown, induced, subjected to pulse-chase experiments, and analyzed as described in Figure 1. Raw data and quantified results are shown in Figure S11. Means of the percentages of the arrested form of VemP recorded at 1 min-chase in the Δ*ppiD* cells (*orange*) and at 30 s-chase in the WT cells (*blue*) were plotted against the calculated ΔG values of the mutants (error bars are SD (N = 2)). The regression lines in *orange and blue* obtained by a least squares method using data of the four mutants from the smallest ΔG value and of the six mutants from the largest ΔG value plus WT, respectively, are also shown with r and R^2^ values. *C*, growth curve of Δ*ppiD* strain expressing a VemP (either QNQN, QKQK, or QRQR)-Bla derivative. Δ*ppiD* cells expressing the indicated VemP-Bla derivatives and WT cells expressing VemP-Bla were cultured as shown in Figure 3*B*, except that the medium was supplemented with 25 μg/ml Amp. The result shown is a representative of two biological replicates. *D*, pulse-chase experiments of cells expressing VemP-F_3_M derivatives. Δ*ppiD* cells carrying a plasmid encoding the indicated VemP-F_3_M derivative were grown, induced, subjected to pulse-chase experiments, and analyzed, as shown in Figure 1. The result shown is a representative of two biological replicates. Means with SD (N = 2) are plotted against the chase time. An unpaired two-tailed Student's *t* test was used to statistically compare the values between the groups. ∗*p* < 0.05 and ∗∗*p* < 0.01. Amp, ampicillin; RGI, relative growth index; SHS, short hydrophobic segment.

To systematically investigate the inverse correlation between the hydrophobicity of the SHS and RGI under more simplified conditions, we constructed 31 VemP-Bla mutants by replacing all four residues in the SHS with three types of uncharged amino acids of different hydrophobicity—Leu (ΔG _bilayer to water_ = 0.56 kcal/mol), Ala (−0.17 kcal/mol), and Gln (−0.58 kcal/mol)—and examined the growth of the Δ*ppiD* strain expressing these VemP-Bla mutants in the L-Amp medium (Fig. 6*A* and Table 3) and the cellular accumulation of the mutant proteins (Fig. S9*B*), as shown in Figure 5*B* and Figure S9*A*, respectively. Although the cells expressing some mutants with the same amino acid composition but a different sequence in the SHS showed significantly different RGI values, overall the RGI values tended to increase gradually as the hydrophobicity of the SHS decreased (r = −0.72, R^2^ = 0.52). A correlation between the RGI values and the cellular accumulation of the VemP-Bla derivatives was also observed (r = 0.84, R^2^ = 0.71) (Fig. S10*B*). These results further highlight the importance of hydrophobicity in determining RGI. In addition, we selected eight mutants colored in red in Figure 6*A* that possessed different RGIs and different hydrophobicity in the SHS, constructed VemP-F_3_M derivatives with these mutations, and directly measured their TTAC kinetics in both WT and the Δ*ppiD* cells by pulse-chase experiments (Fig. S11). A chase time-dependent conversion of these VemP derivatives from the arrested to the full-length form was observed, only when the VemP derivatives with a relatively hydrophobic or hydrophilic SHS region were expressed in the WT or Δ*ppiD* strain, respectively. The results showed a significant positive correlation between the stability of their translation arrest and the hydrophobicity of their SHS in both strains (Fig. 6*B*), strengthening the idea that the hydrophobicity of the SHS determines the stability of the VemP arrest.

**Table 3:** Summary of the *in vivo* analyses of the VemP-Bla derivatives constructed by systematic mutagenesis

Sequence^a^	ΔG _bilayer to water_^b^ (kcal/mol)	RGI^c^	Accumulation^d^
LALF (in WT)	1.94	1	1
LALF	1.94	0.20 (±0.11)	0.31 (±0.03)
QNQN	2.14	1.02 (±0.04)	1.55 (±0.07)
LLLL	2.1	0.23 (±0.01)	0.37 (±0.14)
ALLL	1.37	0.22 (±0.03)	0.26 (±0.03)
LALL	1.37	0.07 (±0.07)	0.29 (±0.02)
LLAL	1.37	0.37 (±0.01)	0.30 (±0.06)
LLLA	1.37	0.41 (±0.01)	0.34 (±0.02)
LALA	0.64	0.61 (±0.12)	0.37 (±0.04)
LAAL	0.64	0.51 (±0.13)	0.32 (±0.05)
ALLA	0.64	0.27 (±0.14)	0.24 (±0.02)
AALL	0.64	0.27 (±0.14)	0.23 (±0.04)
ALAL	0.64	0.60 (±0.00)	0.37 (±0.07)
LLAA	0.64	0.77 (±0.10)	0.30 (±0.10)
LAAA	−0.09	0.59 (±0.06)	0.32 (±0.12)
ALAA	−0.09	0.39 (±0.07)	0.23 (±0.06)
AALA	−0.09	0.27 (±0.03)	0.40 (±0.19)
AAAL	−0.09	0.27 (±0.06)	0.32 (±0.22)
AAAA	−0.82	0.40 (±0.10)	0.18 (±0.04)
QLLL	0.96	0.40 (±0.06)	0.33 (±0.14)
LQLL	0.96	0.77 (±0.03)	0.48 (±0.05)
LLQL	0.96	0.74 (±0.02)	0.33 (±0.09)
LLLQ	0.96	0.37 (±0.01)	0.17 (±0.07)
LQLQ	−0.18	0.97 (±0.03)	0.78 (±0.11)
LQQL	−0.18	0.98 (±0.03)	1.06 (±0.06)
QLLQ	−0.18	0.90 (±0.03)	0.73 (±0.22)
QQLL	−0.18	0.99 (±0.01)	0.77 (±0.15)
QLQL	−0.18	1.02 (±0.04)	0.80 (±0.18)
LLQQ	−0.18	0.83 (±0.12)	0.40 (±0.01)
LQQQ	−1.32	1.01 (±0.01)	0.90 (±0.11)
QLQQ	−1.32	1.01 (±0.02)	1.17 (±0.33)
QQLQ	−1.32	1.04 (±0.03)	0.91 (±0.23)
QQQL	−1.32	1.04 (±0.03)	0.94 (±0.06)
QQQQ	−2.48	1.01 (±0.01)	1.17 (±0.34)

Abbreviation: RGI, relative growth index.

a,b Described in Table 1.

c Described as ^d^ in Table 1.

d Described as ^f^ in Table 1.

#### Effect of positively charged residues introduced into the SHS on RGI

The Lys-containing mutants (yellow) and Arg-containing mutants (red) in group 2 (triangles) showed a clear difference in the RGI values (Fig. 5*C*); however, they may possess similar ΔG values. To elucidate the effects of these positively charged residues on the VemP arrest cancellation, we constructed the VemP-Bla derivatives in which the SHS was replaced by the QKQK or QRQR sequences and examined the growth of the Δ*ppiD* strains expressing them. Since the cells expressing either of these mutants as well as the QNQN mutant grew well in the medium containing 10 μg/ml Amp (see Table 4), we used L-medium containing 25 μg/ml Amp (Fig. 6*C*, Table 4) to detect any difference in their Amp resistance. The strain expressing either WT VemP-Bla (small closed circles) or the QRQR mutant (closed circles) did not grow in this medium, whereas the strain expressing the QKQK (closed triangles) or QNQN (small closed squares) mutants grew well, suggesting that Arg residues in the SHS stabilize the VemP translation arrest. We confirmed this by measuring the kinetics of the TTAC of VemP-F_3_M derivatives with QRQR and QKQK in the Δ*ppiD* strain (Fig. 6*D*). The arrested form of QKQK was rapidly converted to full-length, as was the arrest-defective W143A mutant. In contrast, the TTAC of QRQR was significantly slower than that of QKQK, despite the lower calculated ΔG for QRQR (−2.92 kcal/mol) than for QNQN (−2.14 kcal/mol). Therefore, Arg residues in the SHS appear to exert an arrest-stabilizing effect in contrast to other hydrophilic residues, including Lys.

**Table 4:** Summary of the growth phenotype of the VemP-Bla derivatives with positively charged residues in the SHS in L-medium containing different concentrations of Amp

Sequence^a^	ΔG _bilayer to__water_^b^ (kcal/mol)	RGI^c^ (in 10 μg/ml Amp)	RGI^d^ (in 25 μg/ml Amp)
QQQQ (in WT)	−2.46	NE	1
LALF (in WT)	1.94	1	0.40 (±0.00)
LALF	1.94	0.20 (±0.18)	0.28 (±0.00)
QNQN	−2.14	1.01 (±0.0)	0.96 (±0.00)
QRQR	−2.92	0.89 (±0.07)	0.44 (±0.00)
QKQK	−3.28	1.02 (±0.02)	0.88 (±0.09)

Amp, ampicillin; RGI, relative growth index; SHS, short hydrophobic segment.

a,b Describbed as ^a, b^ in Table 1.

c Δ*ppiD* cells expressing VemP-Bla derivatives as indicated were grown in L-1 mM IPTG medium containing 10 μg/ml Amp. The mean values of RGIs of these cultures to that of WT cells expressing WT VemP-Bla are shown with SD (N = 2, biological replicates). NE indicates “not examined.”

d Similar growth experiments using these cells indicated were performed except that concentration of Amp was 25 μg/ml. RGIs of the cells expressing the indicated VemP-Bla derivatives were calculated using WT cells expressing VemP(QQQQ)-Bla, instead of WT cells expressing VemP-Bla, as a positive reference, because the latter cells hardly grew in this condition. Mean values are shown with SD (N = 2, biological replicates).

### Coordination of the SHS and Arg-85 in VemP translation arrest

**Figure 7:**
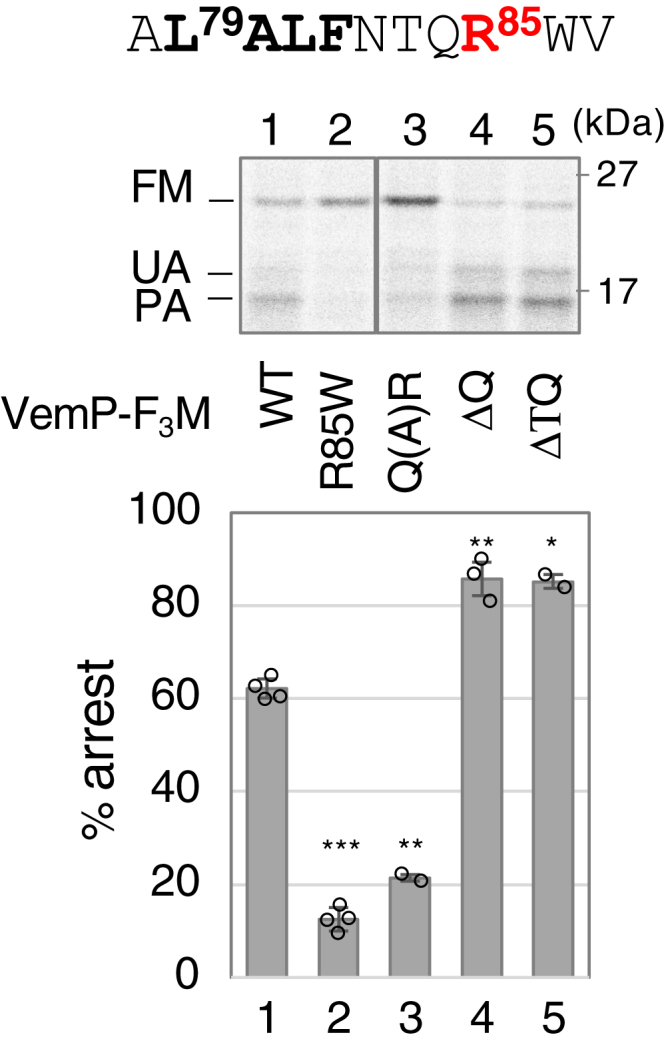
**Importance of the distance between SHS and Arg-85.** WT cells carrying plasmids encoding the indicated VemP-F_3_M derivatives were grown, induced, pulse-labeled for 30 s, chased for 1 min, and analyzed as shown in Figure 1. The result shown is a representative of at least two biological replicates. The mean values with SD. (N ≥ 2) and the raw data are shown in the *lower graph*. An unpaired two-tailed Student's *t* test was used to statistically compare the values between the WT and each mutant. ∗*p* < 0.05, ∗∗*p* < 0.01, and ∗∗∗*p* < 0.001. SHS, short hydrophobic segment.

The apparent similarity of the phenotypes of the SHS (Fig. 6, *B* and *C*) and Arg-85 (25) mutants, as well as their close positioning (they are only three residues apart, with this distance being fully conserved among VemP orthologs; Fig. S6*A*) led us to examine the possibility that these elements cooperate in regulating the TTAC. We constructed three VemP-F_3_M derivatives: Q(A)R mutant, in which Ala was inserted between Gln-84 and Arg-85 to increase the distance and ΔQ84 and Δ(T83Q84) mutants, in which one and two residues between these elements were deleted, respectively, to decrease the distance (Fig. 7). To assess the TTAC of these mutants in the WT strain, the percentage of the arrested species in the total VemP derivatives at the 30-s chase point is calculated. The Ala insertion destabilized the arrested state to the same extent as the R85W mutation, whereas both deletions slightly but significantly stabilized it. Since the Cys substitutions of Thr-83 and Gln-84 did not affect VemP-PhoA activity (Fig. S4*A*), the observed stabilizing effects were not attributed to the absence of these residues but rather to the change in distance. These results suggest that proper positioning is important for the full functionality of these elements in the TTAC.

### Identification of the (Hphi)xP motif as a novel *cis* element involved in the stability of VemP elongation arrest

**Figure 8:**
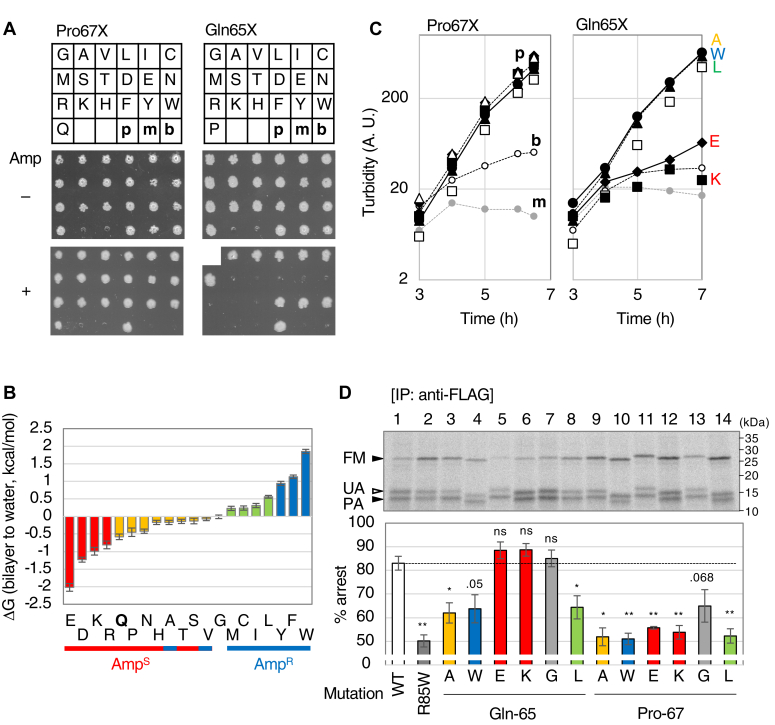
**Mutational analyses of Gln-65 and Pro-67 in VemP.***A*, growth phenotype of Δ*ppiD* cells expressing VemP-Bla derivatives with systematically mutated Gln-65 (*right*) or Pro-67 (*left*) residues. Δ*ppiD* cells carrying a plasmid encoding the VemP-Bla derivative indicated in the *upper panels* were grown, diluted, spotted on L-Cm-1 mM IPTG with (+) or without (−) 10 μg/ml Amp, and incubated at 30 ˚C for 16 h as in Figure 4*C*. p, m, and b represent WT cells/p-*vemP-bla* (positive control), Δ*ppiD* cells/p-*vemP-myc* (*m*yc negative control), and Δ*ppiD* cells/p-*vemP-bla* (*b*la negative control), respectively. *B*, relationship between the growth phenotypes of the Δ*ppiD* cells expressing one of the 20 kinds of VemP(Q65X)-Bla mutants and the experimentally determined ΔG_bilayer to water_ values of 20 amino acid residues (30). Amino acids are ordered according to their ΔG_bilayer to water_ values from the smallest on the *left* to the largest on the *right*. Charged (*red*), hydrophilic (*yellow*), hydrophobic (*pale green*), and aromatic (*blue*) residues are colored. Based on the results in Figure 8*A*, the growth phenotypes of cells expressing VemP(Q65X)-Bla derivatives on an L-Amp plate are presented as *red* (Amp^S^) and *blue* (Amp^R^) *horizontal bars* at the *bottom of the graph*. *C*, representative growth curves of Δ*ppiD* cells expressing VemP-Bla derivatives with a mutation at positions 67 (*left*) and 65 (*right*). Δ*ppiD* cells carrying the indicated p-*vemP-bla* derivatives were grown, as shown in Figure 4*B*. *Small closed circles, small open circles, and small gray circles* with *dashed lines* represent growth curves of WT cells carrying p-*vemP-bla* (positive control), Δ*ppiD* cells carrying p-*vemP-bla* (negative control), and Δ*ppiD* cells carrying p-*vemP-myc* (negative control), respectively. The result shown is a representative of two biological replicates. *D*, pulse-chase experiments of Δ*ppiD* cells expressing VemP::FLAG-LacZα derivatives. Δ*ppiD* cells carrying the indicated p-*vemP::flag-lacZα* derivatives were grown, induced, pulse-labeled for 30 s, chased for 1 min, and analyzed as shown in Figure 1. However, IP was performed using the anti-FLAG M2 beads, because mutations at positions 65 and 67, located in the epitope region (59–73) for anti-VemP antibodies, greatly reduced the IP efficiency of labeled VemP derivatives. The result shown is a representative of two biological replicates. Means with SD (N = 2) are shown at the *bottom of the gel*. An unpaired one-tailed Student's *t* test was used to statistically compare the values between the WT and each mutant. ∗*p* < 0.05, ∗∗*p* < 0.01, ∗∗∗*p* < 0.001, and *ns*, not significant. *p* values are shown for the mutants with those in the range 0.05 to 0.1. Amp, ampicillin; Cm, chloramphenicol; IP, immunoprecipitation.

Cys (Fig. S4*A*) and Ala (Fig. S12*A*) substitution analyses of the Q^65^SP sequence revealed that the substitutions at positions 65 and 67 destabilized the VemP arrested state, whereas substitutions at position 66 did not (Fig. S12, *A* and *B*, Supporting result 6). Since a His_10_-VemP(ΔSS) (P67A)-F_3_M derivative stably accumulated in the arrested form *in vivo*, as did the R85W and QNQN mutants (Fig. S7), we expected Gln-65 and Pro-67 to also act as important *cis* elements in the proper TTAC. We then performed extensive mutational analyses targeting these two residues. Nineteen different p-*vemP(Q65X or P67X)-bla* plasmids encoding VemP-Bla with one of the 19 amino acid residues other than the original at either Gln-65 or Pro-67 were constructed and introduced into the Δ*ppiD* strain, and the growth of the strains was examined. All 19 strains expressing the P67 mutants grew on the L-Amp plate (Fig. 8*A*, lower left). For Gln-65, strains expressing the mutants with a hydrophobic residue were Amp-resistant but those with a hydrophilic residue were Amp-sensitive (Fig. 8*A*, lower right, and Fig. 8*B*). The growth of the Δ*ppiD* strain–expressing VemP(Q65G)-Bla differed among the four transformants examined (Fig. S12*C*, Supporting result 7). Essentially the same results were obtained when growth in liquid Amp medium was examined for six VemP-Bla derivatives carrying a residue with a different chemical property (Ala, Trp, Glu, Lys, Gly, or Leu) at either position 65 or 67 (Figs. 8*C* and S12*C*). In addition, we used 12 kinds of VemP::FLAG-LacZα derivatives shown in Figure 8*D* to evaluate their TTAC kinetics in the Δ*ppiD* strain (Fig. 8*D*). All Pro67 mutations destabilized the arrested state, whereas the hydrophilic (Glu, Lys, or Gly) and hydrophobic (Trp, Leu, or Ala) residues at position 65 stabilized and destabilized the arrested state, respectively. Taken together, we concluded that Pro-67 and Gln-65 are new *cis* elements involved in the regulation of the VemP TTAC; the former plays an essential role in the proper TTAC, whereas the hydrophilic nature of the latter is also significant for its regulation. According to the requirement of these residues, we refer to the element as the (Hphi)xP motif. (Hphi and x represent hydrophilic and any amino acid residues, respectively).

### Effects of *cis* elements on the stabilization of the VemP arrest state in *trans* factor mutant strains

**Figure 9:**
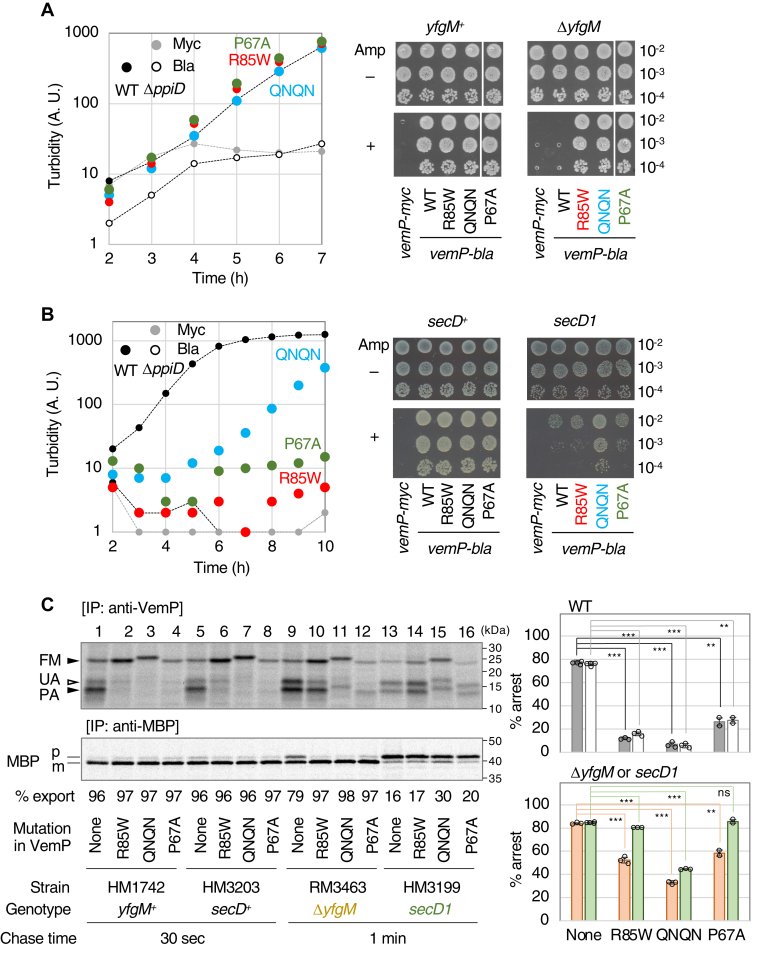
**Effects of *cis*-element mutations on VemP translation arrest in *trans*-factor mutant strains.** Growth of Δ*yfgM* (*A*) or *secD1* (*B*), a mutant allele of *secD* that reduces the cellular accumulation level of SecD/F to undetectable levels, cells expressing VemP-Bla derivatives in the liquid medium (*left*) and on a plate (*right*). *Left:* Δ*yfgM* or *secD1* cells carrying p-*vemP(R85W (red), QNQN (blue)* or *P67A (green))-bla* were grown in the L-medium containing 10 μg/ml Amp, as shown in Figure 4*B*. HM1742 and HM3203 are isogenic "WT" strains of the Δ*yfgM*, and the *secD1* mutants, respectively. Growth curves of the WT cells expressing VemP-Bla (*small black*), either Δ*yfgM* or *secD1* cells-expressing VemP-Bla (*small open black*) or VemP-Myc (*small gray*), are also shown as controls. The result shown is a representative of two biological replicates. *Right:* WT, Δ*yfgM*, or *secD1* cells carrying either p-*vemP-myc* or p-*vemP* (WT*, R85W (red), QNQN (blue), or P67A (green))-bla* were grown, diluted, spotted on an L-plate without (−) and with (+) 10 μg/ml Amp, as shown in Figure 5*A*, and incubated at 30 ˚C for 16 h. The result shown is a representative of two biological replicates. *C*, pulse-chase experiments of *trans*-factor mutant cells expressing VemP-F_3_M derivatives. WT (lanes 1–8), Δ*yfgM* (lanes 9–12), or *secD1* (lanes 13–16) cells carrying p-*vemP-f*_*3*_*m* with the indicated mutation were grown, induced, pulse-labeled for 30 s, chased for 30 s (lanes 1–8) or 1 min (lanes 9–16), and subjected to IP with anti-VemP antibodies (*upper gel*) and anti-MBP antibodies (*lower gel*). The samples were analyzed as shown in Figure 1. The result shown is a representative of at least two biological replicates. The mean values of the percentage of the arrest of VemP derivatives are shown with SD (N ≥ 2) and the raw data in the *upper right* (WT) and *lower right* (*trans* mutants) *graphs*. The *gray and white bars* in the *upper graph* represent the results obtained by using HM1742 and HM3203 as host strains, respectively. The *orange and green bars* in the *lower graph* show those obtained by using HM3463 (Δ*yfgM*) and HM3199 (*secD1*), respectively. An unpaired two-tailed Student’s *t* test was used to statistically compare the values between the groups. ∗∗*p* < 0.01 and ∗∗∗*p* < 0.001. The mean values of the percentage of the MBP export are shown under the *lower gel*. Amp, ampicillin; IP, immunoprecipitation; MBP, maltose binding protein.

Mutational analyses of VemP in the Δ*ppiD* strain led to the identification of Arg-85, the SHS, Gln-65, and Pro-67 as *cis* elements involved in its proper TTAC. We next investigated the effects of *cis*-element mutations on the VemP TTAC in the Δ*yfgM* and *secD1* strains, which lack other *trans* factors, because the VemP arrested state was stabilized in both mutant strains ((25, 27), Fig. 1*D*) as well as the Δ*ppiD* strain ((25), Fig. 1*C*). Δ*yfgM* strains expressing the VemP-Bla derivative with either the R85W, QNQN, or P67A mutation were all viable in both liquid (Fig. 9*A*, left graphs) and solid Amp-containing media (Fig. 9*A*, right photos), similar to the results obtained with the Δ*ppiD* mutant (Figs. 4, and 8). Interestingly, in contrast to the Δ*ppiD* and Δ*yfgM* mutants, the *secD1* strain expressing the VemP-Bla derivative with either R85W (red) or P67A (green) failed to grow in both growth conditions, whereas the same strain expressing the QNQN mutant grew slower but significantly (blue).

We also evaluated the efficiency of the TTAC of VemP-F_3_M with these *cis* mutations in the WT, Δ*yfgM*, and *secD1* strains. Since the VemP arrest in the Δ*yfgM* strain, as in the *secD1* strain, was expected to be released more slowly than in the WT strain (Fig. 1, *A* and *B*), we analyzed the arrested state of VemP at 30 s and 1 min chase points in the WT and the mutant strains, respectively. In the case of the WT strains, the introduction of any of the three *cis* mutations significantly reduced the levels of the arrested form of VemP (Fig. 9*C* right upper graph), suggesting that these mutations enhance the VemP TTAC. Consistent with the results in Figure 9*A*, the translation arrest of all VemP *cis* mutants was destabilized in the Δ*yfgM* mutant strain (Fig. 9*C*, lower graph, orange bars). The behavior of VemP in *secD1* mutants differed depending on the *cis* mutations carried (Fig. 9*C* lower graph, pale green bars). The ratio of the arrested forms was almost the same for the R85W and P67A mutants and WT VemP, whereas it was significantly lower for the QNQN mutant. Thus, the introduction of R85W or P67A does not alter the stability of the VemP arrested state in the *secD1* strain. These results suggest that SecD/F and YfgM/PpiD do not play the same role in the VemP TTAC, similar to that observed with *cis* elements.

## Discussion

VemP specifically monitors the SecD/F activity by arresting its translocation at a later stage, with release dependent on both SecD/F and PpiD/YfgM (24, 25, 27). Upon conducting a genetic and biochemical analysis to elucidate the molecular mechanism of the TTAC, we recorded the following findings: 1) SecY was involved in the VemP TTAC (Fig. 1) (see Supporting discussion 4). 2) In the arrested state, Arg-74 of the nascent VemP was stably held in the vicinity of TM7 near the SecY pore ring (Figs. 2). 3) The SHS (L^78^ALF) acted as a *cis* element to stabilize the VemP translation arrest with a positive correlation between its hydrophobicity and the stability of the arrested state (Figure 3, Figure 4, Figure 5, Figure 6). 4) The introduction of Arg, but not Lys, into the SHS led to the retardation of the TTAC (Fig. 6, *C* and *D*), similar to that observed with Arg-85 (25), suggesting the special role of Arg in the TTAC. 5) The distance between the SHS and Arg-85 was important for proper TTAC (Fig. 7). 6) The novel (Hphi)xP motif acted as a *cis* element to regulate the stability of translation arrest (Fig. 8), in which the hydrophilicity of Gln-65 was important. Furthermore, Pro-67 was essential to this process. 7) Mutational alterations in these three *cis* elements similarly destabilized the arrested state in both the Δ*ppiD* and Δ*yfgM* strains (Fig. 9), consistent with the previous finding that PpiD and YfgM heterodimers acted as a functional unit (27). These *cis* elements should play an important role in the normal TTAC process since their mutations destabilized the arrest even in the WT strain. In the *secD1* strain, the QNQN mutation of the SHS destabilized the arrest, whereas the R85W and P67A mutations did not (Fig. 9). These results suggest that the PpiD/YfgM and SecD/SecF complexes play different roles in the VemP TTAC and that the roles of these *cis* elements are not the same (see below).

### Predicted positions of the *cis* elements of the arrested nascent VemP polypeptide in the SecY/E/G translocon

**Figure 10:**
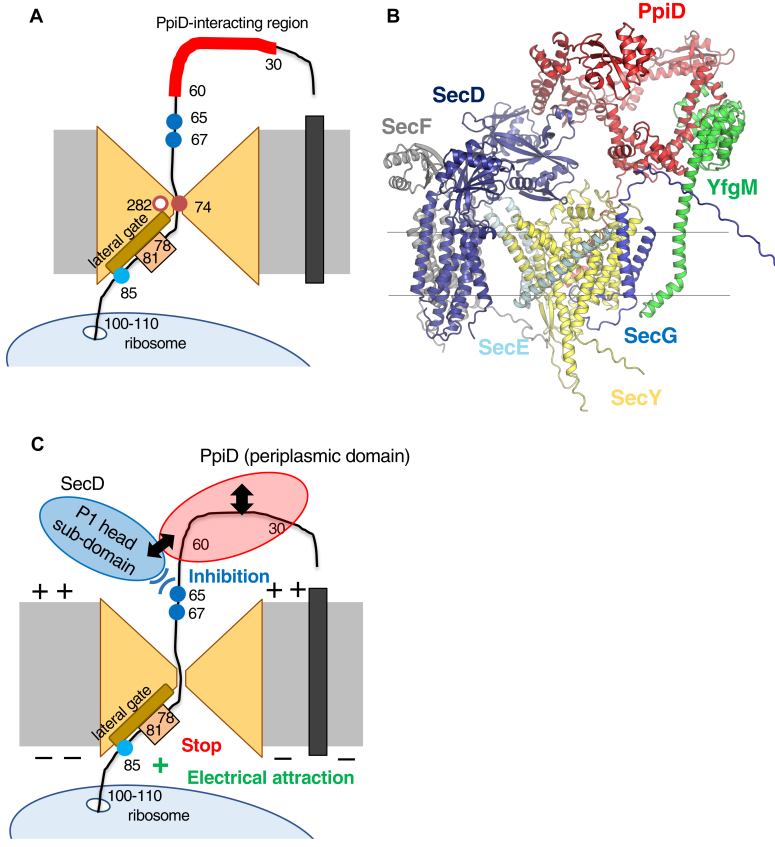
**Model for the *cis* element mediated arrest cancellation of VemP.***A*, a schematic representation of the nascent VemP polypeptide passing through SecY in the VemP translation arrested state. See text for details. Residues focused on in this study are indicated with residue numbers. *B*, a structural model of SecY/E/G-SecD/F-PpiD/YfgM supercomplex predicted by AlphaFold2. *C*, working hypothesis of the *cis* element mediated translation arrest cancellation of VemP. Only functionally important domains of Sec factors are shown. See text for details.

Based on this and previous studies, we constructed a model of the positioning of the *cis* elements in SecY in the arrested state of VemP (Fig. 10*A*). In the Δ*ppiD* strain, Arg-74 in arrested VemP was in close proximity to Ser-282 in SecY TM7 near the pore ring (Fig. 2, *B* and *F*), suggesting that the SHS was located near the lateral gate formed by TM2 and TM7 in the cytoplasmic funnel of the channel. In this case, Arg-85 would be located within the cytoplasmic funnel or at the cytoplasm-membrane interface. Gln-65 and Pro-67 in the (Hphi)XP motif would be located around/near the (unidentified) substrate-binding site in the periplasmic domain of PpiD in or near the periplasmic funnel of SecY. This finding is consistent with the AlphaFold2-predicted SecY/E/G-SecD/F-PpiD/YfgM supercomplex (Fig. 10*B*), in which the P1 head subdomain of SecD is located between the periplasmic SecY funnel and the periplasmic domain of PpiD. Based on the aforementioned model of *cis*-element positioning, the AF2-predicted structural model and the biochemical results of this study, we discuss the roles of *cis* elements in regulating the TTAC.

### Role of the SHS in the VemP arrest cancellation

Since the VemP nascent chain possesses tRNA at its C terminus, the translocation of arrested VemP cannot be completed unless the arrest is released. In this situation, a ribosome containing the VemP-tRNA would sterically hinder the translocation of nascent VemP, allowing the localization of SHS around the lateral gate of SecY and facilitating its interaction. Our finding that the half-life of the arrested state of VemP was more than 30 s in the WT strain (Figs. 1, 9*C* (24); indicates that nascent VemP remains in SecY/E/G for a substantial period. The inverse correlation between the rate of the translation arrest cancellation and the degree of the SHS hydrophobicity (r = −0.66) (Figs. 5*C* and 6*A*) suggests that the polypeptide translocation was halted by the hydrophobic interaction of the SHS with the lateral gate, which plays a critical role in determining the timing of TTAC.

Several lines of evidence suggest that an interaction between the lateral gate and a hydrophobic sequence in a substrate halts its translocation. 1) A medium hydrophobic sequence (8, 9 residues) in a model membrane protein was recognized by the lateral gate to halt its translocation and allow its integration into the membrane *in vitro*, when the energy supply for the reaction was limited to inhibit protein translocation (31, 32). 2) Movement of a substrate-polypeptide containing an S-S bond near its C terminus was stably halted at the site of the short hydrophobic sequence (4, 5 residues) located at the N-terminal adjacent to the loop formed by the S-S bond, which sterically hindered the substrate translocation (33, 34). These results were interpreted as substrate translocation being halted in a locally stable energy state generated by hydrophobic interactions between a short hydrophobic sequence in the substrate and the lateral gate under a condition where completion of protein translocation was inhibited. This situation is very similar to the VemP arrested state observed in this study. Considering the site of the VemP translation arrest (the codon for Asn-156 was located at the P site of the ribosome (18)) and the complicated secondary structures that the nascent VemP exhibits in the ribosome exit tunnel (23), approximately 100 residues of the N-terminal region of VemP would be exposed outside the ribosome. In this situation, the SHS is the most C terminally located hydrophobic segment in the aforementioned extraribosome region of the arrested VemP (Fig. 3*A*, left). Hence, the SHS in the arrested VemP would interact with the lateral gate. The proximity between the SHS and the ribosome tunnel exit site (approximately 20–30 residues in this state; Fig. 10*A*) may prevent the SecA ATPase from functioning from the cytosolic side, resulting in the increased dependence of the TTAC on SecD/F.

Replacement of the SHS by QNQN cancels the translation arrest-stabilizing effect observed in all three *trans*-factor mutants (Fig. 9). The reduced hydrophobic interaction between SecY and QNQN may enable VemP to move more freely up and down the channel by Brownian motion, which could allow VemP to use a pushing force from the cytoplasmic side (by SecA) and a pulling force from the periplasmic side (by SecD/F), or promote the VemP translocation by its partial folding in the periplasm, leading to the TTAC, even in the absence of the SecD/F or Ppi/YfgM complexes.

### Role of Arg-85 residue in the control of translation arrest

The introduction of Arg, but not Lys, residues into the SHS stabilized the VemP arrested state (Fig. 6, *C* and *D*), and the distance between the SHS and Arg-85 is important for TTAC (Fig. 7), suggesting a unique role for Arg in regulating TTAC. Recently, Collinson's group reported that the presence of Arg residues in a protein specifically slows the rate of its translocation, whereas a Lys residue has no such effect (35). They attributed this phenomenon to the deprotonation of the ε amino group of Lys in a translocating substrate at the SecA-SecY/E/G interface region, whose hydrophobic environment showed a reduction in the pKa of the ε amino group, and whose pH is higher as a result of proton translocation to the periplasm to form ΔpH. Since the guanidium group of Arg is always protonated (*i.e.* positively charged) even in such an environment, Arg residues in a polypeptide retard protein translocation due to electrical attraction attributed to the ΔΨ component of the proton motive force (PMF) (negatively charged on the cytoplasmic side). Indeed, we have previously observed that Arg-85 retards the translocation rate of an arrest-defective mutant (25). Such retardation of VemP translocation by Arg-85 may facilitate the hydrophobic interaction between the SHS and SecY and stabilize the pausing of the arrested VemP in the SecY/E/G channel.

Interestingly, the R85W mutation did not destabilize the VemP arrest state in the *secD1* strain (Fig. 9*C*). This could be explained as follows. Although the R85W mutation eliminates the positive charge-mediated reduction of the VemP translocation, the drastic reduction in the amount of SecD/F abolishes the PMF-driven promotion of the VemP translocation in a late step. These two opposing effects may cancel each other out, allowing the SHS alone to stabilize the VemP translocation. In addition, the Arg to Trp substitution at position 85 would increase local hydrophobicity, which may also contribute to the arrest stabilization.

### Role of (Hphi)xP motif in the regulation of translational arrest

The P67A mutation in the (Hphi)xP motif destabilizes the VemP arrested state in the Δ*ppiD* and Δ*yfgM* strains but not in the *secD1* strain with an undetectable level of SecDF (Fig. 9*C*), suggesting that this motif plays a different role from the other *cis* elements (the SHS and Arg-85) in the TTAC and could contribute to the SecD/F-dependent step(s). This fits well with the structural prediction that the (Hphi)xP motif is located near the P1 head subdomain of SecD (Fig. 10, *A* and *B*). The P67A mutation destabilizes the translation arrest state in the WT strain (Fig. 9*C*), indicating that the (Hphi)xP motif negatively regulates the TTAC under normal conditions. Because the P1 head subdomain of SecD contains the substrate-binding cavity (13), it is possible that the (Hphi)xP motif represses the binding of VemP to this domain. The PpiD/YfgM complex may promote proper binding of the VemP polypeptide to the P1 head subdomain in the supercomplex, SecY/E/G-SecD/F-PpiD/YfgM-VemP nascent chain. Further detailed studies, including the exact disposition of the (Hphi)xP motif in the supercomplex, will be required to understand the role of this motif in the TTAC.

### A proposed model for the molecular mechanism of VemP arrest cancellation

Based on the results and discussion in this and previous studies, we propose the following model for the molecular mechanism of the VemP arrest cancellation by the SecY/E/G-SecD/F-PpiD/YfgM supercomplex (Fig. 10*C*).

1) Targeting of VemP to the Sec translocon: The nascent VemP–ribosome complex is targeted to the cytoplasmic membrane by the signal recognition particle pathway (25) where the SecA-assisted translocation of VemP is initiated (step 1).
2) Transient arrest of the VemP translocation by the interactions between the VemP SHS and the SecY lateral gate: The steric hindrance by the VemP-synthesizing ribosome and the electrical attraction of the positive charge of Arg-85 halt the translocation of nascent VemP, with the hydrophobic interactions between the SHS and the lateral gate placing it at a specific position near the lateral gate (step 2). In this situation, SecA may be excluded and unable to function in VemP translocation.
3) Inhibition of the binding of VemP to the P1 head subdomain of SecD by the (Hphi)xP motif: While the translocation-halted VemP is situated near the P1 domain of SecD, the (Hphi)xP motif somehow prevents the nascent VemP from binding to the P1 head subdomain, and the arrested state is maintained transiently (step 3).
4) The PpiD/YfgM-dependent binding of the VemP polypeptide to the P1 head subdomain: The VemP polypeptide, whose translocation is halted, is presented to the P1 head subdomain through multiple interactions including those of PpiD with the SecD P1 head subdomain and VemP (step 4).
5) The TTAC by PMF-driven conformational changes of the P1 head subdomain: The VemP polypeptide is pulled toward the periplasm by the conformational change of the P1 head subdomain coupled with the influx of monovalent cations. The pulling force generated by this movement of VemP releases the translation arrest of VemP (step 5).

While the above working model is based on the structure predicted by AF2 and the biochemical results, steps 3 and 4 regarding the role of the (Hphi)xP motif are highly speculative and not well supported experimentally. Further detailed investigations are needed to validate this model. In particular, it would be important to identify the factors that physically or functionally interact with the (Hphi)xP motif in the supercomplex. Previous systematic *in vivo* photo-XL analysis against the entire region of VemP failed to detect any XL with other proteins for the (Hphi)xP motif region (25). Because the (Hphi)xP motif is present in the epitope sequence (59–73) of the anti-VemP antibodies used in the XL experiments, it is possible that XL products in this region were not efficiently recovered in the anti-VemP IP, and therefore, escaped detection. XL products can be detected by using VemP::FLAG-LacZα (used in this study) and anti-FLAG antibodies in XL experiments, which would lead to the identification of the (Hphi)xP motif interacting factors. According to our model, the (Hphi)xP motif may repress its binding to the P1 head subdomain of SecD. This possibility could be addressed by examining the binding ability of the isolated P1 head subdomain with a synthetic peptide containing the (Hphi)xP motif or its mutants. We postulate that SecA is not involved in VemP TTAC (step2). This can also be validated experimentally, which will provide further our understanding of the molecular mechanism of VemP TTAC.

## Conclusion and perspective

This study has addressed the molecular mechanisms underlying the TTAC of VemP. The SecD/F function can be monitored by the release efficiency of the translocation arrest of nascent VemP. This arrest can be attributed to the relatively weak hydrophobic interaction between nascent VemP and SecY and its release could be driven by the pulling forces associated with the substrate binding and the conformational change of the P1 head subdomain of SecD. The three *cis* elements (the (Hphi)xP motif, the SHS, and Arg-85), which are all required for proper TTAC regulation, are highly conserved among VemP orthologs (Fig. S6), suggesting that they would act together to monitor the SecD1/F1 function in *Vibrio* species. This mechanism would contribute to the maintenance of protein transport activity in *Vibrio* species growing in various salt environments by real-time monitoring of chan,ges in the Na^+^-driven SecD1/F1 activity in response to changes in the surrounding salt environment and feedback upregulation of H^+^-driven SecD2/F2 expression.

*Vibrio* species include many pathogenic bacteria such as *Vibrio cholerae*, *Vibrio parahaemolyticus*, and *Vibrio vulnificus*, all of which carry the *vemP-secD2/F2* operons and presumably possess the VemP translation arrest-coupled expression system of SecD2/F2 (18). Upon infection of a host, the salinity environment surrounding these pathogenic bacteria should change significantly. Hence, the regulation of SecD2/F2 expression by the VemP ortholog would be important not only for their survival and maintenance of protein export ability in the host but also for their virulence. Therefore, the identification and characterization of *cis* and *trans* elements involved in the stability of the translation arrest of VemP orthologs in the pathogenic *Vibrio* species might provide a clue to the prevention of pathogenic *Vibrio* infections.

Although the translation arrest-coupled–monitoring systems of protein export activity have been thought to be a specialized mechanism present only in a limited number of bacteria (16), the recent reports strongly suggest that they are used as mechanisms to regulate the expression of the Sec-related factors in a wide range of microorganisms (21, 22). Hence, elucidation of the regulatory mechanisms is becoming increasingly important. To fully understand the molecular mechanism of the translation arrest mediated real-time feedback regulation, it is crucial to understand the mechanism of not only the establishment of translation arrest but also its cancellation. Recently, a series of cryo-EM structures of ribosomes with arrested nascent polypeptides have been determined (23, 36, 37, 38), providing a molecular basis for the establishment of the translation arrest of the nascent chains. However, our knowledge of the molecular basis for the translation arrest cancellation is still very limited (39), although the concept that physical pulling force is necessary to release the arrest has been proposed (40, 41). In this and previous studies (25, 27), we have shown that the VemP arrest cancellation is tightly regulated by sophisticated interactions between multiple *cis* elements and *trans* factors including SecY. Further detailed studies, including a cryo-EM analysis of the ribosome-VemP-SecY/E/G-SecD/F-PpiD/YfgM supercomplex and molecular dynamics simulations based on the proposed model structure, are required to validate our conclusion. The studies on VemP, including the current study, will hopefully provide a compass for studying the molecular mechanisms of the arrest cancellation of other monitoring polypeptides.

## Experimental procedures

### Bacterial strains, plasmids, and primers

*E. coli* K12 strains, plasmids, and primers used in this study are listed in Tables S1–S3, respectively. Details of the strain and plasmid construction are described in Supporting Experimental procedures.

### Media and bacterial cultures

*E. coli* cells were grown in L-rich medium (10 g/L bacto-tryptone, 5 g/L bacto-yeast extract, 5 g/L NaCl; pH adjusted to 7.2 with NaOH) or M9 synthetic medium (without CaCl_2_; (42)) supplemented with maltose (final 0.2%), glycerol (final 0.4%), all amino acids (except Met and Cys; final concentration 20 μg/ml each). Final concentrations of 50 μg/ml Amp, 20 μg/ml chloramphenicol (Cm), 25 μg/ml kanamycin, 25 μg/ml tetracycline, and 50 μg/ml spectinomycin were added as appropriate for growing plasmid-bearing cells and selection of transformants and transductants. For the VemP-Bla reporter assay, *E. coli* cells were grown in L-medium supplemented with either 10 or 25 μg/ml Amp (see below). Bacterial growth was monitored with Mini photo 518R (660 nm; TAITEC Co).

### Antibodies

Anti-Myc (9E10), anti-FLAG M2, and anti-beta lactamase [8A5.A10] antibodies were purchased from Santa Cruz Biotechnology (, Sigma-Aldrich, and GeneTex, respectively. Anti-PpiD antibody (43) was a gift from M. Müller (University of Freiburg). Anti-SecD (25), anti-VemP (18), anti-SecY (44) and antimaltose binding protein (45) antibodies were described previously.

### *In vivo* stability assay of the arrested state of VemP

The procedure was used in Figures 1, *B* and *C*, 3, *B* and *C*, 6, *B* and *D*, 7, 8*D*, 9*C*, S1*C*, S4*B*, and S11. Cells were first grown at 30 °C in M9-medium supplemented with 2 μg/ml thiamine, 0.4% glycerol, 0.2% maltose, all amino acids (except Met and Cys) until early log phase. After induction with 1 mM IPTG and 2 mM cAMP for 15 min, cells were pulse-labeled with 370 kBq/ml [^35^S]Met (American Radiolabeled Chemicals, Inc) for 30 s. At appropriate time points after the addition of excess nonradioactive Met (final conc. 250 μg/ml), a portion of the cultures were withdrawn, and total cellular proteins were precipitated with 5% trichloroacetic acid, washed with acetone, and solubilized in SDS buffer (50 mM Tris–HCl (pH 8.1), 1% SDS, 1 mM EDTA, and 0.1 mM Pefabloc SC (Sigma-Aldrich)). Samples were then diluted 33-fold with Triton buffer (50 mM Tris–HCl (pH 8.1), 150 mM NaCl, 2% Triton X-100, 0.1 mM EDTA). After clarification, samples were incubated with appropriate antibodies (either anti-VemP, anti-maltose binding protein, or anti-FLAG M2 gel) and nProtein A Sepharose 4 Fast Flow (GE healthcare, UK Ltd) overnight at 4 °C with slow rotation. Proteins bound to antibody/protein A-Sepharose beads were recovered by centrifugation, washed with Triton buffer and then with 10 mM Tris–HCl (pH 8.1), and eluted by incubation at 25 °C for 5 min followed by 98 °C for 10 min in SDS-sample buffer (62.5 mM Tris–HCl (pH 6.8), 2% SDS, 10% glycerol, 5 mg/ml bromophenol blue). Isolated proteins were separated by SDS-PAGE and visualized with BAS1800 or BAS5000 phosphor-imager (GE healthcare). When VemP-F_3_M and VemP::FLAG-LacZα were analyzed, the percentages of arrested VemP were calculated using the following equations based on the numbers of Met: arrested VemP (%) = 100 × [AU+ 2.5 × AP]/[(AU+ 2.5 × AP) + (2.5 × FM)] (in Figs. 1*B*, 3, *B* and C, 6, *B* and *D*, 7 and 9*C*) and arrested VemP (%) = 100 × [AU+ 2 × AP]/[(AU+ 2 × AP) + (2 × FM)] (in Fig. 8*D*), where AU, AP, and FM are the intensities of the respective bands.

### S-S XL experiments

The procedure was used in Figure 2, *B*–*D*, *F* and S5. Cells were first grown to mid-log phase in L-medium supplemented with Amp and Cm. After induction with 1 mM IPTG and 2 mM cAMP for 1 h, cells were chilled on ice and treated with final 0.25 mM Cu(Phe)_3_ on ice for 30 min, then treated with final 20 mM N-ethylmaleimide on ice for 30 min to quench the oxidant. Total proteins were acid-precipitated, washed with acetone, and solubilized in the SDS buffer. The samples were divided into two portions. One was subjected to IP with anti-FLAG M2 affinity agarose gel (Sigma-Aldrich) according to the procedure described above. Proteins bound to the anti-FLAG M2 beads were recovered by centrifugation, washed with Triton buffer, and then with 10 mM Tris–HCl (pH 8.1), and eluted in the SDS-sample buffer by incubation at 25 °C for 30 min, followed by 37 °C for 5 min. The other portion was mixed directly with 2x the SDS-sample buffer. These IP-purified and total proteins were separated by SDS-PAGE and subjected to IB analysis as described above.

### IB analysis

This method was used in Figure 2, *B*–*D, F*, 4, *C* and *E*, 5*A*, S1*A*, S3, *B* and *C*, S5, *A*–*E*, S7 and S12, *A* and *B*. SDS-solubilized total proteins were separated by SDS-PAGE and electro-blotted onto a polyvinylidene difluoride membrane (Merck Millipore). The membrane was first blocked with 5% skim milk in PBST (PBS with Tween 20) and then incubated with anti-SecD (1/5000 dilution), anti-PpiD (1/50,000), anti-VemP (1/2,500), anti-FLAG (1/50,000), or anti-Myc (1/5,000) antibodies. After washing with PBST, the membrane was incubated with a horseradish peroxidase (HRP)-conjugated secondary antibody (1/5,000) (goat anti-rabbit IgG (H + L)-HRP conjugate (Bio-Rad Laboratories, Inc) for the first three or goat anti-mouse IgG (H + L)-HRP conjugate (Bio-Rad Laboratories) for the last two) in PBST. Proteins were visualized with enhanced chemiluminescence Western blotting detection reagents (GE Healthcare) or enhanced chemiluminescence Prime Western blotting detection reagents (GE Healthcare) and LAS3000 mini Lumino image analyzer (GE Healthcare).

### Growth of cells expressing a VemP-Bla reporter protein in a liquid medium containing a low concentration of Amp

This assay was used in Figures 4, *B* and *D*, 5, *B* and *C*, 6, *A* and *C*, 8*C*, 9, *A* and *B*, and S12, *A*–*C*. Overnight cultures of cells carrying a plasmid encoding a VemP-Bla derivative were diluted 100-fold with L-medium. Forty microliters of the diluted cultures was inoculated into 4 ml of L-medium supplemented with 20 μg/ml Cm, 1 mM IPTG, and 10 μg/ml Amp, and the cultures were grown with rotary shaking at 160 rpm at 30 ˚C. The turbidity of the cultures was measured hourly. The RGI of VemP-Bla derivatives was calculated according to the equation shown in Figure 5*C*. In the case of Figure 6*C*, 25 μg/ml Amp was used instead of 10 μg/ml Amp.

### Isolation of plasmids encoding VemP-Bla derivatives with altered stability of the arrested state by random mutagenesis

This procedure was used in Figure 5. Two kinds of plasmid libraries (groups 1 and 2) encoding VemP-Bla derivatives, in which L^78^ALF and L^80^F in the SHS were randomly mutagenized, respectively, were constructed from pHM1505 by site-directed mutagenesis using a pair of primers, LALF(random)-p and LALF(random)-c, and LAXXNT-p and LAXXNT-c, respectively. The plasmid libraries were introduced into the Δ*ppiD* strain (RM3122) and transformants were selected on L-Cm plates at 30 ˚C. They were purified, grown in L-liquid medium, spotted on an L-plates containing 20 μg/ml Cm-1 mM IPTG with or without 10 μg/ml Amp and incubated overnight at 30 ˚C to examine the Amp^R^ phenotype. Nucleotide sequences of the *vemP-bla* region on plasmids prepared from cells with either an Amp^R^ or Amp^S^ phenotype were determined using primers, RV-N and M4C. pYI7–pYI19, pYI40, pYI41, and pYI44–pYI52 were isolated from the group 1, and pYI20–pYI36, pYI38, and pYI53–pYI61 were from the group 2.

### Structural prediction with AlphaFold2

The structural models of the *E. coli* SecD/F-PpiD/YfgM-SecY/E/G supercomplex were constructed using the amino acid sequences of *E. coli* PpiD, YfgM, SecY, SecE, and SecG by ColabFold 1.3.0 (46) installed on a local workstation. From five structural models generated by the prediction, only a single model was shown because they were similar to each other. The coordinate of the *E. coli* SecD/F-PpiD/YfgM-SecY/E/G supercomplex is provided in PDB format in Supplementary file 4.

## Data availability

All data described are contained within the article.

## Supporting information

This article contains supporting information (48, 49, 50, 51, 52, 53, 54, 55, 56, 57, 58, 59, 60, 61, 62).

## Conflict of interest

The authors declare that they have no conflicts of interest with the contents of this article.
