# Supplementary material for: Translation arrest cancellation of VemP, a secretion monitor in Vibrio, is regulated by multiple cis and trans factors, including SecY
Source: J Biol Chem. 2024 Sep 2;300(10):107735. doi: 10.1016/j.jbc.2024.107735 (PMC11470409; doi:10.1016/j.jbc.2024.107735)
Supplement: Supporting Figures [file mmc2.pdf]

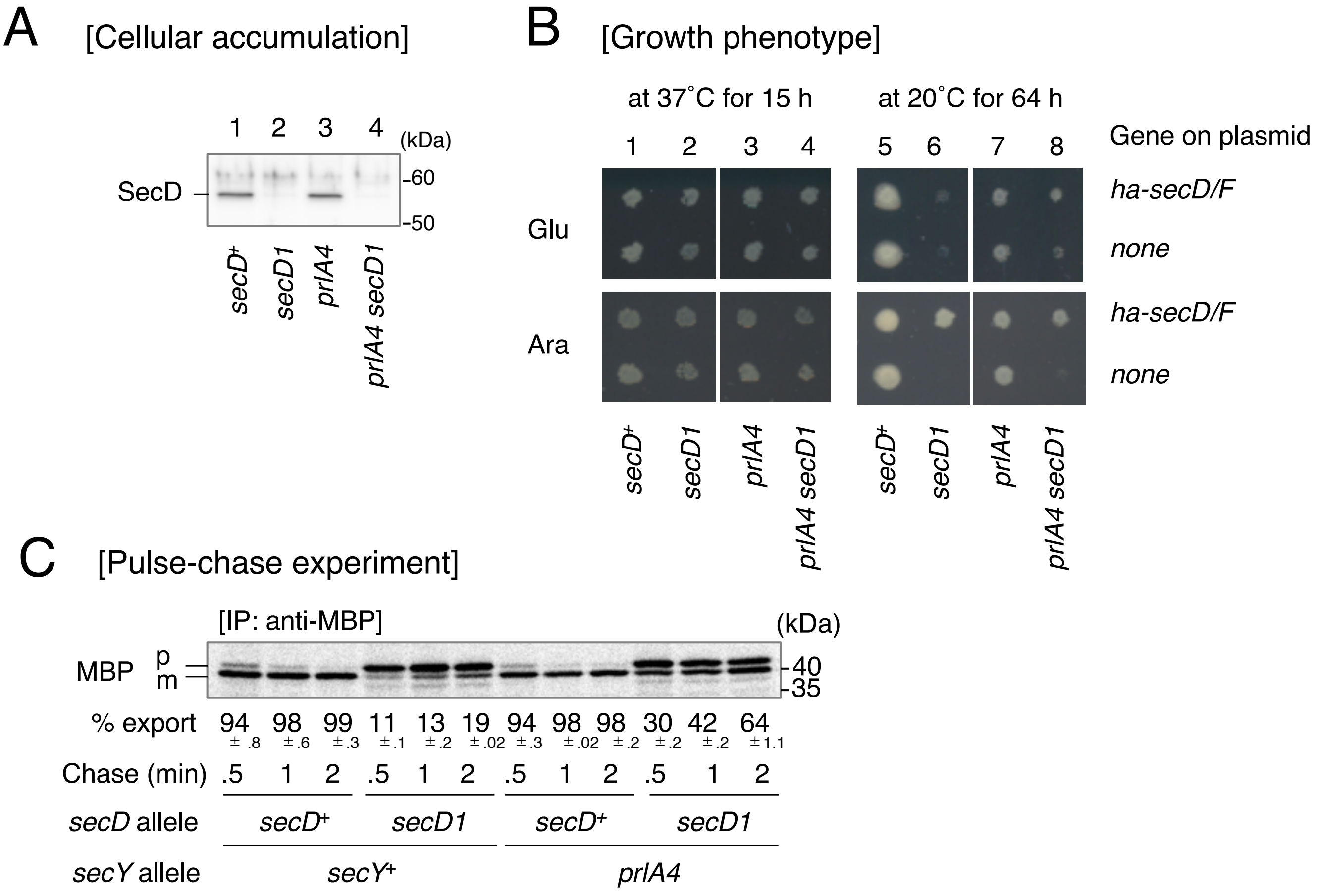

Figure S1. Characterization of a *prlA4-secD1* double mutant strain. A. Cellular accumulation of SecD. The four strains with genotypes indicated were cultivated in L medium until mid-log phase and total proteins were acid-precipitated, analyzed by SDS-PAGE and IB with anti-SecD antibodies. **B.** Cold sensitive growth defective phenotype of *prlA4-secD*1double mutant cells. Either cells, HM3203 (*secD*+), HM3199 (*secD1*), HM6833 (*prlA4, secD*+) or HM6835 (*prlA4, secD1*) carrying either pHM735 (*ha-secD/F*) or pBAD33 (none) were spotted on L-Amp-Cm-0.4% glucose (upper) and L-Amp-Cm-0.2 % arabinose (lower) and incubated at 37 ˚C for 15 h (left) and 20 ˚C for 64 h (right). The result shown is a representative of two biological replicates. **C**. MBP export activity. The four strains with the genotypes indicated carrying p-*vemP-f3m* were grown, induced, pulse-labeled and chased as in **Figure 1**. At each time point, total cellular proteins were acid-precipitated, subjected to IP with anti-MBP antibodies, and analyzed using SDS-PAGE, followed by phosphor-imaging. ***p*** and ***m*** indicate precursor and mature bands of MBP, respectively. Intensity of these bands were quantified and export efficiency of MBP were calculated according to the formula ([% export] = (1.5 x [m]) / ([p] + 1.5 x [m]) x 100). Means of % export with S.D. (N=2) are presented at the bottom of the gel. The result shown is a representative of two biological replicates.

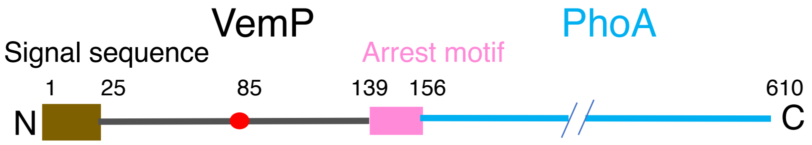

Translation arrest of VemP	PhoA activity
Stable	Low
Unstable	High

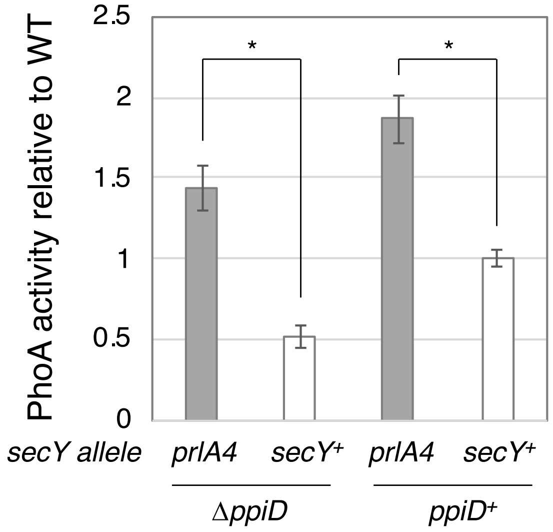

Figure S2. Monitoring of TTAC of VemP in mutant cells using a VemP-PhoA reporter. A schematic picture of VemP-PhoA reporter (27) and expected results. The four strains with genotypes indicated carrying the p-*vemP-phoA* were grown in L-Amp medium at 30˚C until mid-log phase, induced with 1 mM IPTG for 1h and measured PhoA activities of these cells according to the procedure described previously (27). Means of the activities with S.D. (N=2, biological replicates) are shown in the graph. An unpaired two-tailed Student's *t*-test was used to statistically compare the values between the groups. *$P < 0.05$. The *prlA4* mutant and Δ*ppiD-prlA4* double mutant cells possess higher PhoA activities than the corresponding isogenic $secY^+$ and Δ*ppiD*-$secY^+$ strains, respectively, strongly suggesting that the PrlA4 channel destabilizes the translation arrest of VemP.

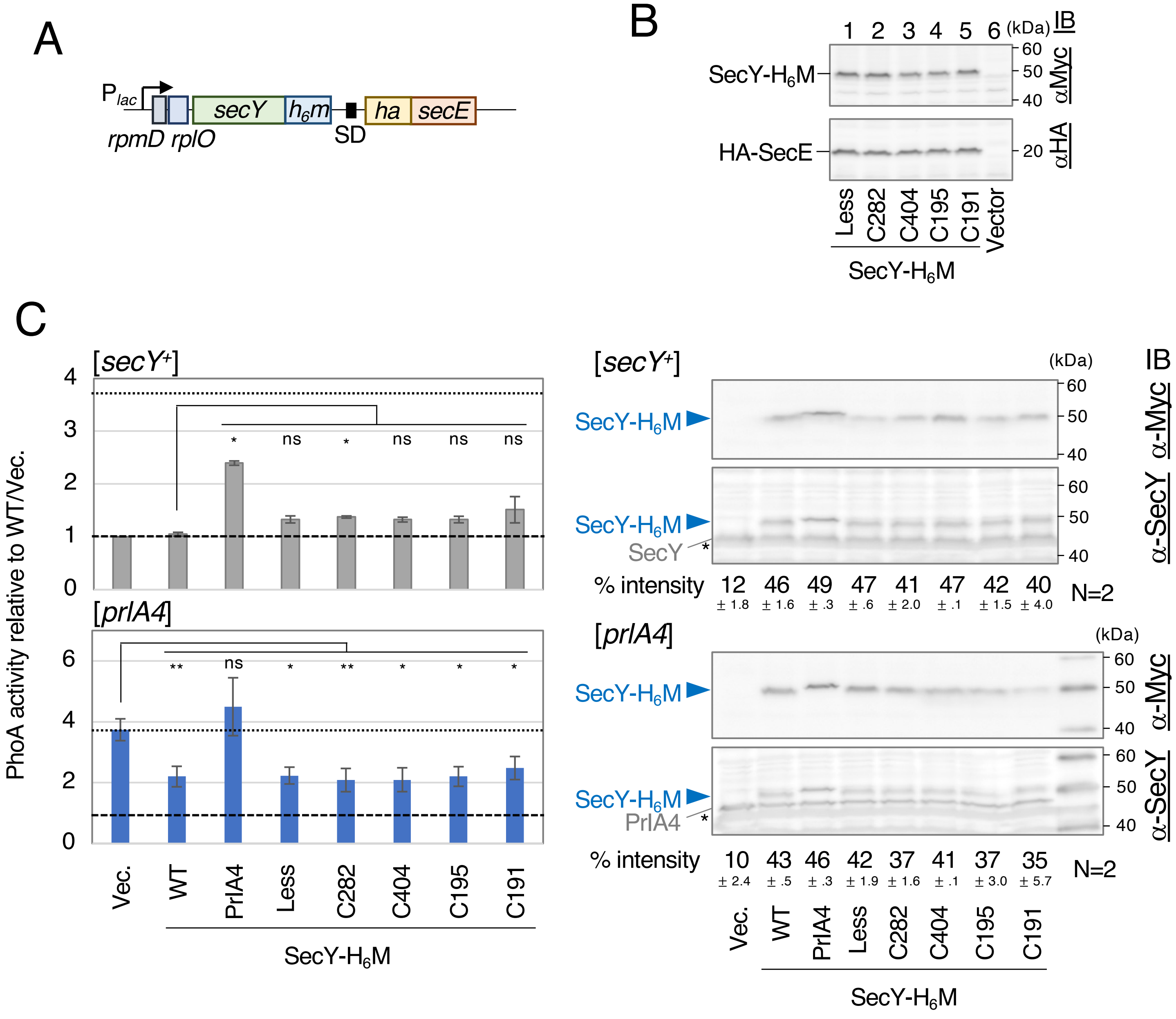

Figure S3. SecY(mono Cys) mutants used in disulfide bond XL experiments have a similar ability to maintain the arrested form of VemP as wild type SecY. A. A schematic picture of the plasmid, pHM1721 used for *in vivo* di-sulfide (S-S) bond XL experiments. *E. coli* SecY(Cys-less) in which two Cys residues (329, 389) were substituted to Ala was used as a start material for construction of mono-Cys derivatives, since the Cys-less derivative has been proven to exhibit normal function (55, 62). **B.** Cellular accumulation of SecY-H$_6$M derivatives and HA-SecE. Δ*ppiD* Cells carrying plasmids encoding both SecY-H$_6$M derivatives indicated and HA-SecE were grown in L-Cm medium at 30˚C until mid-log phase, induced with 1 mM IPTG-2 mM cAMP for 1 h. Total proteins were acid-precipitated and analyzed by SDS-PAG and IB with anti-Myc and anti-HA antibodies. The result shown is a representative of two technical replicates. Roughly similar amounts of the SecY-H$_6$M derivatives and HA-SecE were accumulated in all the cells. **C**. PhoA activities of cells expressing SecY-H$_6$M derivatives (left) and accumulation level of plasmid-encoded SecY-H$_6$M and chromosome-encoded SecY (right). Either Δ*ppiD-secY*$^+$/pHM1552 (p-*vemP-phoA, Spc*^{R}) or Δ*ppiD-prlA4* /pHM1552 cells were transformed with plasmids carrying *secY-h$_6$m* derivatives indicated. The obtained cells were cultivated in L-Cm-Spc-1 mM IPTG, 2 mM cAMP for 3 h. PhoA activities of crude lysate prepared from the cells was measured as in Figure S2B. Means of the relative PhoA activities of wild type cells (upper) and *prlA4* cells (lower) (the value of wild type cells expressing wild type SecY-H$_6$M was set to 1) with S. D. (N=2, biological replicates) are shown. An unpaired two-tailed Student's *t*-test was used to statistically compare the values between the groups. *P < 0.05, **P < 0.01, and *ns*, not significant. Total proteins from these cells after the cultivation for 3 h were acid-precipitated and analyzed by SDS-PAGE and IB with the indicated antibodies. Both bands corresponding to SecY-H$_6$M and SecY were quantitated and means of % intensity of SecY-H$_6$M (PrlA4-H$_6$M) bands with S.D. (N=2) are shown at the bottom of the gels. * indicates a protein non-specifically cross-reacting with anti-SecY antibodies. The result shown is a representative of two biological replicates. See Supporting result 2 in detail.

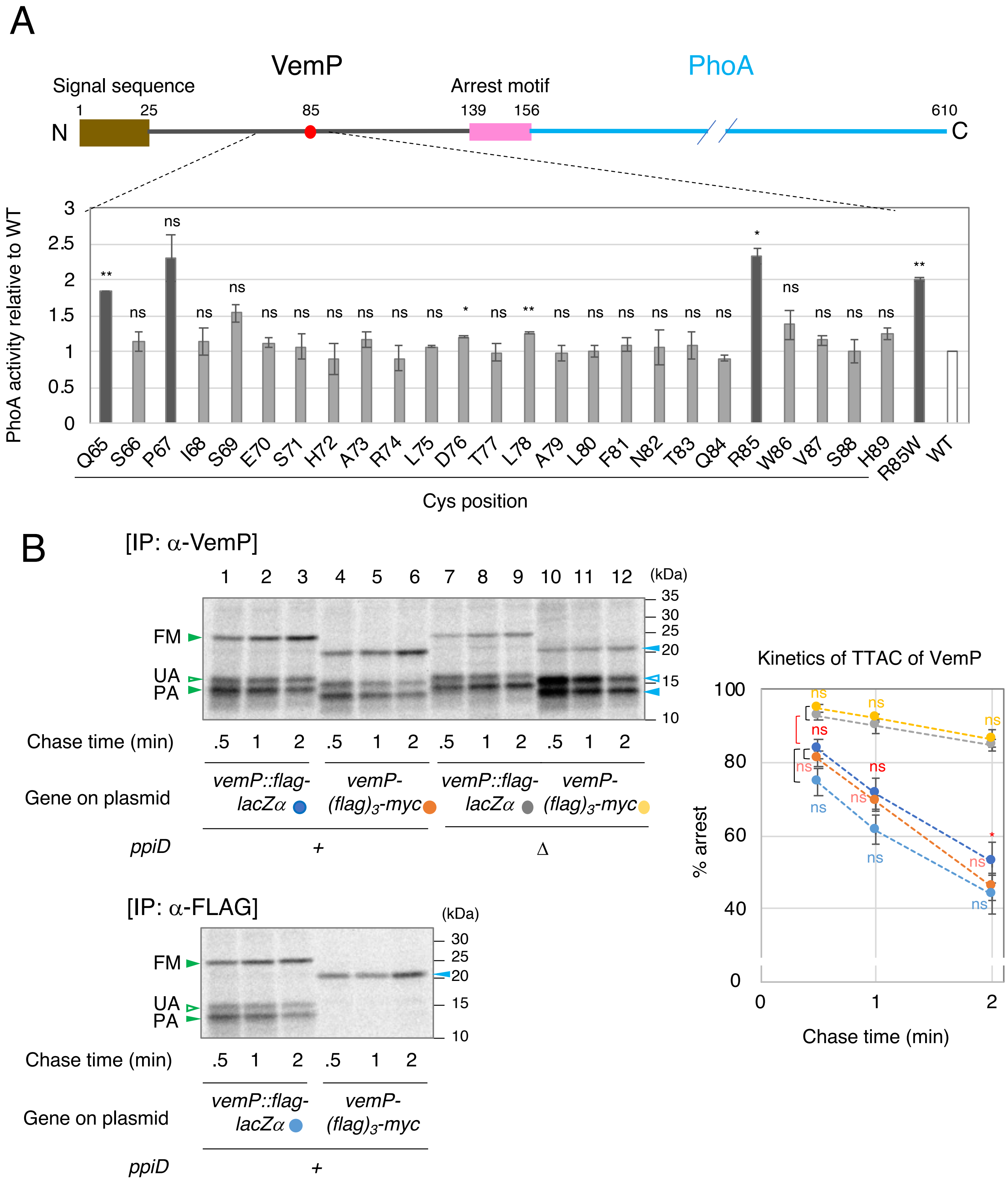

Figure S4. Characterization of VemP derivatives using S-S bond XL experiments. A. Cys-scanning analysis of the 65-89 region in VemP. Δ*ppiD* Cells carrying a plasmid encoding VemP(Cys at position indicated)-PhoA were cultivated in L-Amp with 1 mM IPTG at 30°C for 2.5 h and PhoA activites of total lysate prepared from the cells were measured as in **Figure S2**B. Means of relative PhoA activities of the cells expressing VemP(Cys)-PhoA derivatives (the value of Δ*ppiD* cells expressing wild type VemP-PhoA was set to 1) with S. D. (N=2, biological replicates) are shown. A paired two-tailed Student's *t*-test was used to statistically compare the values between the wild-type and each mutants. *$P < 0.05$, **$P < 0.01$, and *ns*, not significant. Cys-replacement of Qln-65 and Pro-67 as well as Arg-85 increased PhoA activities to the same level as that of the arrest-destabilizing R85W variant (25) but other Cys-mutants had almost as similar PhoA activities as that of the wild type. **B.** Kinetics of the TTAC of VemP::FLAG-LacZα. Either wild type (lanes 1–6) or Δ*ppiD* (lanes 7–12) cells carrying a plasmid with gene indicated were grown, induced, and pulse-chased as in **Figure 1**. Labeled VemP proteins were immunoprecipitated with anti-VemP antibodies and anti-FLAG M2 beads, analyzed and quantified as in **Figure 1**. The result shown is a representative of two biological replicates. Arrowheads in green and blue show VemP fragments generated from cells carrying *vemP::flag-lacZα* and *vemP-f₃m*, respectively. Means of % arrest of VemP with S.D. (N=2) were plotted against chase time (right). An unpaired two-tailed Student's *t*-test was used to statistically compare the values between the groups. *$P < 0.05$, **$P < 0.01$, and *ns*, not significant. Kinetics of the TTAC of VemP::FLAG-LacZα resembles that of VemP-F_3M, indicating that insertion of the FLAG-tag between positions 35 and 36 in VemP does not affect kinetics of the TTAC of VemP.

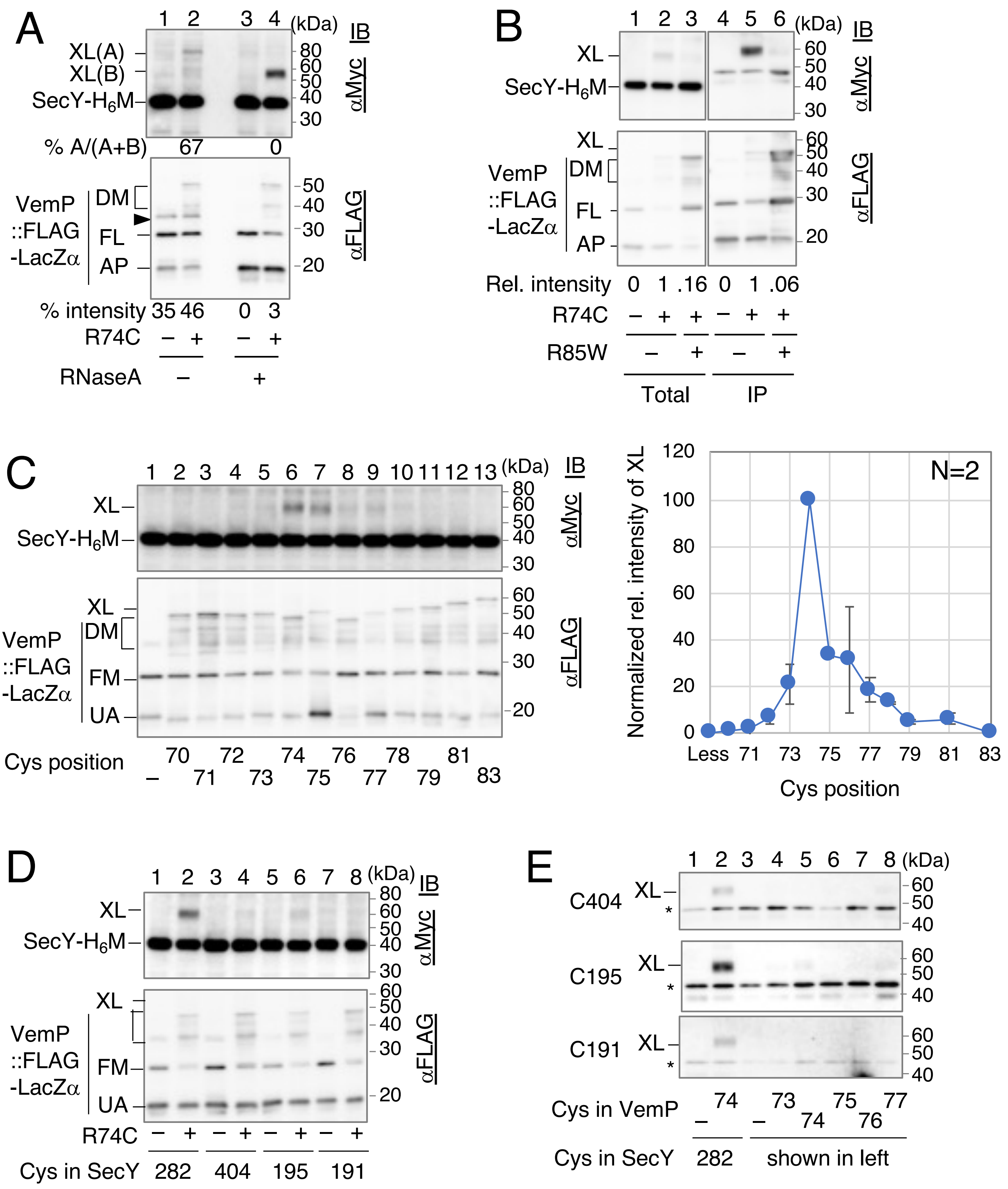

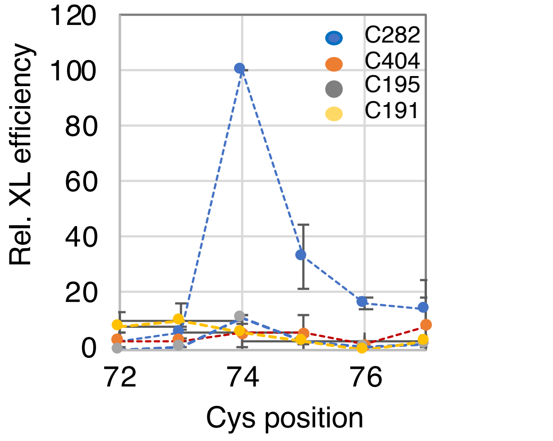

Figure S5. The nascent VemP polypeptide stops at a specific position in SecY. A. The XL product between SecY and VemP possesses a RNase-sensitive moiety. Δ*ppiD* Cells expressing both SecY(C282)-H$_6$M and VemP::FLAG (either R74 or C74) were subjected to the S-S bond XL experiments as in Figure 2B. Two sets of acid precipitated total proteins were solubilized with the 1x SDS sample buffer without 2-mercaptoethanol at room temperature for 30 min. One and the other treated with 0.2 mg/ml (final) RNaseA and H_2O (mock treatment) at 37˚C for 17 min, respectively. These samples were analyzed by neutral SDS-PAGE and IB with the antibodies indicated. The XL(A) and XL(B) in the upper gel presumably represent the XL-product between SecY(C282)-H$_6$M and VemP::FLAG (C74) with and without a tRNA moiety at its C-terminal, respectively. The intensities of the two XL bands was quantified and % [A]/([A] + [B]) was calculated and shown. The arrowhead in the bottom gel represents VemP::FLAG-tRNA.

(legend continued on next page)

The intensities of the two bands (the arrowhead and AP) was quantified and the % intensities of the VemP::FLAG-tRNA were calculated according to the formula [% intensity] = [the arrowhead] / ([AP] + [the arrowhead]) x100. The result shown is a representative of two biological replicates. See **Supporting result 3**–(1) in detail. **B.** The R85W mutation dramatically reduced formation of the XL-product. Δ*ppiD* Cells expressing both SecY(C282)-H_6M and VemP::FLAG (C74, either R85W or none) indicated were subjected to the S-S bond XL experiments, and analyzed by SDS-PAGE and IB with the antibodies indicated as in **Figure 2**B. Intensities of the XL products were quantified and efficiencies of X-linking were normalized as in **Figure 2**D (the intensity of XL between SecY(C282)-H_6M and VemP::FLAG (C74) was set to 1). The result shown is a representative of two biological replicates. See **Supporting result 3**–(2) in detail. **C**. Effects of Cys positions in VemP on S-S bond formation between SecY(C282)-H_6M and VemP::FLAG (mono-Cys). Δ*ppiD* Cells expressing all SecY(C282)-H_6M and HA-SecE and VemP::FLAG (Cys at indicated position) were treated with the oxidant and subjected to the acid-precipitation. Total proteins were solubilized with 1 x SDS-samples buffer without 2-mercaptoethanol and analyzed by SDS-PAGE and IB with the antibodies indicated as in **Figure 2**B. Intensities of the XL products were quantified and efficiencies of X-linking were normalized as in **Figure 2**D. Means of the normalized intensities (the intensity of XL between SecY(C282)-H_6M and VemP::FLAG (C74) was set to 1) with S. D. (N=2, biological replicates) are plotted against residue numbers where Cys residue was introduced (right). **D.** Effects of Cys position in SecY-H_6M on S-S bond formation between SecY(mono Cys)-H_6M and VemP::FLAG (C74). Δ*ppiD* Cells expressing SecY(mono Cys at the indicated position)-H_6M, HA-SecE and VemP::FLAG (either C74 or R74) were treated with the oxidant and subjected to the acid-precipitation. Total proteins were solubilized with 1 x SDS-samples buffer without 2-mercaptoethanol and analyzed by SDS-PAGE and IB with the antibodies indicated as in **Figure 2**B. The result shown is a representative of two biological replicates. **E.** Effects of Cys positions in VemP on S-S bond formation between SecY(mono Cys)-H_6M and VemP::FLAG (mono-Cys). Δ*ppiD* Cells expressing all SecY(mono Cys at the position indicated at the left)-H_6M, HA-SecE and VemP::FLAG (Cys at the position indicated at the bottom) were treated with the oxidant and subjected to IP with the anti-FLAG M2 beads, followed by SDS-PAGE and IB with anti-Myc antibody and quantitated as in **Figure 2**B. * indicates a protein non-specifically cross-reacting with anti-Myc antibody. The result shown is a representative of two biological replicates. In the bottom graph, the mean relative intensities of XL products with S.D. (N=2) are plotted against the number of VemP residues into which Cys was introduced.

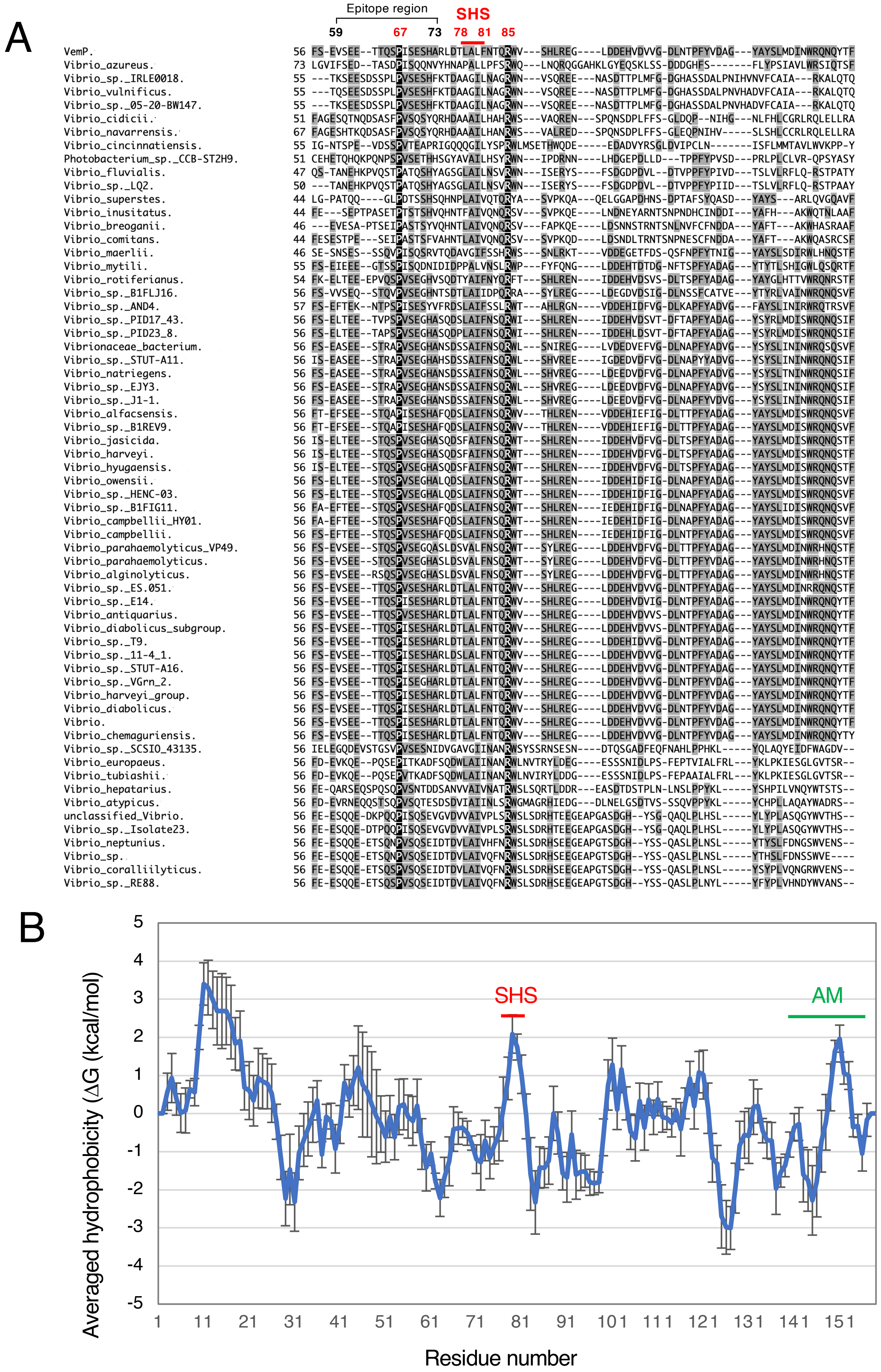

Figure S6. Conservation of *cis*-elements identified in this study. A. Amino acid sequence alignment of VemP orthologs from various *Vibrio* species. Amino acid sequences of 55–131 region (in the case of *V.a*VemP) from 62 kinds of VemP orthologs searched by using BlastP research site (https://blast.ncbi.nlm.nih.gov/Blast.cgi) are aligned with *V.a*VemP (top). Perfectly matched and conserved (>51%) residues are shown in black and gray shaded characters, respectively. The numbers at left and right at each line indicate the first and the last residue numbers of each VemP ortholog, respectively. Positions at 65, 67, 78-81, and 85 are highlighted as the corresponding red numbers. See **Supporting discussion 1** in detail. One (59–73) of the epitope regions for anti-VemP antibodies is also shown. **B.** Conservation of the short hydrophobic segment (SHS) of VemP among *Vibrio* species. The means of ΔG values calculated from 31 kinds of VemP orthologs including *V.a*VemP using the "Kyte and Doolittle" scale (window size is 5) are plotted against residues numbers. Error bars represent S.D. Positions of the SHS and the arrest motif (AM) are also shown as red and green bars, respectively. See **Supporting discussion 1** in detail.

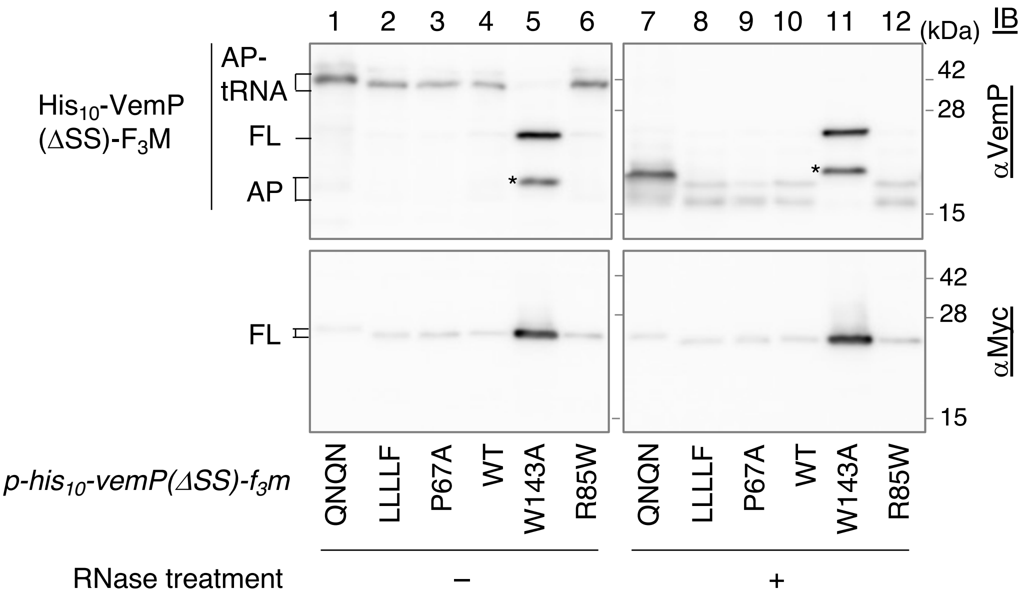

Figure S7. Newly identified *cis*-elements contribute to the TTAC of VemP. Δ*ppiD* Cells carrying p-*his*$_{10}$*-(ΔSS)vemP-f*$_3$*m* with the mutation indicated were grown, induced with 1 mM IPTG for 1 h as in Figure 2B. Total proteins were acid-precipitated, solubilized with sample buffer containing 2% SDS with (+) or without (-) 0.2 mg/ml RNase A at 37˚C for 15 min, separated with neutral SDS-PAGE and analyzed IB with the antibodies indicated. The result shown is a representative of two biological replicates. * indicates non-specifically cross-reacting band with anti-VemP antibodies. See Supporting result 4 in detail.

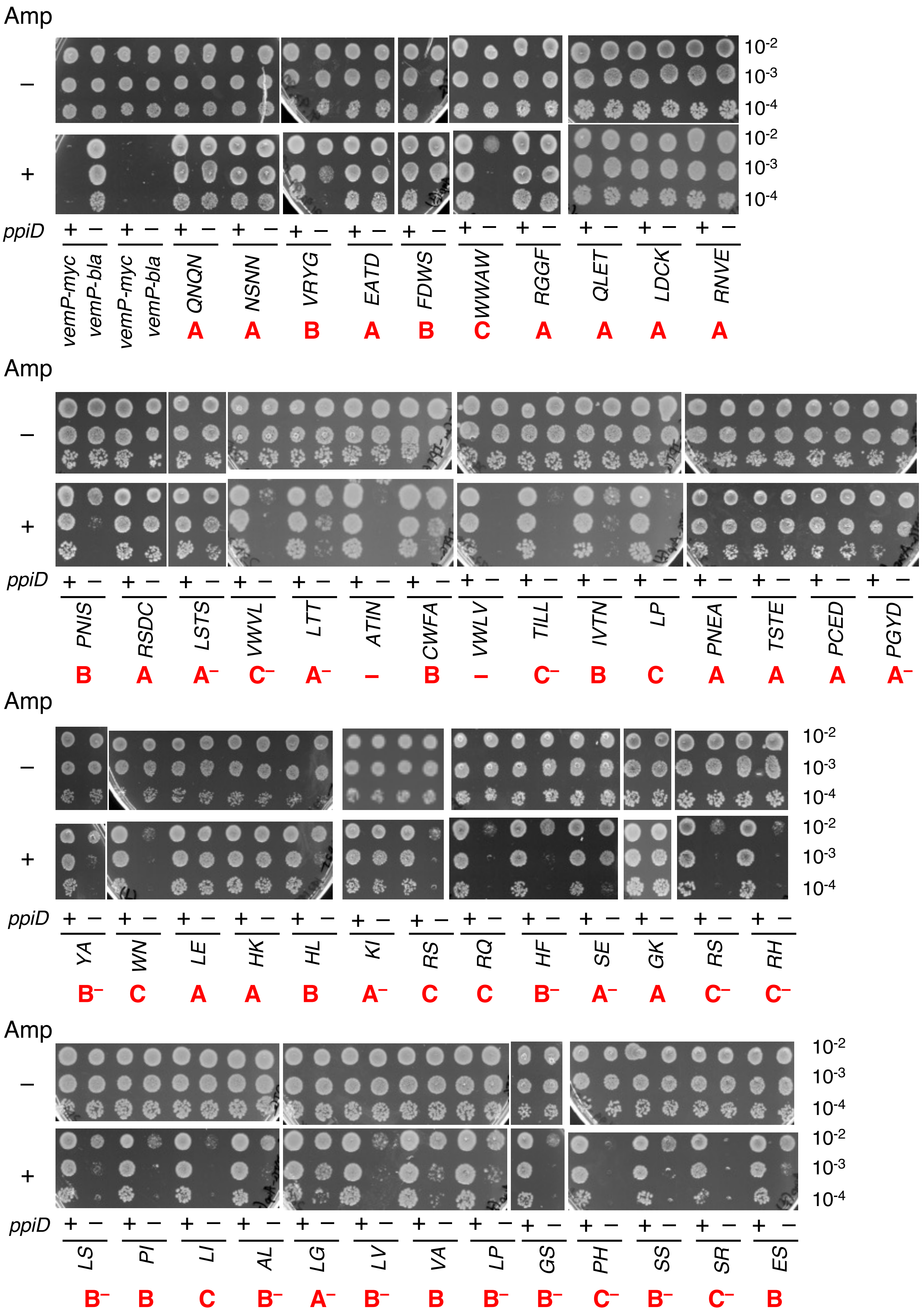

Figure S8. Growth phenotype of cells expressing VemP-Bla derivatives on L-plate. Either wild type or Δ*ppiD* strains carrying expression plasmids for VemP-Bla derivatives indicated were grown in L-Cm medium at 30 ˚C until mid-log phase, serially diluted with saline as indicated, spotted on L-plate supplemented with 1 mM IPTG, 20 µg/ml of Cm without (–) or with (+) 10 µg/ml Amp, and incubated at 30˚C for 16 h. The result shown is a representative of two biological replicates. A, B, and C represent that Δ*ppiD* cells expressing VemP-Bla derivatives grew well for 10^4-, 10^3-, and 10^2-fold diluted culture spots on the L-Amp plate, respectively. $A^–$, $B^–$, and $C^–$ indicate that the cells grew poorly at each dilution condition. – indicates that cells did not grow under any condition.

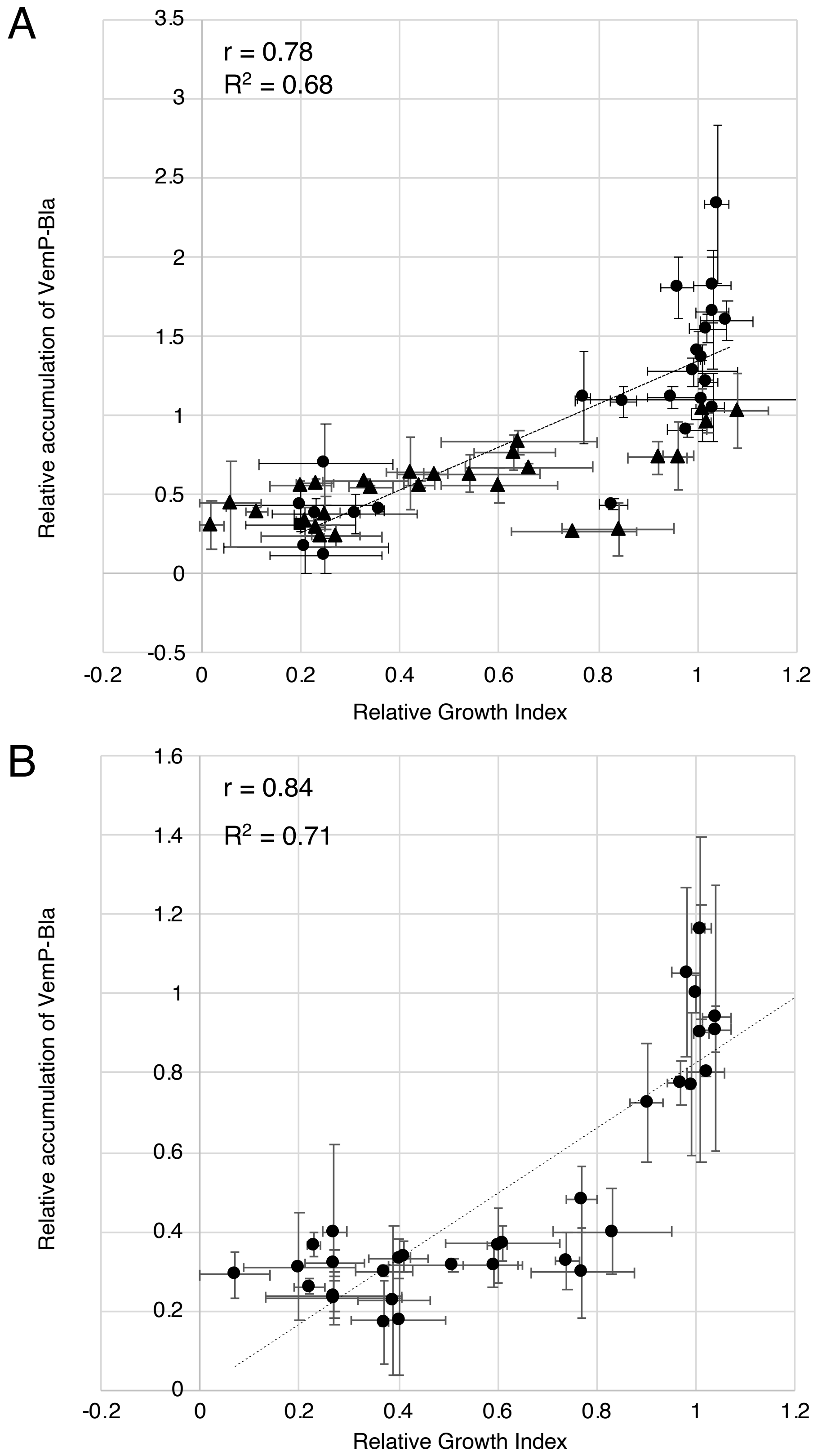

Figure S9. Relationship between RGI of Δ*ppiD* cells expressing VemP-Bla derivatives and their relative cellular accumulation. The RGI and relative accumulation level of each mutant isolated from the random mutagenesis analyses (**A**) shown in the fourth column from left and the rightest column, respectively, in **Table 1** (circles) and **Table 2** (triangles) and from the systematic analyses (**B**) shown in the third and fourth columns, respectively, in **Table 3** are plotted on horizontal and vertical axes, respectively, with S.D. (N=2, biological replicates). The open square and the closed square indicate the wild type cells expressing wild type VemP-Bla and the Δ*ppiD* cells expressing wild type VemP-Bla, respectively. In both graphs, the regression lines obtained by a least squares method using all the data are also shown with r and R^2 values. See **Supporting result 5** in detail.

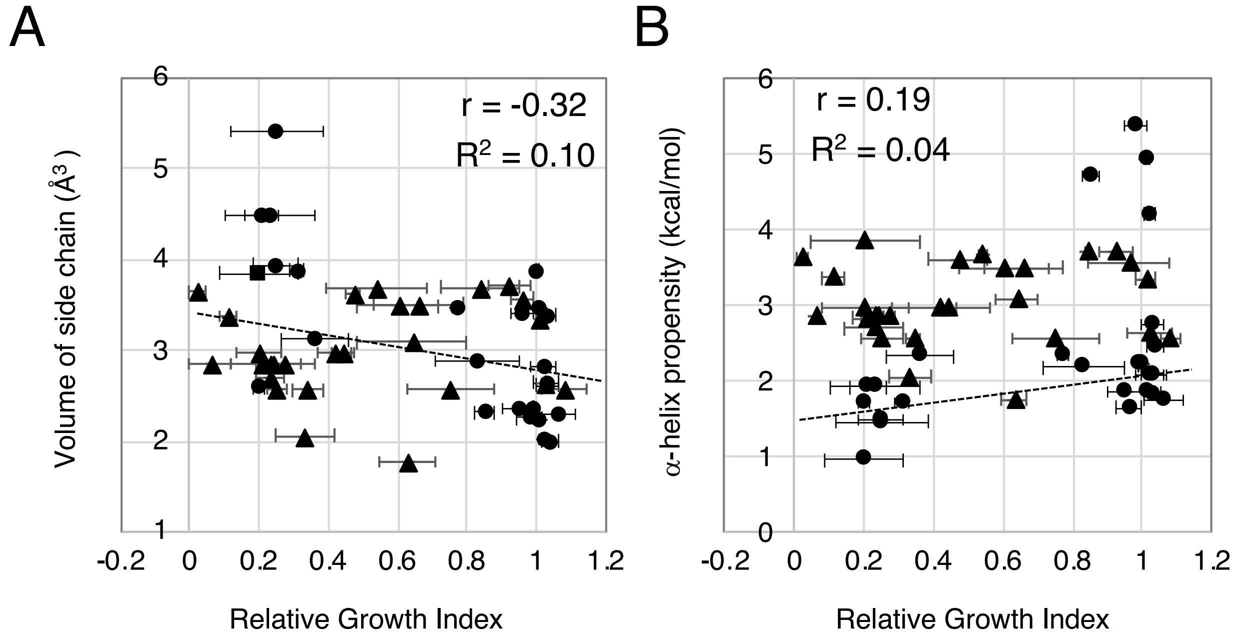

Figure S10. Relationship between RGI of Δ*ppiD* cells expressing VemP-Bla derivatives and other physical properties. The RGI and the calculated side-chain volume of SHS of each mutant isolated from the random mutagenesis analyses (**A**) shown in the second and fourth columns from left, respectively, in **Table S4–1** (circles), and **Table S4–2** (triangles) are plotted on horizontal and vertical axes, respectively, with S.D. On the same way, the RGI and the calculated α-helix propensity of SHS of each mutant isolated from the random mutagenesis analyses (**B**) shown in the second column from the left and the rightest column, respectively, in **Table S4–1** (circles) and **Table S4–2** (triangles) are also plotted on horizontal and vertical axes, respectively, with S.D. In both graphs, the regression lines obtained by a least squares method using all the data are also shown with r and R^2 values. See **Supporting discussion 3** in detail.

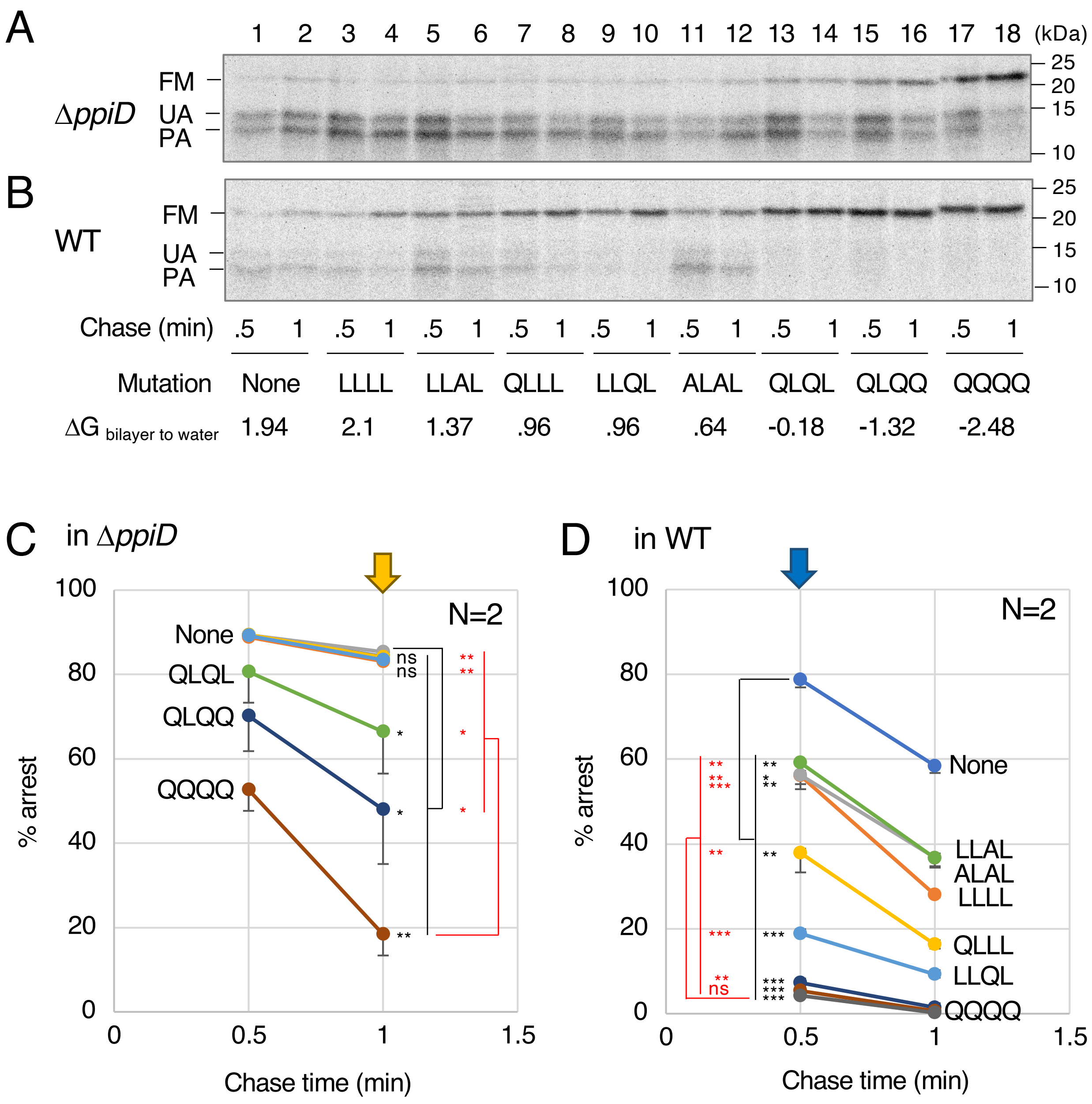

Figure S11. Pulse-chase experiments of either wild type or Δ*ppiD* cells expressing VemP-F_3M derivatives with different hydrophobicity in the SHS region. Either Δ*ppiD* (A) or wild type (B) cells carrying p-*vemP-f₃m* with the indicated mutation were grown, induced, pulse-chased and analyzed as described in Figure 1. The means of % arrested form of VemP in the Δ*ppiD* cells (C) and in the wild type cells (D) were plotted against the chase time (error bars marked only in the negative direction represent S.D. (N=2)). An unpaired one-tailed Student's *t*-test was used to statistically compare the values between the groups. $*P < 0.05$, $**P < 0.01$, $***P < 0.001$, and *ns*, not significant. Arrested forms of VemP derivatives with more hydrophobic SHS segments than the QLQL mutant ($\Delta G_{\text{bilayer to water}}$ = - 0.18 kcal/mol) retained stable in the Δ*ppiD* cells over the chase time. By contrast, arrested forms of VemP derivatives with more hydrophilic SHS segments than the ALAL mutant ($\Delta G_{\text{bilayer to water}}$ = 0.64 kcal/mol) were rapidly and almost completely converted to the full-length even at the 30 sec chase time in the wild type cells. These quantified data at the 1 min chase in the Δ*ppiD* cells (yellow arrow) and those at the 30 sec chase in the wild type cell (blue arrow) were used to generate Figure 6B. Note that data from the Δ*ppiD* cells expressing VemP(LLLL)-F_3M is not included in the graph of Figure 6B, because we have not confirmed reproducibility of the result.

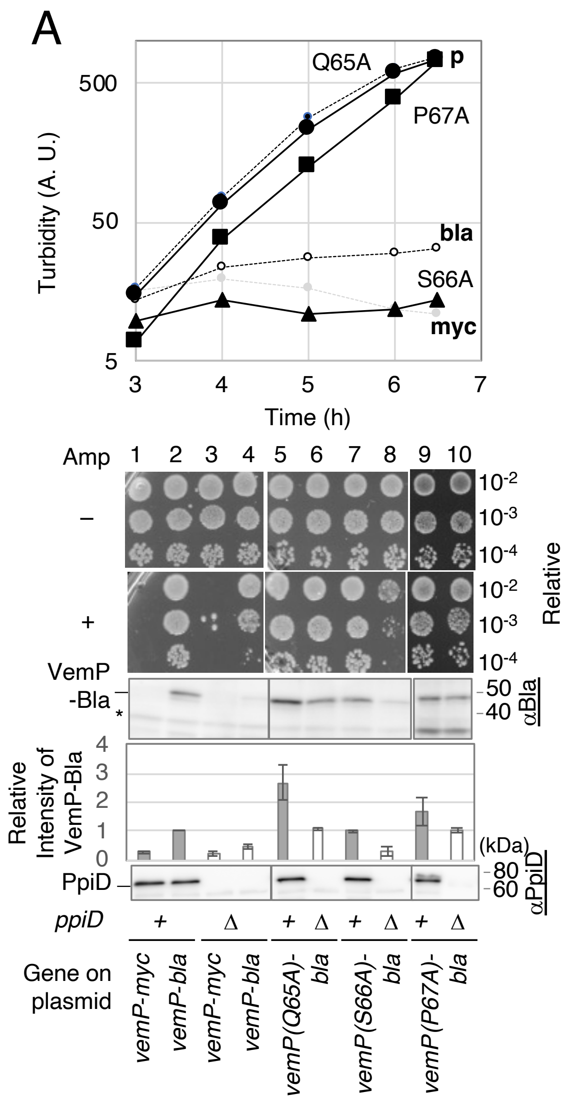

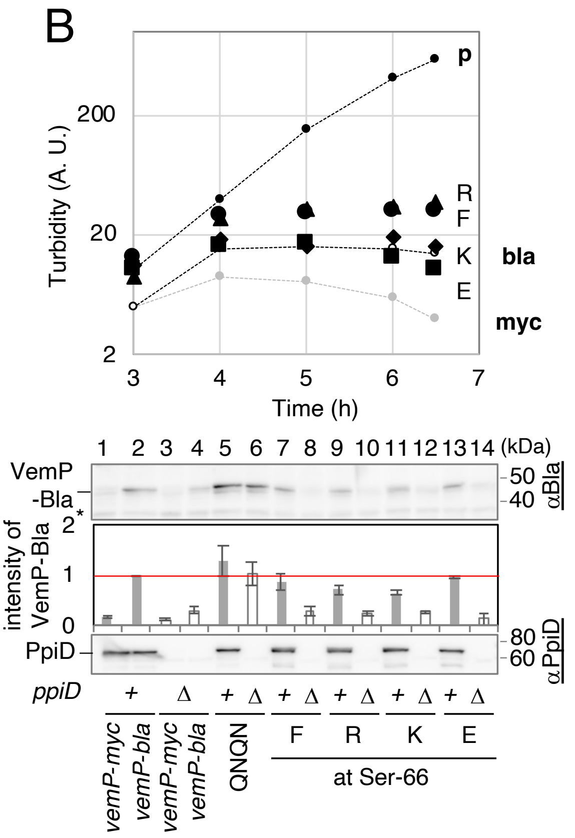

	RGI	RI of VemP-Bla
Ala	0.01 (±0.01)	0.30 (±0.11)
Phe	0.38 (±0.01)	0.29 (±0.08)
Arg	0.37 (±0.05)	0.26 (±0.04)
Lys	0.30 (±0.08)	0.28 (±0.02)
Glu	0.23 (±0.05)	0.13 (±0.11)

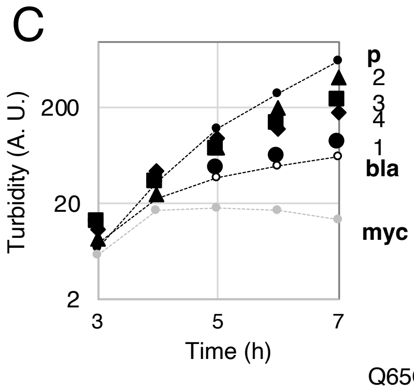

	Pro-67		Gln-65	
	GOP	RGI	GOP	RGI
Ala	+	0.93 (±0.02)	+	0.97 (±0.14)
Trp	+	0.93 (±0.01)	+	1.03 (±0.02)
Lys	+	1.02 (±0.0)	–	0.31 (±0.11)
Glu	+	0.99 (±0.05)	–	0.43 (±0.14)
Gly	+	0.96 (±0.07)	variable	0.70 (±0.20)
Leu	+	0.84 (±0.04)	+	0.99 (±0.02)

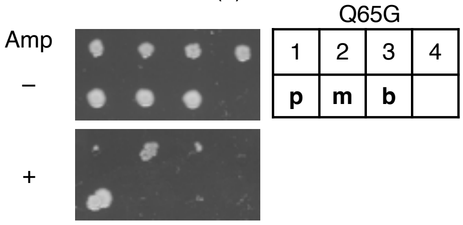

Figure S12. Mutational analysis of QSP region in VemP. A. Ala scanning analysis of the QSP region. Growth phenotypes of Δ*ppiD* cells expressing VemP-Bla derivatives with the mutation indicated in L medium with 10 μg/ml Amp (upper graph) on L-plate with (+) or without 10 μg/ml Amp (–) (middle panels) were examined as in Figure 4B, C, respectively. In A, growth curves of the control samples (wild type cells / p-*vemP-bla* (small closed circles), Δ*ppiD* cells / p-*vemP-bla* (small open circles), and Δ*ppiD* cells / p-*vemP-myc* (small gray circles) are also presented. * indicates a protein non-specifically cross-reacting with anti-Bla antibody. The results shown are representatives of two biological replicates. Relative accumulation level of their VemP-Bla derivatives were determined as in Figure 4C. Means of relative intensity of VemP-Bla bands with S. D. (N=2) are presented in the bottom graph. **B.** Growth phenotype of Δ*ppiD* cells expressing VemP-Bla mutants with the amino acid alteration indicated at position 66 in L medium with 10 μg/ml Amp were examined as in Figure 4B (upper graph). Relative accumulation level of their VemP-Bla derivatives were determined as in Figure 4C. Means of relative intensity of VemP-Bla bands with S. D. (N=2, biological replicates) are shown in the middle graph. The data regarding VemP(S66X) mutants shown in Figure S12A and B are summarized in the bottom Table. See Supporting result 6 in detail. **C**. Different growth phenotype of Δ*ppiD* cells expressing VemP(Q65G)-Bla mutant in liquid (upper) and on plate (lower). Growth of four different transformants carrying p-*vemP(Q65G)-bla* in L medium with 10 μg/ml Amp (upper) and on L-plate with or without 10 μg/ml Amp (lower) were examined as in Figure 4B and C. **p**, **m**, and **b** represent control cells, wild type / p-*vemP-bla* (positive), Δ*ppiD* / p-*vemP-myc* (negative), and Δ*ppiD* / p-*vemP-bla* (negative), respectively. The data regarding VemP(Q65X or P67X) shown in Figure 8A, C and Figure S12C are summarized in the Table (right). GOP means growth phenotype on L plate containing 10 μg/ml Amp. See Supporting result 7 in detail.
