# Supplementary material for: Translation arrest cancellation of VemP, a secretion monitor in Vibrio, is regulated by multiple cis and trans factors, including SecY
Source: J Biol Chem. 2024 Sep 2;300(10):107735. doi: 10.1016/j.jbc.2024.107735 (PMC11470409; doi:10.1016/j.jbc.2024.107735)
Supplement: Supporting Information [file mmc3.pdf]

Supporting information

Translation arrest cancellation of VemP, a secretion monitor in *Vibrio*, is regulated by multiple cis- and trans-factors, including SecY

Yuki Ikeda[1], Ryoji Miyazaki[2], Tomoya, Tsukazaki[2], Yoshinori Akiyama[1] and Hiroyuki Mori[1]

[1]Institute for Life and Medical Sciences, Kyoto University, Kyoto 606-8507, Japan; [2]Division of Biological Science, Graduate School of Science and Technology, Nara Institute of Science and Technology, Nara 630-0192, Japan

*For correspondence: hiromori@infront.kyoto-u.ac.jp (HM)
Tel. +81 75-751-3995; Fax +81 75-771-5699

Contents

Supporting results and discussion (pages S-2–S-5)
Supporting experimental procedures (pages S-6–S-7)
Table S1: Strains used in this study (page S-8)
Table S2: Plasmids used in this study (pages S-9–S-12)
Table S3: Primers used in this study (page S-13)
Table S4-1: Summary of the RGI and the SHS physical properties (Group 1) (page S-14)
Table S4-2: Summary of the RGI and the SHS physical properties (Group 2) (page S-15)

Figure S1. Characterization of a *prlA4-secD1* double mutant strain
Figure S2. Monitoring of TTAC of VemP in mutant cells using a VemP-PhoA reporter
Figure S3. SecY(mono Cys) mutants used in disulfide bond XL experiments have a similar ability to maintain the arrested form of VemP as wild type SecY.
Figure S4. Characterization of VemP derivatives using S-S bond XL experiments
Figure S5. The nascent VemP polypeptide stops at a specific position in SecY.
Figure S6. Conservation of *cis*-elements identified in this study
Figure S7. Newly identified *cis*-elements contribute to the TTAC of VemP.
Figure S8. Growth phenotype of cells expressing VemP-Bla derivatives on L-plate
Figure S9. Relationship between RGI of Δ*ppiD* cells expressing VemP-Bla derivatives and their relative cellular accumulation
Figure S10. Relationship between RGI of Δ*ppiD* cells expressing VemP-Bla derivatives and other physical properties
Figure S11. Pulse-chase experiments of either wild type or Δ*ppiD* cells expressing VemP-F_3M derivatives with different hydrophobicity in the SHS region
Figure S12. Mutational analysis of QSP region in VemP

Supporting information file 1: The coordinate of the *E. coli* SecD/F-PpiD/YfgM-SecY/E/G super complex (PDB format)

Supporting results and discussion

Supporting result 1. Characterization of a *prlA4-secD 1* double mutant strain.

The *secD1* mutant strain, in which the cellular accumulation level of SecD/F is reduced to undetectable levels (Figure S1A lane 2), exhibited a cold-sensitive growth defect (Figure S1B, lane 6) and strong maltose binding protein (MBP) export defects (Figure S1C, lanes 4-6) (48)). The *prlA4-secD 1* double mutant cells were also unable to grow at 20°C, and this *Cs* phenotype was suppressed by the expression of SecD/F from a plasmid as in the *secD1* mutant cells (Figure S1B, lower panels, lanes 6 and 8). Thus, although the additional introduction of the *prlA4* mutation partially rescued the MBP export defect in the *secD1* mutant (Figure S1C, lanes 10-12), it did not completely suppress the phenotypes attributed to the *secD1* mutation.

Supporting result 2. The SecY(mono-Cys)-H$_6$M derivatives used in the S-S XL experiment retain comparable activity to wild-type SecY in maintaining the VemP translation arrest.

The ability of the SecY(mono-Cys)-H$_6$M derivatives used in the XL experiments to maintain the VemP arrested state was examined using the VemP-PhoA reporter system (See Figure S2). SecY complexed with SecE is stable in a cell, but when it fails to form a complex with SecE , it is rapidly eliminated by the FtsH protease (49). Therefore, when SecY-H$_6$M is expressed solely from a plasmid, the plasmid-born SecY-H$_6$M competes with the chromosomally encoded SecY for interaction with SecE, resulting in only a fraction of the SecY/E/G complex in a cell containing plasmid-born SecY-H$_6$M. Indeed, when SecY-H$_6$M derivatives were expressed in either the wild-type or *prlA4* mutant strain, approximately 30–40% of the SecY in the cells was replaced by the SecY-H$_6$M, regardless of the introduced Cys in SecY (Figure S3C, right panels). Thus, the PhoA activities of these strains are expected to represent the sum of the activity of the chromosomally encoded SecY-containing translocon and that of the plasmid-born SecY-H$_6$M-containing translocon.

Compared to the PhoA activity observed in the *secY*$^+$ strain, that in the *prlA4* strain, in which the VemP translation arrest is destabilized, was approximately 3.5-fold higher (lower graph, far left), whereas the expression of wild-type SecY-H$_6$M in the *prlA4* strain reduced the PhoA activity to approximately two-fold, probably due to the replacement of a fraction of the chromosomally encoded PrlA4 by the wild-type SecY-H$_6$M (lower graph, second bar from the left). Conversely, expression of PrlA4-H$_6$M in the wild-type strain resulted in a two-fold increase in the PhoA activity (upper graph, third bar from the left). These results support the aforementioned hypothesis described. The PhoA activities of the wild-type cells expressing either the SecY(mono Cys)-H$_6$M derivatives or SecY(Cys-less)-H$_6$M were not significantly different from those of the cells expressing wild-type SecY-H$_6$M. The degree of the decrease in the PhoA activities of the *prlA4* cells expressing the SecY(mono Cys)-H$_6$M derivatives was also similar to that of the *prlA4* cells expressing wild-type SecY-H$_6$M. In the XL analysis, the stability of the VemP translation arrest in the cells expressing the SecY(mono-Cys)-H$_6$M derivatives was similar to that of the cells expressing wild-type SecY-H$_6$M. Thus, the difference in S-S XL formation efficiency between the SecY(mono-Cys)-H$_6$M mutants (Figure 2F) would reflect the difference in the distance (and/or orientation) between Cys74 in VemP and the Cys residues introduced into SecY.

Supporting results 3: SecY(C282)-H$_6$M forms an S-S bond with a nascent VemP::FLAG(C74)

We verified whether the partner of the XL band with SecY(C282)-H$_6$M observed in Figure 2B is the nascent or the full-length VemP::FLAG (C74)-LacZα via the following two experiments.

(1) Detection of VemP- tRNA (See Figure S5A)

After the oxidant treatment to induce S-S bond formation between SecY(C282)-H$_6$M and VemP::FLAG (C74), as shown in Figure 2B, all cellular proteins were acid-precipitated, solubilized in a neutral buffer containing 1% SDS, treated with or without RNase A, and analyzed by neutral SDS-PAGE and IB with anti-Myc and anti-FLAG antibodies. In the RNase A-untreated samples, two XL bands, a major XL(A) and a minor XL(B), which cross-reacted with anti-Myc antibodies, were detected (lane 2). After RNase A treatment, the XL(A) band disappeared, and instead, the intensity of the XL(B) band increased (lane 4), suggesting that the XL(A) band represented an RNA-containing molecule, i.e., SecY-H$_6$M x VemP::FLAG-tRNA, which was converted to

X(B) upon RNase A treatment. The percentage of VemP-tRNA (black arrowhead in the lower gel) among all nascent VemP polypeptides was calculated based on the intensities of the two bands cross-reacting with anti-FLAG antibodies (AP and black arrowhead), indicating that a significant fraction of the arrested VemP-tRNA lost the tRNA moiety during the experimental manipulation even without RNaseA treatment. Furthermore, RNaseA treatment almost completely removed the tRNA moiety. Thus, the XL(B) in lane 2 would be a tRNA-less degradation product of the XL(A) generated during the sample preparation. These results strongly suggest that the observed XL product was formed between the nascent VemP::FLAG (C74) polypeptide and SecY(C282)-H_6M.

(2) Effect of the R85W mutation on the XL formation (See Figure S5B)

The Arg-85 residue in VemP plays an essential role in maintaining the VemP arrested state in the SecY/E/G translocon, and the Trp-substitution of this residue dramatically destabilizes its translation arrest (25). Therefore, we examined the efficiency of XL formation between VemP::FLAG (C74, R85W) and SecY(C282)-H_6M. Consistent with previous reports, the R85W mutation decreased and increased the arrested and full-length forms of the VemP::FLAG (C74) mutant, respectively, (lower gel, lanes 3 and 6), and concomitantly, decreased the efficiency of XL formation (upper gel, lanes 3 and 6). This result also supports the hypothesis that the XL partner represented the nascent VemP polypeptide.

Supporting discussion 1: High conservation of the VemP *cis*-elements among *Vibrio* species (Related to Figure S6)

The Arg-85 residue is completely conserved among the VemP orthologs of nine *Vibrio* species (25). We expected that the novel two *cis*-elements identified in this study (SHS, and Gln-65 and Pro-67 residues in the (Hphi)xP motif) would also be highly conserved, given their functional importance. We then confirmed this by comparing the amino acid sequences of various VemP orthologs. First, we selected the top 500 VemP ortholog sequences by BlastP search (https://blast.ncbi.nlm.nih.gov/Blast.cgi) using the entire *V. alginolyticus(Va)*VemP sequence as a query. To compare VemP orthologs from evolutionarily distant and diverse species, we finally selected and used 62 VemP orthologs for homology comparison with *V.a*VemP according to the following two criteria; 1) only one ortholog sequence with the lowest E-value was selected as a representative for multiple sequences from the same species. 2) only VemP orthologs with signal sequences were selected by Signal-P 5.0 analysis. The results of the alignment of sequences corresponding to the 56–131 region of *V.a*VemP, which includes three *cis*-elements characterized in this study, are shown in Figure S6A. As expected, residues Pro-67 and Arg-85 were completely conserved in all orthologs examined. Residue Gln-65 was also largely conserved but was replaced in some orthologs by Ser, Thr, Arg, Pro, and Gly residues, all of which are substitutes in *V.a*VemP (**Figure 8**A, B), and therefore, will not affect the VemP arrest.

As shown in **Figure S6**A, the SHS among the selected VemP orthologs primarily comprised hydrophobic amino acid residues, supporting our working model. To clearly demonstrate the conservative nature of the SHS among VemP orthologs, we performed more quantitative analyses. First, we obtained 241 VemP ortholog sequences by a Quick BLAST search, which can align sequences with few gaps and insertions, using the entire *V. alginolyticus* VemP sequence. We selected 30 types according to the aforementioned two criteria and calculated the hydrophobicity at each amino acid residue position in the VemP orthologs using the "Kyte and Doolittle" scale (window size 5) (47). Because the sequence alignments obtained from the 30 orthologs contained few insertions and deletions (data not shown), we calculated the mean and standard deviation of the hydrophobicity at each amino acid residue position using a total of 31 orthologs (the selected 30 + *V.a*VemP) and plotted these data against the residue number of *V.a*VemP (**Figure S6**B). The region corresponding to the SHS exhibited a weak but distinct hydrophobic peak in the plot, and the standard deviation of this region was smaller than that of other short hydrophobic regions (11–25, 43–47) but comparable to that of the highly conserved arrest motif. Therefore, we concluded that the hydrophobic feature of the SHS was conserved among VemP orthologs.

Interestingly, neither an insertion nor a deletion of amino acid residue was observed between Gln-65 and Arg-85 when the 63 orthologs are aligned (**Figure S6**A). In addition to the cooperative function between SHS and Arg-85 that was demonstrated experimentally (**Figure 7**), the (Hphi)xP motif may also act in concert with the other two *cis*-elements to regulate the TTAC of VemP. This point should be addressed in the future.

Supporting result 4: The SHS and Pro-67 of VemP are involved in the regulation of arrest cancellation on the translocon (Related to Figure S7)

The reduced VemP translation arrest can be attributed to 1) a defect in the establishment of the translation arrest state (e.g., caused by an arrest motif mutation such as W143A (24)) or 2) normal arrest but enhanced arrest cancellation on the translocon (e.g., the R85W mutant, (25)). In the latter case, if VemP targeting the translocon is impaired, the translational arrest state would be stably maintained, and the arrested form of VemP would accumulate. This is indeed the case as shown by the *in vivo* assay using the His_{10}-(ΔSS)VemP-F_3M derivative, which is not targeted to the translocon due to the lack of its signal sequence (25). The *in vivo* behavior of the His_{10}-(ΔSS)VemP-F_3M with the SHS and P67A mutations was examined by IB using anti-VemP and anti-Myc antibodies and a neutral gel system that allows the detection of tRNA-attached VemP species (Figure S7). As shown previously (25), the VemP derivative with the W143A mutation generated a full-length band that cross-reacted with both antibodies (lane 5), whereas its derivative with the R85W mutation accumulated as a high-molecular-weight band that cross-reacted only with anti-VemP antibodies, as did the wild-type (lanes 4 and 6). These high-molecular-weight bands disappeared upon RNase treatment, which concomitantly generated a new band at the position of the normally arrested product (lanes 10 and 12), showing that the (ΔSS)VemP(R85W) mutant stably formed an arrested structure, similar to the wild type protein. We next examined the SHS and Pro-67 mutants in the same assay system. All QNQN, LLLLF, and P67A mutants (lanes 1–3 and 7–9) showed similar results to the wild type and the R85W mutant, indicating that their translation arrest can occur normally, strongly suggesting that the QNQN substitution in the SHS and the Ala replacement of Pro-67 enhance the arrest cancellation of VemP on the Sec translocon.

Supporting discussion 2: Considerations regarding the uneven distribution of the group 1 mutants with respect to the RGI values (Related to Figure 5C)

The number of theoretically constructible mutants that can be obtained using this method is 159,999 (20^4 - 1) and 399 (20^2 - 1) for groups 1 and 2, respectively. Since the number of mutants isolated was 23 (group 1) and 27 (group 2), the recovery of the mutants was 0.014% and 6.8%, respectively. Notably, while the properties of the isolated group 2 mutants may at least partly reflect those of all possible group 2 mutants, the isolated group 1 mutants may not accurately represent all possible group 1 mutants, especially since only a relatively small number (23) of mutants with a clear growth phenotype were selected and characterized.

Supporting result 5: Correlation between RGI values of Δ*ppiD* strains expressing VemP-Bla derivatives and relative cellular accumulation of these derivatives (Related to Figure S9).

Random and systematic mutagenesis analysis targeting the SHS in VemP revealed a significant correlation between the RGI and the hydrophobicity of the mutated SHSs (Figures 5C, 6A). To validate these results, we examined a correlation between the RGI of the Δ*ppiD* strain expressing each mutant and its cellular accumulation in the same strains by plotting the former (horizontal axis) against the latter (vertical axis) for each mutant (Figure S9). The results showed a significant relationship between the RGI value and the intracellular accumulation of VemP-Bla for both the random mutational analysis (A) and the systematic mutational analysis (B) ($R^2 > 0.6$ in both cases).

Supporting discussion 3: Relationships between the RGI of Δ*ppiD* strains expressing VemP-Bla derivatives and the properties of the SHS mutants other than hydrophobicity (Related to Figure S10).

Amino acid substitutions in the SHS affect not only the hydrophobicity of SHS but also other physical properties including its side-chain volume and α-helix propensity. We then examined the relationships between RGI and either the side-chain volume or the α-helix propensity of the mutant SHSs. The volume (A) and α-helix propensity (B) of the SHS of the mutants isolated in the random mutational analyses were calculated according to previous studies [(50) and (51), respectively] and plotted against RGI (Figure S10). The results showed very weak correlations of the RGI with both side-chain volumes ($r = -0.32$, $R^2 ≒ 0.1$) and α-helix

propensities (r = 0.19, R^2 = 0.04), suggesting that side-chain volume and α-helix propensity would not be a major determinant of RGI.

Supporting result 6: Mutational analysis of the (Hphi)xP motif (related to Figure S12A, B)

Cys scan analysis using the VemP-PhoA reporter system revealed that the Cys mutants at Gln-65 and Pro-67 showed as high PhoA activity as the Arg-85 mutants (R85C and R85W) (Figure S12A), suggesting that these residues play an important role in the VemP TTAC. We then constructed and analyzed Ala-substituted mutants of the Q^{65}SP residues using the VemP-Bla reporter. The results of all three assays shown in Figure S12A showed that the Q65A and P67A mutations destabilized the arrested state of VemP-Bla, whereas the S66A mutant did not. As the substitution with a residue with a similar property (Ser to Ala) could be a possible reason for the unchanged stability of the arrested state, we performed the same assay with mutants in which Ser-66 was replaced by four other amino acids with different properties, Phe, Arg, Lys, and Glu (Figure S12B). We found that all these mutants had a similar phenotype to the wild-type. Thus, we concluded that the Ser-66 residue is not important for the proper TTAC.

Supporting result 7: Analysis of the Q65G mutant (related to Figure S12C)

We examined the growth phenotypes of the Δ*ppiD* strain expressing VemP(Q65G)-Bla using four different transformants of the same plasmid in the liquid L-Amp medium. We found that their growth curves varied between the Δ*ppiD* strain carrying p-*vemP-bla* (small open circles, negative control) and the wild-type strain carrying the same plasmid (small closed circles, positive control) (Figure S12C, upper left panel). The growth of the same transformants also varied on the L-Amp plates (Figure S12C, lower left panel). We then introduced the Q65G mutation into VemP::FLAG-LacZα and analyzed the kinetics of its TTAC in the Δ*ppiD* strain by pulse-chase experiments. We found that the arrested state of this mutant protein was as stable as the wild-type protein (Figure 8D), which was inconsistent with the aforementioned results of VemP(Q65G)-Bla. Although the reason for this inconsistency is not clear, the results of the pulse-chase experiment, which can directly measure the kinetics of the VemP TTAC, appear to be more reliable than those of the indirect VemP-Bla assay. Thus, we concluded that the Q65G mutation has little effect on the stability of the VemP arrested state.

Supporting discussion 4: The effect of *prlA4* mutation

The *prlA4* mutation in SecY promoted the release of the VemP translation arrest in the Δ*ppiD* or *secD1* strain (Figure 1). This suggests that SecY regulates the timing of TTAC. Two possible mechanisms can explain the effect of the *prlA* mutation. 1) The PrlA4 mutant channel can allow a substrate protein with a mutated signal sequence to effectively translocate across the membrane (28). Possibly, this mutation alters the recognition of a hydrophobic region at the lateral gate, which could hinder the interaction of the VemP SHS with SecY and allow the up and down movements of VemP by Brownian motion and the rapid TTAC in both the Δ*ppiD* and *secD1* mutant strains. 2) Biochemical analysis of the PrlA4 mutant channel revealed that the PrlA4/E/G translocon exhibited an increased affinity for SecA and enhanced translocation activity (52). In this mutant strain, VemP export may be more dependent on SecA function and conversely less dependent on SecD/F function compared to the wild-type strain. In this case, even if the SHS interacts with the SecY channel, the SecA ATPase might still be able to push substrates into the channel, resulting in the failure of the transient halt of VemP translocation.

Supporting Experimental Procedures

Construction of Mutant Strains

HM6031 (NT17, Δ*ppiD*::*kan prlA4 zhd33*::Tn*10*) was constructed by co-transducing *prlA4* linked to *zhd33*::Tn*10* from HM3532 into RM3688 (27). HM6033 (NT17, Δ*ppiD*::*kan prlA*$^+$ *zhd33*::Tn*10*) was obtained as an isogenic *prlA*$^+$ strain of HM6031. The presence or absence of the *prlA4* mutation was confirmed by sequence analysis of a PCR-amplified *secY* region from these strains. HM3538 (MC4100, *prlA4* Δ*gspA*::*kan*) was constructed by transducing Δ*gspA*::*kan* from JW3285 (53) into HM3532. HM6801 (HM1742, *prlA4* Δ*gspA*::*kan*) was constructed by co-transducing *prlA4* linked to Δ*gspA*::*kan* from HM3538 into HM1742. Co-transduction of the *prlA4* mutation was also confirmed by DNA sequence analysis of a PCR-amplified *secY* region from HM1742. HM6835(HM1742, *prlA4* Δ*gspA*::*kan secD1 zaj*::Tn*10*) was constructed by co-transducing *secD1* linked to *zaj*::Tn*10* from HM3199 to HM6801. The introduction of the *secD1* mutation was confirmed by the Cs growth phenotype of the transductant and the lower accumulation level of SecD. HM6833 (HM1742, *prlA4* Δ*gspA*::*kan secD*$^+$ *zaj*:: Tn*10*) was isolated as an isogenic *secD*$^+$ strain of HM6835.

Plasmids Construction

pHM1021-*vemP(mono-Cys)-phoA* plasmids (pHM1592–pHM1616) and pHM1514 were constructed from pHM1258 (27) by site-directed mutagenesis using a pair of appropriate primers.

pHM1320 (pHM1021, *lacI*) was constructed as follows. A 1.2 kbp DNA fragment containing *lacI* was PCR-amplified from pETDuet-1 (Novagen) using the pair of primers, lacI-5' and lacI-3'. Separately, a 4 kbp vector fragment was PCR-amplified from pHM1021 (18) using the pair of primers, pHM1021-Vec-5' and pHM1021-Vec-3'. The *lacI* fragment and the pHM1021-derived fragment were connected using the In-Fusion® HD cloning kit (Clontech) to generate a pHM1021-derivative carrying the *lacI* gene. To avoid accidental mutations, the 2.2 kbp NdeI-NcoI fragment containing *lacI* was confirmed for sequence and cloned back into the same sites of pHM1021 to obtain pHM1320. pHM1532 (pHM1320-*vemP-phoA*) was constructed as follows. A NcoI-HindIII fragment containing the *vemP-phoA* gene was prepared from pHM1258 and cloned into the same sites of pHM1320 to obtain pHM1532.

pHM1507 (pSTV28(NcoI)) was constructed as follows. A unique NcoI site was introduced at the start codon of *lacZα* of pSTV28 by site-directed mutagenesis using a pair of appropriate primers to generate pHM1507. pHM1530 and pHM1586 were constructed as follows. 2 kbp NcoI-HindIII fragments containing *rpmD-rplO-secY*$^+$*-h6m* and *rpmD-rplO-secY(Cys-less)-h6m* were prepared from pNA3 (54) and pHM462 (55), respectively, and cloned into the same sites of pHM1507 to give pHM1530 and pHM1586, respectively. pHM1534 (pHM1507-*prlA4-h6m*) was constructed as follows. A 1.9 kbp DNA fragment containing the *rpmD-rplO-prlA4* genes was PCR-amplified from the genomic DNA prepared from HM5905 (a *prlA4* strain) using primers, rpsE-NcoI and secY-C(BamHI), digested with NcoI and BamHI, and cloned into the same sites of pHM1530 to obtain pHM1534. pHM1507-*secY(mono-Cys)-h6m* plasmids (pHM1588, pHM1733, pHM1735 and pHM1737) were constructed from pHM1586 by site-directed mutagenesis using a pair of appropriate primers.

pHM1721 (pHM1507-*rpmD-rplO-secY(Cys-less)-h6m-SD-ha-secE)* and pHM1722 (pHM1507-*rpmD-rplO-secY(S282C)-h6m-SD-ha-secE)* were constructed as follows. A 0.5 kbp DNA fragment containing SD-*ha-secE* was PCR-amplified from pHM991 (pBAD-*ha-secE-secY-secG*; (56)) using a pair of primers, ha-secE-5' and ha-secE-3'. Separately, pHM1664 (pBAD33-SD-*secY(Cys-less)-h6m*; lab stock) was digested with HindIII to prepare a vector fragment. The amplified fragment and the HindIII-digested vector were connected using the In-Fusion® HD cloning kit (Clontech) to generate pHM1689 (pBAD33-SD-*secY(Cys-less)-h6m*-SD-*ha-secE*). Then, an 800 bp EcoRI-HindIII* fragment containing a part of *secY* and *ha-secE* was PCR-amplified from pHM1689 using a pair of primers, secY(EcoRI)-5' and ha-secE-3'-2. The amplified fragment was cloned into the same sites of pHM1586 and pHM1588 using the In-Fusion® HD cloning kit (Clontech) to generate pHM1721 and pHM1722, respectively. pHM1727, pHM1729 and pHM1731 were constructed from pHM1721 by site-directed mutagenesis using a pair of

appropriate primers.

pHM1695 (pTV118N-*vemP::flag-lacZα*) and pHM1697 (pTV118N-*vemP::flag (T77C)-lacZα*) were constructed as follows. 5.9 kbp fragments with most of the flag-tag sequence at the both ends were PCR-amplified from pHM1258 (pHM1021-*vemP-phoA*; Miyazaki et al., 2022) and pHM1604 (pHM1021-*vemP(T77C)-phoA*) using a pair of primers, vemP-flag-P and vemP-flag-C, and circularized using the In-Fusion® HD cloning kit (Clontech) to generate pHM1680 (pHM1021-*vemP::flag-phoA*) and pHM1682 ((pHM1021-*vemP::flag (T77C)-phoA*), respectively. Then, 600 bp NcoI-HindIII fragments containing *vemP::flag* and *vemP::flag (T77C)* were PCR-amplified from pHM1680 and pHM1682, respectively, using a pair of primers, vemP-lacZα-5' and vemP-lacZα-3', and inserted into the same sites of pTV118N to generate pHM1695 and pHM1697, respectively. pTV118N-*vemP::flag (mono-Cys)-lacZα* derivatives (pHM1698-pHM1702, pHM1705-pHM1708 and pHM1712) were constructed from pHM1695 by site-directed mutagenesis using a pair of appropriate primers. pHM1725 (pTV118N-*vemP::flag (R74C, R85W)-lacZα*) was constructed from pHM1706 (pTV118N-*vemP::flag (R74C)-lacZα*) by site-directed mutagenesis using a pair of appropriate primers.

pHM1684 (pHM1021-*vemP::flag-lacZα*) was constructed as follows. A 600 bp NcoI-HindIII DNA fragment containing *vemP::flag* was amplified using a pair of primers, vemP-lacZα-5' and vemP-lacZα-3', and inserted into the same sites of pHM1021 to generate pHM1684. pHM1021-*vemP::flag-lacZα* derivatives with a single mutation (pHM1749, pHM1751–pHM1762) were constructed from pHM1684 by site-directed mutagenesis using a pair of appropriate primers.

pHM806 was constructed as follows. The phosphorylated two oligonucleotides, myc-P and myc-C were annealed and inserted into HindIII and PstI sites of pSTV28 to obtain pHM412 (pSTV28-*myc*). A 550 bp SacI-PstI fragment containing *vemP* was PCR-amplified from a *vemP*-plasmid (lab. stock) using the pair of primers, 5'-ORF-P and 5'-ORF-C and cloned into the same sites of pHM412 to obtain pHM806. pHM1505 (pSTV28-*vemP-bla*) was constructed as follows. An 820 bp PstI fragment containing *bla* was PCR-amplified from pHM1206 carrying *bla* gene encoding a Amp^R marker using a pair of primers, bla-5' and bla-3' and cloned into the PstI site of pHM806 to obtain pHM1505.

The first group of plasmids (pHM1509, pHM1510, pHM1511, pHM1513, pYI63, pYI65, pYI67, pYI69, pYI74, pYI75, pYI78, pYI80, pYI81, pYI82, pYI85, pYI85, pYI87, pYI88, pYI91, pYI92, pYI307, pYI309, pYI327 and pYI337), the second ones (pYI94, pYI96, pYI98, pYI100, pYI103, pYI104, pYI108, pYI111, pYI113, pYI115, pYI117, pYI119, pYI130, pYI132, pYI134, pYI136, pYI138, pYI140, pYI142, pYI144, pYI146 and pYI148), and the third group of plasmids (pYI120, pYI122, pYI125, pYI126 and pYI128) were constructed from pHM1505, pYI67 and pYI115, respectively, by site-directed mutagenesis using a pair of appropriate primers.

pHM1021-*vemP-f3m* derivatives with a mutation (pYI264, pYI266, pYI351, pHM1763, pHM1764, pHM1766, pHM1767, pHM1769, pHM1771, pHM1773, pHM1775, pYI333, pYI335, pHM1277, pH1301, pHM1289, and pHM1291) were constructed from pTS48 (for the first 9 mutants), pYI264 (for the middle 2 mutants) and pTS47 (for the last 4 mutants), by site-directed mutagenesis using a pair of appropriate primers used above.

pTV118N-*his_{10}-vemP(ΔSS)-f3m* derivatives with a single mutation (pYI1, pYI3, and pYI5) were constructed from pRM557 by site-directed mutagenesis using a pair of appropriate primers used above.

Plasmids carrying *vemP(Q65X)-bla* (except for Q65N and Q65K) were constructed from pHM1505 by site-directed mutagenesis using an appropriate one of the following pairs of primers, 1) 220802_VemP_Q65X_p and 220802_VemP_Q65X_m, 2) 220913_VemP_Q65X_1_p and 220913_VemP_Q65X_1_m, and 3) 220913_VemP_Q65X_2_p and 220913_VemP_Q65X_2_m. pYI307 encoding VemP(Q65N)-Bla and pYI309 encoding VemP(Q65K)-Bla were constructed from the same plasmid using a pair of appropriate primers. Plasmids carrying *vemP(P67X)-bla* (except for P67C and P67M) were constructed from pHM1505 by site-directed mutagenesis using any one of the following pair of primers, 1) 220802_VemP_P67X_p and 220802_VemP_P67X_m, 2) 220913_VemP_P67X_1_p and 220913_VemP_P67X_1_m and 3) 220913_VemP_P67X_2_p and 220913_VemP_P67X_2_m. pYI327 encoding VemP(P67C)-Bla and pYI337 encoding VemP(P67M)-Bla were constructed from the same plasmid using a pair of appropriate primers.

Table S1. Strains used in this study

Strain	Genotype	Reference
MC4100	F$^-$ *araD139* Δ*(argF-lac)U169 rpsL150 relA1 flbB5301 deoC1 ptsF25 rbsR*	(57)
CU141	MC4100, *F'lacI^{q}Z$^+$ Y$^+$*	(58)
HM1742	CU141, *ara$^+$*	(59)
NT17	MC4100, Δ*ompT*	(27)
RM3688	NT17, Δ*ppiD*::*kan*	(27)
HM3532	MC4100, *prlA4 zhd33*::Tn*10*	Lab. stock
HM6031	NT17, Δ*ppiD*::*kan prlA4 zhd33*::Tn*10*	This study
HM6033	NT17, Δ*ppiD*::*kan prlA$^+$ zhd33*::Tn*10*	This study
HM6034	NT17, *prlA4 zhd33*::Tn*10*	This study
HM3202	HM1742, *secD$^+$ zaj*::Tn*10*	(14)
HM3199	HM1742, *secD1 zaj*::Tn*10*	(14)
HM3538	MC4100, *prlA4* Δ*gspA*::*kan*	This study
HM6801	HM1742, *prlA4* Δ*gspA*::*kan*	This study
HM6833	HM1742, *prlA4* Δ*gspA*::*kan secD$^+$ zaj*::Tn*10*	This study
HM6835	HM1742, *prlA4* Δ*gspA*::*kan secD1 zaj*::Tn*10*	This study
RM3122	HM1742, Δ*ppiD*::*kan*	(25)
RM3463	HM1742, Δ*yfgM*::*kan*	(25)This study

Table S2. Plasmids used in this study

Plasmid	Vector	Genes and description	Reference or source	Relevant experiments
pHM1320		Expression vector; P_{lac}, Amp^R, *lacI*	This study	
pHM1507		Expression vector; P_{lac}, Cm^R, NcoI site is introduced	This study	
pHM1532	pHM1320	*vemP-PhoA*	This study	Fig. S2
pSTD343	pSTV29	*lacI*	(60)	Fig. 1C
pTS48	pHM1021	*vemP-3xflag-myc*	(25)	Figs. 1, 3B, C, 6B, D, 9C, S1C, S11
pST50	pHM1021	*vemP(W143A)-3xflag-myc*	(25)	Figs. 3B, 6D
pRM542	pHM1021	*vemP(R85W)-3xflag-myc*	(25)	Figs. 7, 9C
pYI264	pHM1021	*vemP(*$Q^{78}NQN^{81}$*)-3xflag-myc*	This study	Figs. 3B, 9C
pYI266	pHM1021	*vemP(*$L^{77}LLLF^{81}$*)-3xflag-myc*	This study	Figs. 3B, C
pYI351	pHM1021	*vemP(P67A)-3xflag-myc*	This study	Fig. 9C
pHM1721	pHM1507	*rpmD-rplO-secY(Cys-less)-h*$_6$*m-SD-ha-secE*	This study	Figs. 2B
pHM1722	pHM1507	*rpmD-rplO-secY(S282C)-h*$_6$*m-SD-ha-secE*	This study	Figs. 2B-D, F, S3B, S5A-E
pHM1727	pHM1507	*rpmD-rplO-secY(T404C)-h*$_6$*m-SD-ha-secE*	This study	Figs. 2F, S3B, S5D, E
pHM1729	pHM1507	*rpmD-rplO-secY(I195C)-h*$_6$*m-SD-ha-secE*	This study	Figs. 2F, S3B, S5D, E
pHM1731	pHM1507	*rpmD-rplO-secY(I191C)-h*$_6$*m-SD-ha-secE*	This study	Figs. 2F, S3B, S5D, E
pTV118N		Expression vector; P_{lac}, Amp^R	Takara Bio	
pHM1695	pTV118N	*vemP::flag-lacZα*	This study	Figs. 2B-D, F, 2S3B, S5A-E
pHM1706	pTV118N	*vemP::flag(R74C)-lacZα*	This study	Figs. 2B-D, F, S5A-E
pHM1725	pTV118N	*vemP::flag(R74C, R85W)-lacZα*	This study	Fig. S5B
pHM1712	pTV118N	*vemP::flag(E70C)-lacZα*	This study	Figs. 2D, S5C
pHM1698	pTV118N	*vemP::flag(S71C)-lacZα*	This study	Figs. 2D, S5C
pHM1705	pTV118N	*vemP::flag(H72C)-lacZα*	This study	Figs. 2D, S5C
pHM1699	pTV118N	*vemP::flag(A73C)-lacZα*	This study	Figs. 2D, S5C, E
pHM1700	pTV118N	*vemP::flag(L75C)-lacZα*	This study	Figs. 2D, S53C, E
pHM1707	pTV118N	*vemP::flag(D76C)-lacZα*	This study	Figs. 2D, S5C, E
pHM1697	pTV118N	*vemP::flag(T77C)-lacZα*	This study	Figs. 2D, S5C, E
pHM1708	pTV118N	*vemP::flag(L78C)-lacZα*	This study	Fig. 2D, S5C
pHM1701	pTV118N	*vemP::flag(A79C)-lacZα*	This study	Fig. 2D, S5C
pHM1702	pTV118N	*vemP::flag(F81C)-lacZα*	This study	Fig. 2D, S5C
pHM1550		Expression vector; P_{lac}, Spc^R	(27)	
pHM1552	pHM1550	*vemP-phoA*	(27)	Fig. S2
pHM1530	pHM1507	*rpmD-rplO-secY*$^+$*-h*$_6$*m*	(61)	Fig. S3C
pHM1534	pHM1507	*rpmD-rplO-prlA4-h*$_6$*m*	This study	Fig. S3C
pHM1586	pHM1507	*rpmD-rplO-secY(Cys-less)-h*$_6$*m*	This study	Fig. S3C
pHM1588	pHM1507	*rpmD-rplO-secY(S282C)-h*$_6$*m*	This study	Fig. S3C
pHM1733	pHM1507	*rpmD-rplO-secY(T404C)-h*$_6$*m*	This study	Fig. S3C
pHM1735	pHM1507	*rpmD-rplO-secY(I195C)-h*$_6$*m*	This study	Fig. S3C
pHM1737	pHM1507	*rpmD-rplO-secY(I191C)-h*$_6$*m*	This study	Fig. S3C
pHM1021		Expression vector; P_{lac}, Amp^R	(18)	
pHM1258	pHM1021	*vemP-phoA*	(27)	Fig. S4A
pHM1592	pHM1021	*vemP(Q65C)-phoA*	This study	Fig. S4A
pHM1593	pHM1021	*vemP(S66C)-phoA*	This study	Fig. S4A
pHM1594	pHM1021	*vemP(P67C)-phoA*	This study	Fig. S4A
pHM1595	pHM1021	*vemP(I68C)-phoA*	This study	Fig. S4A
pHM1596	pHM1021	*vemP(S69C)-phoA*	This study	Fig. S4A
pHM1597	pHM1021	*vemP(E70C)-phoA*	This study	Fig. S4A
pHM1598	pHM1021	*vemP(S71C)-phoA*	This study	Fig. S4A
pHM1599	pHM1021	*vemP(H72C)-phoA*	This study	Fig. S4A
pHM1600	pHM1021	*vemP(A73C)-phoA*	This study	Fig. S4A
pHM1601	pHM1021	*vemP(R74C)-phoA*	This study	Fig. S4A
pHM1602	pHM1021	*vemP(L75C)-phoA*	This study	Fig. S4A
pHM1603	pHM1021	*vemP(D76C)-phoA*	This study	Fig. S4A
pHM1604	pHM1021	*vemP(T77C)-phoA*	This study	Fig. S4A
pHM1605	pHM1021	*vemP(L78C)-phoA*	This study	Fig. S4A
pHM1606	pHM1021	*vemP(A79C)-phoA*	This study	Fig. S4A
pHM1607	pHM1021	*vemP(L80C)-phoA*	This study	Fig. S4A
pHM1608	pHM1021	*vemP(F81C)-phoA*	This study	Fig. S4A
pHM1609	pHM1021	*vemP(N82C)-phoA*	This study	Fig. S4A
pHM1610	pHM1021	*vemP(T83C)-phoA*	This study	Fig. S4A
pHM1611	pHM1021	*vemP(Q84C)-phoA*	This study	Fig. S4A
pHM1612	pHM1021	*vemP(R85C)-phoA*	This study	Fig. S4A
pHM1613	pHM1021	*vemP(V87C)-phoA*	This study	Fig. S4A
pHM1614	pHM1021	*vemP(W86C)-phoA*	This study	Fig. S4A
pHM1615	pHM1021	*vemP(S88C)-phoA*	This study	Fig. S4A
pHM1616	pHM1021	*vemP(H89C)-phoA*	This study	Fig. S4A
pHM1514	pHM1021	*vemP(R85W)-phoA*	This study	Fig. S4A
pRM557	pTV118N	*his*$_{10}$*-vemP(ΔSS)-3xflag-myc*	(25)	Fig. S7

pRM562	pTV118N	*his$_{10}$-vemP(ΔSS, R85W)-3xflag-myc*	(25)	Fig. S7
pRM563	pTV118N	*his$_{10}$-vemP(ΔSS, W143A)-3xflag-myc*	(25)	Fig. S7
pYI1	pTV118N	*his$_{10}$-vemP(ΔSS, Q^{78}NQN^{81})-3xflag-myc*	This study	Fig. S7
pYI3	pTV118N	*his$_{10}$-vemP(ΔSS, L^{77}LLLF^{81})-3xflag-myc*	This study	Fig. S7
pYI5	pTV118N	*his$_{10}$-vemP(ΔSS, P67A)-3xflag-myc*	This study	Fig. S7
pHM806	pSTV28	*vemP-myc*	This study	Figs. 4B-E, 5A-C, 6C, 8A, C, 9A, B, S3A-C, S9, S12A-C
pHM1505	pSTV28	*vemP-bla*	This study	Figs. 4B-E, 5A-C, 6C, 8A, C, 9A, B, S9, S12A-C
pHM1509	pSTV28	*vemP(R85W)-bla*	This study	Figs. 4B, C, 9A, B
pHM1510	pSTV28	*vemP(Q^{78}NQN^{81})-bla*	This study	Figs. 4D, E, 5A, B, 6A, C, 9A, B, S9
pHM1511	pSTV28	*vemP(P67A)-bla*	This study	Figs. 8A, C, 9A, B, S12A-C
pHM1513	pSTV28	*vemP(L^{77}LLLF^{81})-bla*	This study	Figs. 4D, E
pYI7	pSTV28	*vemP(N^{78}SNN^{81})-bla*	This study	Figs. 5A-C, S9
pYI8	pSTV28	*vemP(W^{78}WAW^{81})-bla*	This study	Figs. 5A-C, S9
pYI9	pSTV28	*vemP(R^{78}GGF^{81})-bla*	This study	Fig. 5C, S9
pYI10	pSTV28	*vemP(V^{78}RYG^{81})-bla*	This study	Fig. 5C, S9
pYI11	pSTV28	*vemP(E^{78}ATD^{81})-bla*	This study	Fig. 5C, S9
pYI12	pSTV28	*vemP(P^{78}CED^{81})-bla*	This study	Fig. 5C, S9
pYI13	pSTV28	*vemP(F^{78}DWS^{81})-bla*	This study	Fig. 5C, S9
pYI14	pSTV28	*vemP(Q^{78}LET^{81})-bla*	This study	Fig. 5C, S9
pYI15	pSTV28	*vemP(L^{78}DCK^{81})-bla*	This study	Fig. 5C, S9
pYI16	pSTV28	*vemP(R^{78}NVE^{81})-bla*	This study	Fig. 5C, S9
pYI17	pSTV28	*vemP(P^{78}NIS^{81})-bla*	This study	Fig. 5C, S9
pYI18	pSTV28	*vemP(R^{78}SDC^{81})-bla*	This study	Fig. 5C, S9
pYI19	pSTV28	*vemP(L^{78}STS^{81})-bla*	This study	Fig. 5C, S9
pYI20	pSTV28	*vemP(Y^{80}A^{81})-bla*	This study	Fig. 5C, S9
pYI21	pSTV28	*vemP(K^{80}I^{81})-bla*	This study	Fig. 5C, S9
pYI22	pSTV28	*vemP(R^{80}S^{81})-bla*	This study	Fig. 5C, S9
pYI23	pSTV28	*vemP(W^{80}N^{81})-bla*	This study	Fig. 5C, S9
pYI24	pSTV28	*vemP(L^{80}E^{81})-bla*	This study	Fig. 5C, S9
pYI25	pSTV28	*vemP(H^{80}K^{81})-bla*	This study	Fig. 5C, S9
pYI26	pSTV28	*vemP(H^{80}L^{81})-bla*	This study	Fig. 5C, S9
pYI27	pSTV28	*vemP(R^{80}Q^{81})-bla*	This study	Fig. 5C, S9
pYI28	pSTV28	*vemP(H^{80}F^{81})-bla*	This study	Fig. 5C, S9
pYI29	pSTV28	*vemP(G^{80}K^{81})-bla*	This study	Fig. 5C, S9
pYI30	pSTV28	*vemP(G^{80}K^{81})-bla*	This study	Fig. 5C, S9
pYI31	pSTV28	*vemP(R^{80}S^{81})-bla*	This study	Fig. 5C, S9
pYI32	pSTV28	*vemP(R^{80}H^{81})-bla*	This study	Fig. 5C, S9
pYI33	pSTV28	*vemP(G^{80}S^{81})-bla*	This study	Fig. 5C, S9
pYI34	pSTV28	*vemP(P^{80}H^{81})-bla*	This study	Fig. 5C, S9
pYI35	pSTV28	*vemP(S^{80}S^{81})-bla*	This study	Fig. 5C, S9
pYI36	pSTV28	*vemP(S^{80}R^{81})-bla*	This study	Fig. 5C, S9
pYI38	pSTV28	*vemP(E^{80}S^{81})-bla*	This study	Fig. 5C, S9
pYI40	pSTV28	*vemP(P^{78}NEA^{81})-bla*	This study	Fig. 5C, S9
pYI41	pSTV28	*vemP(T^{78}STE^{81})-bla*	This study	Fig. 5C, S9
pYI44	pSTV28	*vemP(P^{78}CED^{81})-bla*	This study	Fig. 5C, S9
pYI45	pSTV28	*vemP(P^{78}GYD^{81})-bla*	This study	Fig. 5C, S9
pYI46	pSTV28	*vemP(V^{78}WVL^{81})-bla*	This study	Fig. 5C, S9
pYI47	pSTV28	*vemP(L^{78}TT-^{81})-bla*	This study	Fig. 5C, S9
pYI48	pSTV28	*vemP(A^{78}TIN^{81})-bla*	This study	Fig. 5C, S9
pYI49	pSTV28	*vemP(C^{78}WFA^{81})-bla*	This study	Fig. 5C, S9
pYI50	pSTV28	*vemP(V^{78}WLV^{81})-bla*	This study	Fig. 5C, S9
pYI51	pSTV28	*vemP(T^{78}ILL^{81})-bla*	This study	Fig. 5C, S9
pYI52	pSTV28	*vemP(I^{78}VTN^{81})-bla*	This study	Fig. 5C, S9
pYI53	pSTV28	*vemP(L^{80}P^{81})-bla*	This study	Fig. 5C, S9
pYI54	pSTV28	*vemP(P^{80}S^{81})-bla*	This study	Fig. 5C, S9
pYI55	pSTV28	*vemP(P^{80}I^{81})-bla*	This study	Fig. 5C, S9
pYI56	pSTV28	*vemP(L^{80}I^{81})-bla*	This study	Fig. 5C, S9
pYI57	pSTV28	*vemP(A^{80}L^{81})-bla*	This study	Fig. 5C, S9
pYI58	pSTV28	*vemP(L^{80}G^{81})-bla*	This study	Fig. 5C, S9
pYI59	pSTV28	*vemP(L^{80}V^{81})-bla*	This study	Fig. 5C, S9
pYI60	pSTV28	*vemP(V^{80}A^{81})-bla*	This study	Fig. 5C, S9
pYI61	pSTV28	*vemP(L^{80}P^{81})-bla*	This study	Fig. 5C, S9
pYI67	pSTV28	*vemP(L^{78}LLL^{81})-bla*	This study	Fig. 6A
pYI69	pSTV28	*vemP(A^{78}LLL^{81})-bla*	This study	Fig. 6A
pYI82	pSTV28	*vemP(L^{78}ALL^{81})-bla*	This study	Fig. 6A
pYI85	pSTV28	*vemP(L^{78}LAL^{81})-bla*	This study	Fig. 6A
pYI74	pSTV28	*vemP(L^{78}LLA^{81})-bla*	This study	Fig. 6A
pYI75	pSTV28	*vemP(Q^{78}LLL^{81})-bla*	This study	Fig. 6A

pYI78	pSTV28	*vemP(L^{78}QLL^{81})-bla*	This study	Fig. 6A
pYI80	pSTV28	*vemP(L^{78}LQL^{81})-bla*	This study	Fig. 6A
pYI81	pSTV28	*vemP(L^{78}LLQ^{81})-bla*	This study	Fig. 6A
pYI104	pSTV28	*vemP(L^{78}QLQ^{81})-bla*	This study	Fig. 6A
pYI94	pSTV28	*vemP(L^{78}QQL^{81})-bla*	This study	Fig. 6A
pYI96	pSTV28	*vemP(Q^{78}LLQ^{81})-bla*	This study	Fig. 6A
pYI98	pSTV28	*vemP(Q^{78}QLL^{81})-bla*	This study	Fig. 6A
pYI100	pSTV28	*vemP(Q^{78}LQL^{81})-bla*	This study	Fig. 6A
pYI103	pSTV28	*vemP(L^{78}LQQ^{81})-bla*	This study	Fig. 6A
pYI119	pSTV28	*vemP(L^{78}ALA^{81})-bla*	This study	Fig. 6A
pYI108	pSTV28	*vemP(L^{78}AAL^{81})-bla*	This study	Fig. 6A
pYI111	pSTV28	*vemP(A^{78}LLA^{81})-bla*	This study	Fig. 6A
pYI113	pSTV28	*vemP(A^{78}ALL^{81})-bla*	This study	Fig. 6A
pYI115	pSTV28	*vemP(A^{78}LAL^{81})-bla*	This study	Fig. 6A
pYI117	pSTV28	*vemP(L^{78}LAA^{81})-bla*	This study	Fig. 6A
pYI120	pSTV28	*vemP(L^{78}AAA^{81})-bla*	This study	Fig. 6A
pYI122	pSTV28	*vemP(A^{78}LAA^{81})-bla*	This study	Fig. 6A
pYI125	pSTV28	*vemP(A^{78}ALA^{81})-bla*	This study	Fig. 6A
pYI126	pSTV28	*vemP(A^{78}AAL^{81})-bla*	This study	Fig. 6A
pYI128	pSTV28	*vemP(A^{78}AAA^{81})-bla*	This study	Fig. 6A
pYI156	pSTV28	*vemP(L^{78}QQQ^{81})-bla*	This study	Fig. 6A
pYI158	pSTV28	*vemP(Q^{78}LQQ^{81})-bla*	This study	Fig. 6A
pYI161	pSTV28	*vemP(Q^{78}QLQ^{81})-bla*	This study	Fig. 6A
pYI162	pSTV28	*vemP(Q^{78}QQL^{81})-bla*	This study	Fig. 6A
pYI164	pSTV28	*vemP(Q^{78}QQQ^{81})-bla*	This study	Fig. 6A
pHM1769	pHM1021	*vemP(L^{78}LLL^{81}) -3xflag-myc*	This study	Fig. 6B, S11
pHM1771	pHM1021	*vemP(L^{78}LAL^{81}) -3xflag-myc*	This study	Fig. 6B, S11
pHM1766	pHM1021	*vemP(Q^{78}LLL^{81}) -3xflag-myc*	This study	Fig. 6B, S11
pHM1763	pHM1021	*vemP(L^{78}LQL^{81}) -3xflag-myc*	This study	Fig. 6B, S11
pHM1764	pHM1021	*vemP(A^{78}LAL^{81}) -3xflag-myc*	This study	Fig. 6B, S11
pHM1767	pHM1021	*vemP(Q^{78}LQL^{81}) -3xflag-myc*	This study	Fig. 6B, S11
pHM1773	pHM1021	*vemP(Q^{78}LQQ^{81}) -3xflag-myc*	This study	Fig. 6B, S11
pHM1775	pHM1021	*vemP(Q^{78}QQQ^{81}) -3xflag-myc*	This study	Fig. 6B, S11
pYI204	pSTV28	*vemP(Q^{78}RQR^{81})-bla*	This study	Fig. 6C
pYI216	pSTV28	*vemP(Q^{78}KQK^{81})-bla*	This study	Fig. 6C
pYI333	pHM1021	*vemP(Q^{78}RQR^{81}) -3xflag-myc*	This study	Fig. 6D
pYI335	pHM1021	*vemP(Q^{78}KQK^{81}) -3xflag-myc*	This study	Fig. 6D
pTS47	pHM1021	*vemP-3xflag-myc* *NcoI site is absent	(24)	Fig. 7
pHM1277	pHM1021	*vemP(R85W)-3xflag-myc* *pTS47-based	This study	Fig. 7
pHM1301	pHM1021	*vemP(Q(A)R)-3xflag-myc* *pTS47-based	This study	Fig. 7
pHM1289	pHM1021	*vemP(ΔQ^{84})-3xflag-myc* *pTS47-based	This study	Fig. 7
pHM1291	pHM1021	*vemP(ΔQ^{84}-T^{85})-3xflag-myc* *pTS47-based	This study	Fig. 7
pYI63	pSTV28	*vemP(Q65A)-bla*	This study	Figs. 8A-C, S12A, C
pYI65	pSTV28	*vemP(S66A)-bla*	This study	Fig. 8, S12A
pYI87	pSTV28	*vemP(S66F)-bla*	This study	Fig. 8, S12B
pYI88	pSTV28	*vemP(S66R)-bla*	This study	Fig. 8, S12B
pYI91	pSTV28	*vemP(S66K)-bla*	This study	Fig. 8, S12B
pYI92	pSTV28	*vemP(S66E)-bla*	This study	Fig. 8, S12B
pYI226	pSTV28	*vemP(P67W)-bla*	This study	Figs. 8A, C, S12C
pYI315	pSTV28	*vemP(P67K)-bla*	This study	Figs. 8A, C, S12C
pYI318	pSTV28	*vemP(P67E)-bla*	This study	Figs. 8A, C, S12C
pYI255	pSTV28	*vemP(P67G)-bla*	This study	Figs. 8A, C, S12C
pYI248	pSTV28	*vemP(P67L)-bla*	This study	Figs. 8A, C, S12C
pYI277	pSTV28	*vemP(Q65W)-bla*	This study	Figs. 8A, C, S12C
pYI309	pSTV28	*vemP(Q65K)-bla*	This study	Figs. 8A, C, S12C
pYI223	pSTV28	*vemP(Q65E)-bla*	This study	Figs. 8A, C, S12C
pYI224	pSTV28	*vemP(Q65G)-bla*	This study	Fig. S12C
pYI222	pSTV28	*vemP(Q65L)-bla*	This study	Figs. 8A-C, S12C
pYI235	pSTV28	*vemP(Q65V)-bla*	This study	Figs. 8A, B
pYI238	pSTV28	*vemP(Q65I)-bla*	This study	Figs. 8A, B
pYI273	pSTV28	*vemP(Q65C)-bla*	This study	Figs. 8A, B
pYI232	pSTV28	*vemP(Q65M)-bla*	This study	Figs. 8A, B
pYI225	pSTV28	*vemP(Q65S)-bla*	This study	Figs. 8A, B
pYI246	pSTV28	*vemP(Q65T)-bla*	This study	Figs. 8A, B
pYI282	pSTV28	*vemP(Q65D)-bla*	This study	Figs. 8A, B
pYI307	pSTV28	*vemP(Q65N)-bla*	This study	Figs. 8A, B
pYI237	pSTV28	*vemP(Q65R)-bla*	This study	Figs. 8A, B
pYI275	pSTV28	*vemP(Q65H)-bla*	This study	Figs. 8A, B
pYI220	pSTV28	*vemP(Q65F)-bla*	This study	Figs. 8A, B
pYI221	pSTV28	*vemP(Q65Y)-bla*	This study	Figs. 8A, B
pYI245	pSTV28	*vemP(Q65P)-bla*	This study	Figs. 8A, B

pYI251	pSTV28	*vemP(P67V)-bla*	This study	Fig. 8A
pYI319	pSTV28	*vemP(P67I)-bla*	This study	Fig. 8A
pYI327	pSTV28	*vemP(P67C)-bla*	This study	Fig. 8A
pYI337	pSTV28	*vemP(P67M)-bla*	This study	Fig. 8A
pYI249	pSTV28	*vemP(P67S)-bla*	This study	Fig. 8A
pYI329	pSTV28	*vemP(P67T)-bla*	This study	Fig. 8A
pYI295	pSTV28	*vemP(P67D)-bla*	This study	Fig. 8A
pYI294	pSTV28	*vemP(P67N)-bla*	This study	Fig. 8A
pYI259	pSTV28	*vemP(P67R)-bla*	This study	Fig. 8A
pYI293	pSTV28	*vemP(P67H)-bla*	This study	Fig. 8A
pYI247	pSTV28	*vemP(P67F)-bla*	This study	Fig. 8A
pYI227	pSTV28	*vemP(P67Y)-bla*	This study	Fig. 8A
pYI314	pSTV28	*vemP(P67Q)-bla*	This study	Fig. 8A
pHM1684	pHM1021	*vemP::flag-lacZα*	This study	Fig. 8D
pHM1749	pHM1021	*vemP::flag(R85W)-lacZα*	This study	Fig. 8D
pHM1751	pHM1021	*vemP::flag(Q65A)-lacZα*	This study	Fig. 8D
pHM1752	pHM1021	*vemP::flag(Q65E)-lacZα*	This study	Fig. 8D
pHM1753	pHM1021	*vemP::flag(Q65G)-lacZα*	This study	Fig. 8D
pHM1754	pHM1021	*vemP::flag(Q65L)-lacZα*	This study	Fig. 8D
pHM1755	pHM1021	*vemP::flag(P67A)-lacZα*	This study	Fig. 8D
pHM1756	pHM1021	*vemP::flag(P67W)-lacZα*	This study	Fig. 8D
pHM1757	pHM1021	*vemP::flag(P67E)-lacZα*	This study	Fig. 8D
pHM1758	pHM1021	*vemP::flag(P67K)-lacZα*	This study	Fig. 8D
pHM1759	pHM1021	*vemP::flag(P67G)-lacZα*	This study	Fig. 8D
pHM1760	pHM1021	*vemP::flag(P67L)-lacZα*	This study	Fig. 8D
pHM1761	pHM1021	*vemP::flag(W65W)-lacZα*	This study	Fig. 8D
pHM1762	pHM1021	*vemP::flag(W65K)-lacZα*	This study	Fig. 8D

Table S3. Primers used in this study

Name	Sequence (5' to 3')
lacI-5’	ATATGTTCTGCCAAGGACACCATCGAATGGCGC
lacI-3’	AGCTAACTCACATTAATTGCGTTGCGCTCAC
pHM1021-Vec-5’	CTTGGCAGAACATATCCATC
pHM1021-Vec-3’	TAATGTGAGTTAGCTCACTCATTAG
rpsE-NcoI	TCCGTTGAAATTCTGGG
secY-C(BamHI)	GTGGGATCCTCGGCCGTAGCCTTTCAGG
ha-secE-5’	CAAACGTTAAAAGCTTAGGAGGAATTCACCATGTAC
ha-secE-3’	CAAAACAGCCAAGCTCTCAGAACCTCAGGCCAGTG
secY(EcoRI)-5’	TTTGTACCAGGAATTCGTCCGGG
ha-secE-3’-2	GCCAGTGCCAAGCTCTCAGAACCTCAGGCCAGTG
vemP-flag-P	TACAAAGACGATGACGACAAGTACTTTTCTAAGCTGCAACC
vemP-flag-C	GTCATCGTCTTTGTAGTCAGGTAAATGAGCTTTCTGATCG
vemP-LacZα-5'	AACAGACCATGGCTCAGATTTGC
vemP-LacZα-3'	GTGCCAAGCTTGCAGAAAACTGACTATTGAGTGCG
myc-P	GGAAGAACAGAAACTCATCTCCGAAGAGGACCTGCTGCGCAAACGTTAAA
myc-C	AGCTTTTAACGTTTGCGCAGCAGGTCCTCTTCGGAGATGAGTTTCTGTTCTTCCTGCA
5’-ORF-P	TTCGAGCTCATAGGAATACTATCATCG
5’-ORF-C	CTACTGCAGGGCAGAAAACTGACTATTGAG
bla-5’	GTCAGTTTTCTGCCCTGCAGCACCCAGAAACGCTGGTGAAAG
bla-3’	AGTTTCTGTTCTTCCTGCAGTTACCAATGCTTAATCAGTGAGGC
LALF(random)-p	GCAAGACTCGATACGNNSNNSNNSNNSAACACTCAACGTTGG
LALF(random)-c	CCAACGTTGAGTGTTSNNSNNSNNSNNCGTATCGAGTCTTGC
LAXXNT-p	CTCGATACGCTAGCTNNSNNSAACACTCAACGTTGG
LAXXNT-c	CCAACGTTGAGTGTTSNNSNNAGCTAGCGTATCGAG
220802_VemP_Q65X_p	GAAACGACGNNSTCTCCGATTTCTGAAAG
220802_VemP_Q65X_m	CTTTCAGAAATCGGAGASNNCGTCGTTTC
220802_VemP_P67X_p	GAAACGACGCAGTCTNNSATTTCTGAAAG
220802_VemP_P67X_m	CTTTCAGAAATSNNAGACTGCGTCGTTTC
220913_VemP_Q65X_1_p	GAAACGACGNACTCTCCGATTTCTGAAAG
220913_VemP_Q65X_1_m	CTTTCAGAAATCGGAGAGTNCGTCGTTTC
220913_VemP_Q65X_2_p	GAAACGACGTGSTCTCCGATTTCTGAAAG
220913_VemP_Q65X_2_m	CTTTCAGAAATCGGAGASCACGTCGTTTC
220913_VemP_P67X_1_p	GAAACGACGCAGTCTNACATTTCTGAAAG
220913_VemP_P67X_1_m	CTTTCAGAAATGTNAGACTGCGTCGTTTC
220913_VemP_P67X_2_p	GAAACGACGCAGTCTNSCATTTCTGAAAG
220913_VemP_P67X_2_m	CTTTCAGAAATGSNAGACTGCGTCGTTTC
220929_VemP_P67X_1_p	GAAACGACGCAGTCTNAGATTTCTGAAAG
220929_VemP_P67X_1_m	CTTTCAGAAATCTNAGACTGCGTCGTTTC
220929_VemP_P67X_2_p	GAAACGACGCAGTCTATNATTTCTGAAAG
220929_VemP_P67X_2_m	CTTTCAGAAATNATAGACTGCGTCGTTTC
RV-N	TGTGGAATTGTGAGCGG
M4C	TACGCCAGCTGGCGAAAGGG

Table S4–1. Summary of the RGI and the SHS physical properties (Group 1)

Sequence [a]	RGI [b]	Hydrophobicity ΔG [c] (kcal/mol)	Volume [d] (Å^3)	α-helix propensity [e] (kcal/mol)
LALF (in WT)	1	1.94	3.85	0.96
LALF	0.20 (± 0.11)	1.94	3.85	0.96
QNQN	1.02 (± 0.04)	−2.14	2.82	2.08
NSNN	1.04 (± 0.02)	−1.53	2	2.45
WWAW	0.25 (± 0.13)	5.24	5.41	1.47
RGGF	1.03 (± 0.03)	0.16	2.62	2.75
VRYG	0.77 (± 0.01)	−0.09	3.47	2.35
EATD	1.06 (± 0.05)	−3.7	2.29	1.75
FDWS	1.0 (± 0)	1.48	3.88	2.22
QLET	0.96 (± 0.03)	−2.32	3.4	1.66
LDCK	1.03 (± 0.02)	−1.56	3.39	1.84
RNVE	1.01 (± 0.01)	−3.46	3.47	1.87
PNIS	0.85 (± 0.02)	−0.83	2.315	4.72
RSDC	1.03 (± 0.04)	−2.07	2.65	2.08
LSTS	0.95 (± 0.06)	0.02	2.36	1.87
PNEA	1.02 (± 0.02)	−3.2	2.025	4.21
TSTE	0.99 (± 0)	−2.57	2.37	2.22
PCED	1.01 (± 0.01)	−3.6	2.245	4.93
PGYD	0.98 (± 0.03)	−0.89	2.255	5.38
VWVL	0.23 (± 0.13)	2.13	4.49	1.92
LTT-	0.83 (± 0.11)	−0.28	2.89	2.18
ATIN	0.2 (± 0.02)	−0.56	2.59	1.72
CWLV	0.31 (± 0.02)	2.91	3.88	1.71
VWLV	0.21 (± 0.05)	2.13	4.49	1.92
TILL	0.25 (± 0.07)	1.15	3.94	1.49
IVTN	0.36 (± 0.10)	−0.46	3.12	2.33

[a]: Amino acid sequence of the 78 – 81 region of VemP-Bla derivatives.

[b]: Relative growth index (RGI) of Δ*ppiD* cells expressing VemP-Bla derivatives was calculated according to the formula shown in Figure 5C. The mean values are shown with S.D. (N=2, biological replicates)

[c]: Gibbs energy (from bilayer to water) of 5 residues (78 – 82) of VemP-Bla were calculated by using Wimley and White scale (1996).

[d]: Side-chain volumes of 4 residues (78 – 81) of VemP-Bla mutants isolated were calculated according to the literature (50).

[e]: α-helix propensity of 4 residues (78 – 81) of VemP-Bla mutants was calculated according to the literature (51).

Table S4–2. Summary of the RGI and the SHS physical properties (Group 2)

Sequence [a]	RGI [b]	Hydrophobicity ΔG [c] (kcal/mol)	Volume [d] (Å^3)	α-helix propensity [e] (kcal/mol)
LF (in WT)	1	1.94	3.85	0.96
LF	0.20 (± 0.11)	1.94	3.85	0.96
YA	0.64 (± 0.16)	1.02	3.09	0.74
KI	0.92 (± 0.06)	−0.43	3.72	0.88
RS	0.42 (± 0.05)	−0.69	2.98	0.92
WN	0.54 (± 0.12)	1.68	3.68	1.35
LE	1.01 (± 0.01)	−1.21	3.36	0.82
HK	0.96 (± 0.03)	−0.91	3.57	0.96
HL	0.6 (± 0.12)	0.64	3.49	0.61
RQ	0.66 (± 0.15)	−1.14	3.51	1.08
HF	0.84 (± 0.06)	0.42	3.7	1.36
GK	1.02 (± 0.01)	−0.75	2.64	1.47
SE	1.08 (± 0.09)	−1.9	2.57	1.11
RS	0.44 (± 0.04)	−0.69	2.98	0.92
RH	0.47 (± 0.03)	−0.73	3.62	1.03
GS	0.63 (± 0.08)	0.11	1.77	1.71
PH	0.23 (± 0.04)	−0.37	2.705	3.98
SS	0.33 (± 0.09)	−0.01	2.06	1.21
SR	0.2 (± 0.06)	−0.69	2.98	0.92
ES	0.75 (± 0.12)	−1.9	2.57	1.11
LP	0.24 (± 0.12)	0.36	2.855	3.58
LS	0.27 (± 0.05)	0.68	2.85	0.92
PI	0.23 (± 0.02)	0.11	2.855	3.78
LI	0.02 (± 0.02)	1.12	3.64	0.83
AL	0.21 (± 0.02)	0.64	2.84	0.42
LG	0.34 (± 0.04)	0.8	2.56	1.42
LV	0.11 (± 0.02)	0.74	3.37	1.03
VA	0.25 (± 0.03)	0.01	2.57	0.82
LP	0.06 (± 0.06)	0.36	2.855	3.58

[a]: Amino acid sequence of 80 – 81 of VemP-Bla derivatives.

[b-e]: as described in Table S4-1.
